# Discovery of inhibitors of protein tyrosine phosphatase 1B contained in a natural products library from Mexican medicinal plants and fungi using a combination of enzymatic and *in silico* methods**

**DOI:** 10.3389/fphar.2023.1281045

**Published:** 2023-10-31

**Authors:** Miriam Díaz-Rojas, Martin González-Andrade, Rodrigo Aguayo-Ortiz, Rogelio Rodríguez-Sotres, Araceli Pérez-Vásquez, Abraham Madariaga-Mazón, Rachel Mata

**Affiliations:** ^1^ Facultad de Química, Universidad Nacional Autónoma de México, Mexico City, Mexico; ^2^ Facultad de Medicina, Universidad Nacional Autónoma de México, Mexico City, Mexico; ^3^ Instituto de Química Unidad Mérida and Instituto de Investigaciones en Matemáticas Aplicadas y en Sistemas Unidad Mérida, Universidad Nacional Autónoma de México, Mexico City, Mexico

**Keywords:** PTP1B, chemical space, scaffold hops, canophyllol, *E*/*Z* vermelhotin

## Abstract

This work aimed to discover protein tyrosine phosphatase 1B (PTP1B) inhibitors from a small molecule library of natural products (NPs) derived from selected Mexican medicinal plants and fungi to find new hits for developing antidiabetic drugs. The products showing similar IC_50_ values to ursolic acid (UA) (positive control, IC_50_ = 26.5) were considered hits. These compounds were canophyllol (**1**), 5-*O*-(β-D-glucopyranosyl)-7-methoxy-3′,4′-dihydroxy-4-phenylcoumarin (**2**), 3,4-dimethoxy-2,5-phenanthrenediol (**3**), masticadienonic acid (**4**), 4′,5,6-trihydroxy-3′,7-dimethoxyflavone (**5**), *E*/*Z* vermelhotin (**6**), tajixanthone hydrate (**7**), quercetin-3-*O*-(6″-benzoyl)-β-D-galactoside (**8**), lichexanthone (**9**), melianodiol (**10**), and confusarin (**11**). According to the double-reciprocal plots, **1** was a non-competitive inhibitor, **3** a mixed-type, and **6** competitive. The chemical space analysis of the hits (IC_50_ < 100 μM) and compounds possessing activity (IC_50_ in the range of 100–1,000 μM) with the BIOFACQUIM library indicated that the active molecules are chemically diverse, covering most of the known Mexican NPs’ chemical space. Finally, a structure–activity similarity (SAS) map was built using the Tanimoto similarity index and PTP1B absolute inhibitory activity, which allows the identification of seven scaffold hops, namely, compounds **3**, **5**, **6**, **7**, **8**, **9**, and **11**. Canophyllol (**1**), on the other hand, is a true analog of UA since it is an SAR continuous zone of the SAS map.

## 1 Introduction

More than 474 million people worldwide live with type 2 diabetes mellitus (T2DM), characterized by chronic hyperglycemia due to insulin resistance and pancreatic β-cell dysfunction. Furthermore, in 2021, the International Diabetes Federation has estimated that over 6 million deaths yearly are due to T2DM (IDF, 2021). To control high blood glucose levels in diabetic patients, in addition to a healthy lifestyle, drug treatments and/or insulin are prescribed. Nonetheless, in some countries such as in Mexico, a large segment of the population uses medicinal plants for treating diabetes, alone or in combination with allopathic medicines.

The global prevalence of diabetes has prompted the search for novel and more efficacious therapeutic agents. In this regard, a better understanding of the complicated mechanisms involved in T2DM has inspired the pursuit of new compounds targeting new receptors and crucial enzymes involved in glucose homeostasis (ADA, 2022). Protein tyrosine phosphatase 1B (PTP1B) shows promise among these enzymes. PTP1B is a critical enzyme in the dephosphorylation of the insulin receptor and its downstream signaling components. The relevance of this enzyme in the pathophysiology of diabetes has been demonstrated by the genetic deletion of PTP1B in mice; PTP1B-deficient mice remain insulin sensitive on a high-fat diet when compared with the wild-type (Na et al., 2016; Kanwal et al., 2022).

PTP1B is composed of three domains, an *N*-terminal catalytic domain (1–300) that contains the crucial Cys215, Asp181, and Gln262 residues; a regulatory domain (301–400) that is proline-rich and confers substrate specificity to PTP1B; and finally, a *C*-terminal domain (401–435) that is involved in the binding to the plasmatic reticulum and is intrinsically disordered (Tonks, 2003; Song et al., 2017; Liu et al., 2022; Quy et al., 2022). Since the non-receptor protein tyrosine phosphatase family has a highly conserved catalytic site (∼75% of sequence similarity), searching for selective allosteric inhibitors is crucial in drug discovery.

To discover new hit compounds that inhibit PTP1B, screening libraries based on natural products, mostly from antidiabetic plants, is an excellent and straightforward strategy (Singh et al., 2022). In addition, natural product libraries occupy a greater area of chemical space than synthetic compounds, reduce screening costs, and increase the speed of finding appropriate hits (Lahlou, 2007; Harvey et al., 2010). Finally, it is important to point out that a few natural products and some chemical derivatives, such as trodusquemine from the dogfish shark, ertiprotafib, and JTT-551, have entered clinical trials (Kazakova et al., 2022).

Based on the aforementioned considerations, this work aimed to 1) identify molecules from a small molecule library of natural products obtained from Mexican medicinal plants and fungi that might inhibit PTP1B by *in-house* enzymatic screening; 2) explore their chemical space; and 3) identify the scaffold hops and activity with the structure–activity similarity (SAS) map. Altogether, all these ensure a high success rate of finding new PTP1B inhibitors with chemically original or similar structures and increase the probability of identifying hit compounds.

## 2 Materials and methods

### 2.1 PTP1B inhibition assay

A spectrophotometric and colorimetric method was used to detect the inhibitory activity of 99 *in-house* compounds on *h*PTP1B_1-400_ (Rangel-Grimaldo et al., 2020). The analyses were carried out in triplicate; the test materials and positive control (ursolic acid) were dissolved in DMSO and buffer solution. Ursolic acid was used as a reference since it has been widely reported as one of the best allosteric PTP1B inhibitor by different research groups (Liu et al., 2022; Quy et al., 2022). The compounds were initially tested in concentrations between 20 and 1,000 μM, enzyme (66 nM) and buffer [Tris-HCl 50 mM, pH 6.8] solution, and 0.125 mM of 4-nitrophenyl phosphate (4-NP, Sigma-Aldrich, St. Louis, MO, United States) was incubated at room temperature for 20 min as previously described. At the end of the incubation, the reaction was terminated by adding 5 μL of NaOH (10 M); then, the absorbance was measured at 405 nm (t20). The inhibitory activity was determined as a percentage compared to the blank (Buffer) according to the following equation:

$${\%Inhibition}_{PTP1B}=\left(1-\frac{A_{405\left(s\right)}}{A_{405\left(b\right)}}\right)\times 100\%,$$ (1)

where A_405(s)_ is the ΔA of the sample (A_t20_ − A_t0_), A_405(b)_ is the ΔA of the blank obtained in the same manner as that of the sample. Using the data obtained, the enzyme inhibition curve was plotted, for which the average percentage of inhibition was plotted as a function of the inhibitor concentration. To calculate the IC_50_, non-linear regression was performed using the Origin 8.0 software (OriginLab Corporation, 2022). For calculating the IC_50_, the compounds showing a higher percent of inhibition at 20 μM were tested at different concentrations, as shown in Supplementary Figure S1.

### 2.2 *In-house* library of natural products

The tested *in-house* compounds (1–99) were isolated from medicinal plants and fungi, as mentioned in Supplementary Table S1, which includes the natural sources and pertinent references. These compounds belong to a small molecule library of natural products from the Department of Pharmacy, School of Chemistry, UNAM.

#### 2.2.1 Preparation of natural products databases

The isomeric SMILES codes of the most active tested compounds were generated using the Open Babel toolbox (O’Boyle et al., 2011). For chemical space characterization, we included our *in-house* library (most active NPs) and 423 Mexican natural products from the BIOFACQUIM database (Pilón-Jiménez et al., 2019).

#### 2.2.2 Molecular fingerprints

Six molecular fingerprints (FPs) were calculated for the natural products database employing the MayaChemTools (Sud, 2016), rcdk (Guha, 2007), and RDKit (Landrum, 2022) packages (Supplementary Figure S2). These FPs were further used to compute the Tanimoto similarity index from the similarity matrix analysis. The Tanimoto similarity cumulative distribution (TSD) plots were generated to select the best FP representation for the tested compounds and the BIOFACQUIM library (Supplementary Figure S2). The PubChem bit-string (881 bits) was computed for the natural products database chemical space representation using the Rcpi (Cao et al., 2015) module of the R software (R Core Team, 2020).

In the case of the most active NPs, 25 molecular FPs were calculated, and the same methodology was followed to select the best FP representation and plot the SAS map (Supplementary Figure S14).

The bit-string was dimensionally reduced by employing the t-distributed stochastic neighbor embedding (t-SNE) projection computed using the scikit-learn Python library (Pedregosa et al., 2011) (perplexity: 40; iterations: 3,000). All plots from the chemoinformatics analysis were generated using Gnuplot 5.0 (Williams et al., 2017).

### 2.3 Kinetic analysis

The conditions for preparing reagents (enzyme and substrate), incubation, and analysis of results were the same as that described in Section 2.1 (Jiménez-Arreola et al., 2020; Díaz-Rojas et al., 2021). Additional steps in the methodology are described below.

To characterize the type of inhibition exerted by **1**, **3**, and **6** on PTP1B, the hydrolysis of 4-NP at the sub-saturating state and increasing concentrations of each compound was measured. An enzyme saturation curve was prepared with a 4-NP stock solution (10 mM); then, a series of curves with variable 4-NP concentrations and at least five fixed concentrations of the inhibitors were built; by considering the IC_50_ value as the midpoint, the type of inhibition of **1**, **3**, and **6** was estimated; each point of the curves was obtained in triplicate. The compound concentration yielding 50% activity inhibition was interpolated from the results by fitting into a general inhibition model. The resulting set of activities was fitted globally using the non-linear regression algorithm of Levenberg–Marquardt as implemented in the Gnuplot (Williams et al., 2017). Each group was fitted to the kinetic equations for linear competitive, linear uncompetitive, linear non-competitive (classic), linear mixed type, parabolic competitive, parabolic uncompetitive, and parabolic non-competitive inhibition mechanisms. The best fit was ascertained by the reduced chi-squared (χ^2^) value, residual distribution, and uncertainty in the parameter estimates. The best description of the inhibition data for compounds **1**, **3**, and **6** was obtained with Eq. 2, Eq. 3, and Eq. 4, respectively, where V_MAX_, K_M_, and K_Icu_, K_IC_, and K_IU_ are the maximum velocity, Michaelis constant for the substrate 4-NP, and inhibition constants for each type of inhibition, respectively.

$$v_{0}=\frac{V_{\mathrm{MAX}}\left[4NP\right]}{K_{M}\left(1+\frac{\left[I\right]}{K_{\text{Icu}}}\right)+\left[4NP\right]\left(1+\frac{\left[I\right]}{K_{\text{Icu}}}\right)},$$ (2)

$$v_{0}=\frac{V_{\mathrm{MAX}}\left[4NP\right]}{K_{M}\left(1+\frac{\left[I\right]}{K_{\text{IC}}}\right)+\left[4NP\right]\left(1+\frac{\left[I\right]}{K_{\text{IU}}}\right)},$$ (3)

$$v_{0}=\frac{V_{\mathrm{MAX}}\left[4NP\right]}{K_{M}\left(1+\frac{\left[I\right]}{K_{\text{IC}}}+\frac{{\left[I\right]}^{2}}{{K_{IC2}}^{2}}\right)+\left[4NP\right]}.$$ (4)

### 2.4 Structure–activity similarity

The SAS map of the most active compounds tested against PTP1B was constructed using the absolute value of the pIC_50_ difference (|∆Activity|) and the UA similarity calculated with the atom type fingerprints (Supplementary Figure S14), following our previous work methodology (Santiago et al., 2021). The SAS map can only be constructed using the absolute value of the pIC_50_ difference (|∆ Activity|) against ursolic acid and the UA similarity of Tanimoto (Bajusz et al., 2015).

### 2.5 Structural model of PTP1B_1-400_

A structural model of the *h*PTP1B_1-400_ protein was obtained from the AlphaFold Protein Structure Database developed by DeepMind and EMBL-EBI (https://alphafold.ebi.ac.uk/). The UniProt code P18031 corresponds to the PTPN1 gene, which codes for human PTP1B that is among the proteins in the database predicted by AlphaFold (code: Q9PT91). The pdb file was downloaded from the following link: https://alphafold.ebi.ac.uk/entry/P18031 (David et al., 2020). The coordinates of this model were prepared to perform a molecular dynamics (MD) simulation using the LEAP module from AmberTools 2021. The structure was submitted to the following procedure: hydrogens were added using the LEAP module with the leaprc.protein.ff19SB force field; K^+^ counter ions were also included to neutralize the system. The protein was solvated in an octahedral box of explicit TIP3P model water molecules localizing the box limits at 12 Å from the protein surface. MD simulations were performed at 1 atm and 315 K, and maintained with the Berendsen barostat and thermostat, respectively, using periodic boundary conditions and particle mesh Ewald sums (grid spacing of 1 Å) for treating long-range electrostatic interactions with a 10-Å cutoff for computing direct interactions. The SHAKE algorithm was used to satisfy bond constraints, using a time step of two femtoseconds (fs) to integrate Newton’s equations as recommended in the Amber package. All calculations were made using the graphics processing unit (GPU)–accelerated MD engine in Amber (pmemd.cuda), a program package that runs entirely on CUDA-enabled GPUs (Case et al., 2005, 2012). The protocol minimized the initial structure, followed by 50-picosecond (ps) heating and pressure equilibration at 315 K and 1.0 atm pressure, respectively. Finally, the system was equilibrated with 500 ps before starting the production of MD. The production of the MD consisted of 100 nanoseconds (ns). The frames were saved at 10-ps intervals for subsequent analysis. All analyses were done using CPPTRAJ (Roe and Cheatham, 2013). Root mean square deviations (RMSDs) and root mean square fluctuations (RMSFs) were calculated, considering the C, Cα, and N. The charts were built using OriginPro 9.1, and the trends were adjusted with smooth function processing (lowess span method).

#### 2.5.1 Molecular docking

The docking analysis was done using the structural model of *h*PTP1B_1-400_ obtained after MD simulation. Structures **1–11** were constructed and minimized using the Avogadro software (Hanwell et al., 2012). The AutoDockTools 1.5.4 was used to prepare the pdb files of the protein and compounds. Polar hydrogen atoms and Kollman united-atom partial charges were added to the protein structures. By contrast, Gasteiger–Marsili charges and rotatable groups were automatically assigned to the structures of the ligands. We used AutoDock Vina to carry out the docking, covering the entire enzyme. The grid box size was 126 Å × 126 Å × 126 Å in the x, y, and z dimensions and the central coordinates of 54.94, 68.66, and 63.45 for x, y, and z, respectively, with exhaustiveness of 8. The best conformational states were visualized by using PyMOL version 2.4.0 and Maestro version 5.3.156 (DeLano, 2004).

In the case of compound **6**, the geometric isomers and their more stable conformers (**6a1**, **6a2**, **6b1**, and **6b2**) were optimized using SQM-MP7 methodology with implicit aqueous solvation and finally converted to PDBQT format. The three-dimensional model of PTP1B was the same as that used in the aforementioned compounds. A wide box comprised the whole putative active site and its neighborhood within 7 Å. Several docking rounds were performed using AutoDock Vina 1.2.3 (Eberhardt et al., 2021) to collect at least 160 docking poses for each compound. The compounds were clustered using the Clustering Tool plugin for VMD (Humphrey et al., 1996), using a 1-Å cutoff, and the average Vina docking energy of each cluster was obtained from Vina logs. Each cluster’s RMSD was calculated using one arbitrary pose as a reference. Clusters having one or more poses with docking energy higher than −5.3 kcal/mol were ignored because the more stable ligand–protein complexes for compound **6** presented more negative values, therefore all values above −5.3 kcal/mol were disregarded. The docking events with lower energy fell into two neighboring but well-separated binding pockets designated as I and II. For each binding pocket, the conformation with lower docking energy and lower RMSD was considered more likely to represent the real conformation, although docking poses occupying each pocket were considered.

### 2.6 Medicinal chemistry and ADMET predictions of the most active NPs

The medicinal chemistry (MC) properties analyzed included the presence of toxicophore groups, undesirable structural fragments (BRENK alerts), the absence of promiscuous moieties (PAINS), and the Lipinski and Golden Triangle rules. All these properties were calculated using the SwissADME or ADMETlab 2.0 (Sander et al., 2015; Daina et al., 2017; Xiong et al., 2021). Metabolism (inhibition of CYP1A2, CYP2C19, CYP2C9, CYP2D6, and CYP3A4), passive human gastrointestinal (GI) absorption, blood–brain barrier (BBB) permeation (using the BOILED-Egg), and drug-likeness (bioavailability radar) were predicted using the SwissADME web platform. Excretion using clearance (CL ≥ 15, mL/min) and short half-life (T1/2) by the probability ≤1 and other absorption parameters such as skin permeability (log Kp, cm/s, positive value), water solubility (using SILICOS-IT), Caco-2 cell (permeability ≥ −5.15), and distribution (PPB ≤90%; VD, 0.04–20 L/g; Fu ≥ 20%) were calculated using the ADMETlab 2.0 online tool.

The toxicological properties of the active compounds were projected using the DataWarrior v.5.5.0 (Sander et al., 2015) and ICM v. 3.9 (Molsoft). Potential genotoxicity, carcinogenicity, rat oral acute (ROA) toxicity, inhibition of the cardiac potassium channel (hERG), human hepatotoxicity (H-HT), drug-induced liver injury (DILI), renal toxicity, and reproductive effects were analyzed (probability ≤1). In addition, the prediction of the medium lethal dose (DL50, mg/kg) in rats (acute oral toxicity) was achieved using the Toxicity Estimation Software Tool (TEST, EPA, version 5.1).

The properties calculated were compared to those known inhibitors of PTP1B, which included UA (pIC_50_ = 4.66), trodusquemine (T, pIC_50_ = 6.0), ertiprotafib (E, pIC_50_ = 4.70), and metformin (M) (Lantz et al., 2010; Kumar et al., 2020).

### 2.7 Acute oral toxicity in mice

The potential acute toxicity of compound **6** was determined according to the Lorke’s method (Lorke, 1983). The evaluation was performed following the Official Mexican Norm for Laboratory Animal Care and Use (NOM-062-ZOO-1999), with international conventional codes for laboratory animal use, and approved by the Institutional Committee for Care and Use of Laboratory Animals (CICUAL-FQ), Facultad de Química, UNAM (FQ/CICUAL/440/21). Eight-week-old male ICR mice (25–36 g) were adjusted to laboratory conditions. At the end of the experiments, the mice were euthanized by hypoxia in a CO_2_ chamber.

Compound **6** was fed by an intragastric route in two independent phases. In each case, 12 mice were divided into four groups (n = 3). In the first stage, the animals were treated with 10, 100, and 1,000 mg/kg; in the second phase, 1,600, 2,900, and 5,000 mg/kg were administered. The control animals were fed 0.05% Tween 80^®^ in saline solution. The weight of the animals was measured daily for 14 days at each stage. At the end of the test, all animals were euthanized to obtain the lungs, heart, kidneys, and liver to detect macroscopic organ injury. The mice were observed to identify acute toxic effects, changes in behavioral patterns, or mortality.

## 3 Results and discussion

### 3.1 Inhibition of PTP1B and chemical space of NPs

A screening using *h*PTP1B_1-400_ of a small customized (*in-house*) NP library containing 99 compounds, mostly from medicinal plants, was performed. The molecules were selected by the following criteria: ethnopharmacological background of the plant source, good physicochemical properties such as solubility, and the availability of the compounds for future research. The preliminary screening process at two different concentrations (20 and 1,000 μM) allowed the identification of those molecules that showed 90% inhibition or more at 20 μM. Thereafter, the IC_50_ values of these molecules were calculated. The results allowed us to identify 47 inhibitory NPs, with IC_50_ values ranging from 30 to 1,000 μM, and the most active were compounds **1–11**, whose activity gap ranged between 30 and 100 μM. Ursolic acid was used as the reference since it has been widely reported as one of the best allosteric PTP1B inhibitor by different research groups (Liu et al., 2022; Quy et al., 2022). Next, their pIC_50_ values [the negative log of the medium inhibitory concentration (IC_50_)] (Table 1, Supplementary Table S2, and Supplementary Figure S1) were calculated; the pIC_50_ value is a more reliable parameter to compare the potency of the different compounds tested at the same molar levels, and it is commonly used in chemoinformatics studies; furthermore, the pIC_50_ value covers both micro- and millimolar potencies (Kalliokoski et al., 2013; Thakur et al., 2022).

**TABLE 1:** *In vitro* inhibitory activity against PTP1B of most active compounds of the natural product library.

Compound	pIC_50_^[a]^	ΔA^[b]^	Compound	pIC_50_^[a]^	ΔA^[b]^
1	4.48	0.2	7	4.08	0.6
2	4.39	0.3	8	4.06	
3	4.32		9	4.05	
4	4.31	0.4	10	4.05	
5	4.24		11	4.00	0.7

^[a]^Negative log of the medium inhibitory concentration (IC_50_) in Molar.

^[b]^Activity difference between UA and the compound.

**FIGURE 1:**
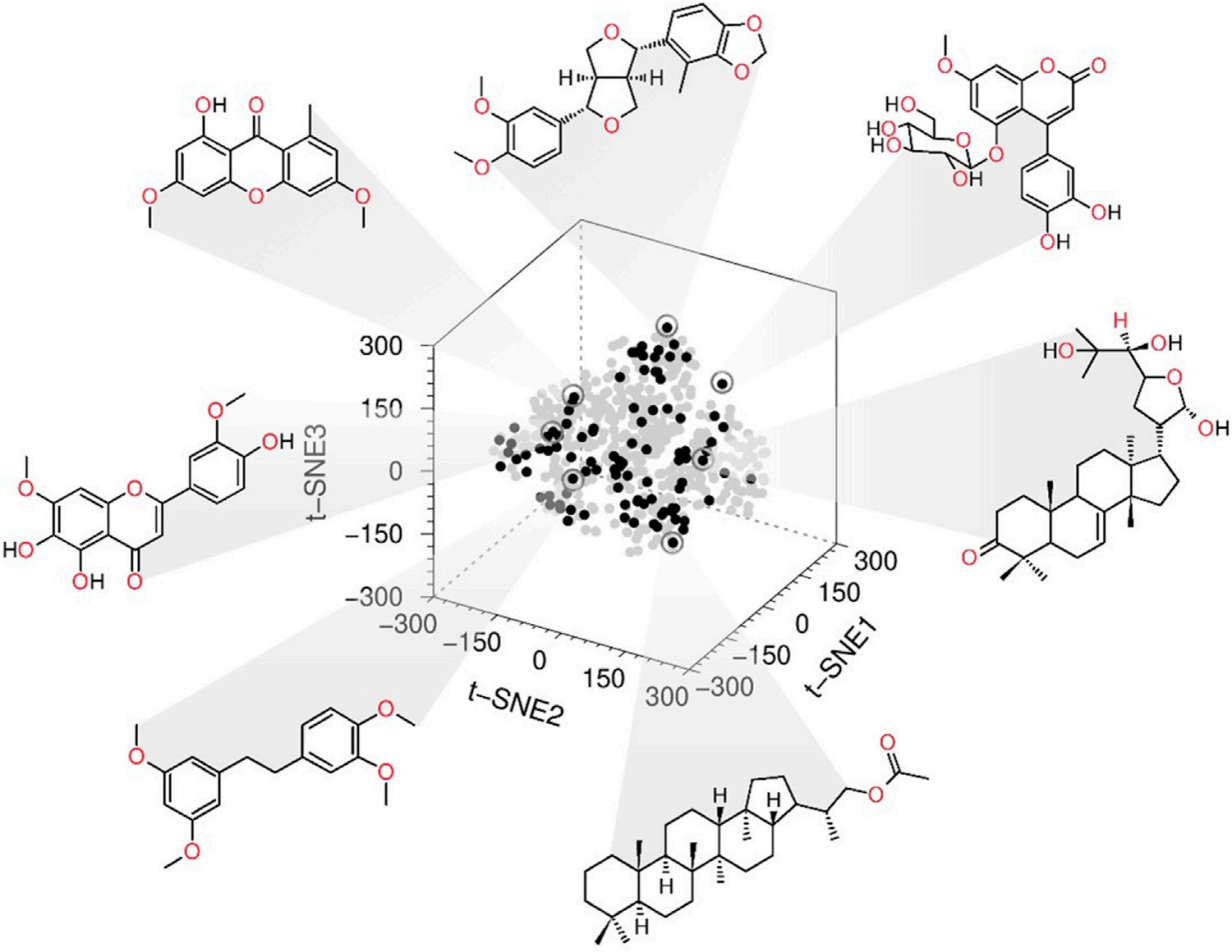
Chemical space 3D representation: the black spots are the most active compounds tested, and gray spots represent the Mexican natural products database BIOFACQUIM.

We use the same fingerprints (NCBI, 2009) to represent the active NPs (**1–11**, **14**, **15**, **18**, **25**, **32**, **34**, **40**, **41**, **43**, **46**, **47**, **49–51**, **53**, **54**, **56**, **58**, **63**, **64**, **67**, **73**, **77**, **82**, **84–86**, and **90–94**), UA and the Mexican NPs database (BIOFACQUIM https://zinc15.docking.org/catalogs/biofacquimnp/) (Pilón-Jiménez et al., 2019). Then, the 3D chemical space projection obtained from the *t*-SNE analysis of the computed FP demonstrated that the compounds with the best inhibitory activity are chemically diverse. Furthermore, the active compounds of the *in-house* library were distributed entirely in the chemical space of BIOFACQUIM (Figure 1 and Supplementary Figure S2).

**FIGURE 2:**
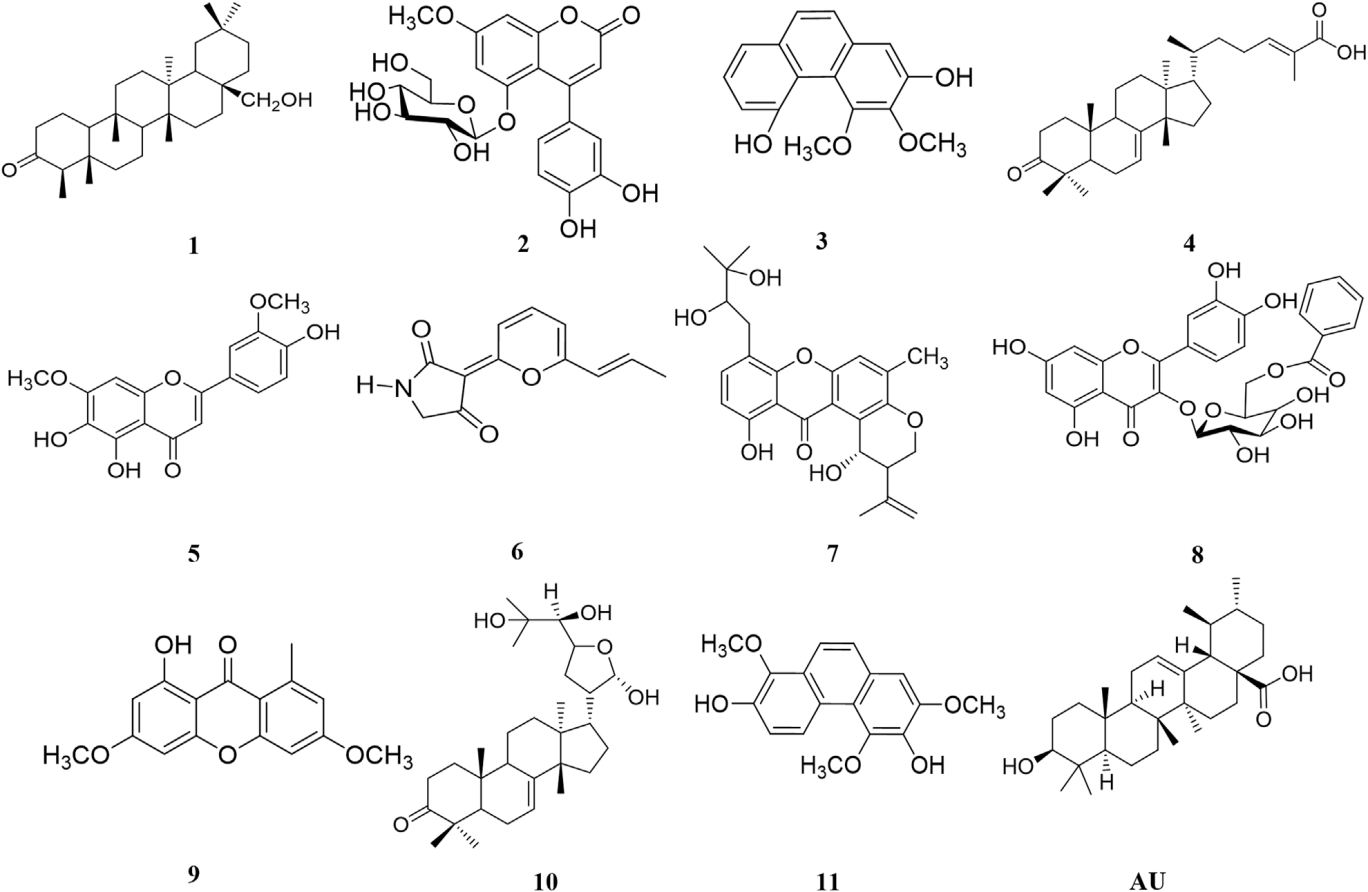
Compounds showing pIC_50_ in the same magnitude order as that of ursolic acid (UA).

Among the 47 most active NPs, compounds **1–11** (Figure 2; Table 1 and Supplementary Figure S2) presented similar pIC_50_ values to UA (pIC_50_ = 4.66 ± 0.004), therefore they were considered hits (Sharma et al., 2020). These compounds included canophyllol (**1**), a friedelane triterpenoid (Calzada et al., 1991); 5-*O*-(β-D-glucopyranosyl)-7-methoxy-3′,4′-dihydroxy-4-phenylcoumarin (**2**) (Guerrero-Analco et al., 2005); 3,4-dimethoxy-2,5-phenanthrenediol (**3**) (Estrada et al., 1999); masticadienonic acid (**4**); a tirucallane (Rivero-Cruz et al., 2010); 4′,5,6-trihydroxy-3′,7-dimethoxyflavone (**5**) (Salinas-Arellano et al., 2020); *E*/*Z*-vermelhotin (**6**) (Leyte-Lugo et al., 2012); tajixanthone hydrate (**7**) (Figueroa et al., 2009); quercetin-3-*O*-(6″-benzoyl)-β-D-galactoside (**8**) (Flores-Bocanegra et al., 2015); lichexanthone (**9**) (Rojas et al., 2000); the protolimonoid melianodiol (**10**) (Jimenez et al., 1998); and the phenanthrene confusarin (**11**) (Morales-Sánchez et al., 2014). All but the fungal compounds **6**, **7**, and **9** were isolated from plants with reputed antidiabetic properties. The natural sources of these compounds are given in Supplementary Table S1. All compounds but **5** were not previously evaluated against PTP1B.

### 3.2 Kinetic analysis of selected compounds

**FIGURE 3:**
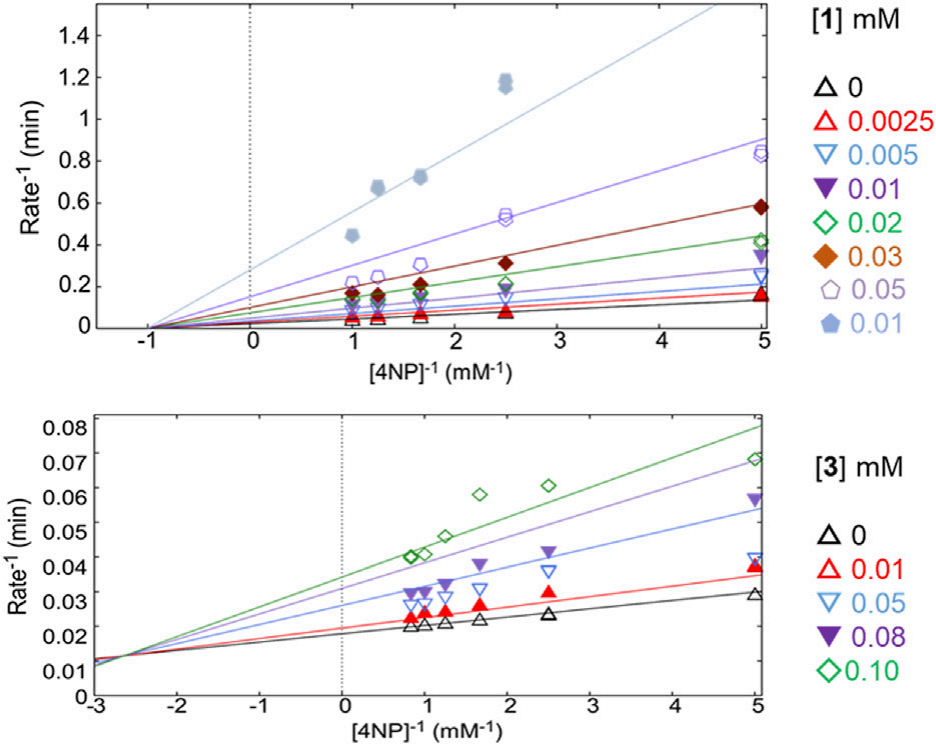
Double-reciprocal plots of PTP1B inhibition at different concentrations of compounds **1** and **3**.

**FIGURE 4:**
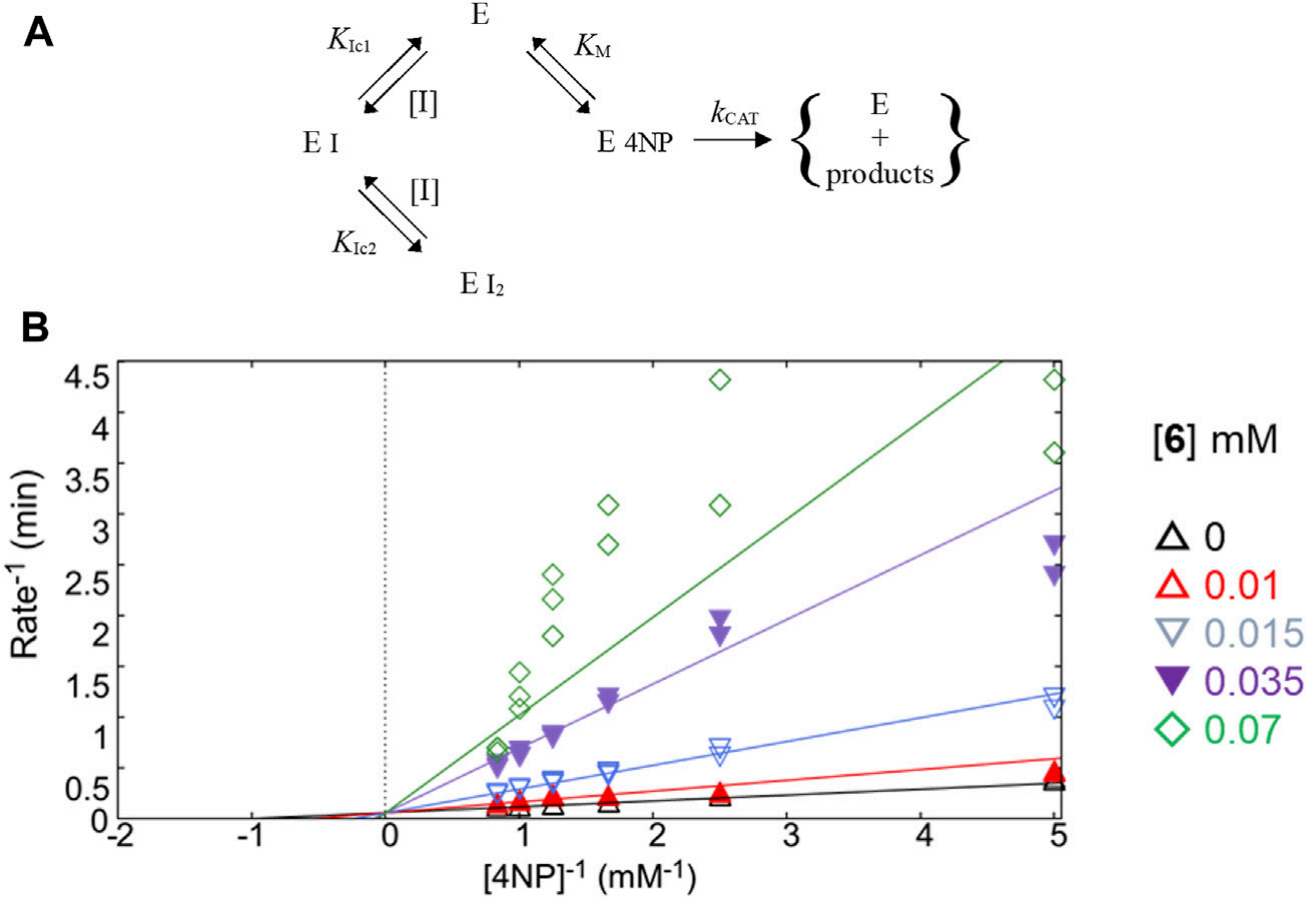
Study of kinetic inhibition for **6**. **(A)** Equilibria for the parabolic competitive inhibition. **(B)** Inhibition patterns of PTP1B fitted to a global total competitive parabolic inhibition.

Compounds **1**, **3**, and **6** were selected for kinetic analysis due to their sufficient availability and good inhibitory activity against PTP1B (Table 1; Table 2; Figure 3; Figure 4). Compound **1** (the most active compound identified, according to pIC_50_ and ΔA) behaved as a non-competitive inhibitor and **3** as a mixed inhibitor (with the second-best value of pIC_50_ and ΔA). In both cases, when the concentration of the compounds increased, the slope and intersection with the ordinate axis changed, but the intersection with the abscissae did not. In addition, the K_IC_ and K_IU_ values changed for compound **3** (Spector and Cleland, 1981; Copeland, 2000). In both cases, the inhibition mechanism discovered was relevant because they interacted with a distinct area from the active site, guaranteeing a selective interaction with PTP1B.

**TABLE 2:** PTP1B nonlinear fit and inhibitory activity parameters of **1**, **3**, **and** **6**.

Compound	Reduce χ^2 [a]^	V_MAX_^[b]^ (mM min^−1^)	K_I_^[c]^ (mM)
**1**	0.6	44.1	KIcu = 0.009
**3**	1.4	56.1	K_IU_ = 0.11, K_IC_ = 0.04
**6 *E*/*Z***	0.08	24.7	K_IC_ = 0.031, K_IC2_ = 0.020

[a] Parameter of nonlinear fit. [b] Maximum velocity. [c] Inhibition constant according to the type.

For *E*/*Z* vermelhotin (**6**), the kinetic analysis data fit for a competitive parabolic inhibition; the statistical analysis of this fit predicted that two molecules of **6** bound to the active site (Table 2), which is possible due to the small molecular size. Figure 4A presents the mechanism of inhibition for **6**, implying mutually exclusive binding of the inhibitor and the substrate, allowing for two molecules of the inhibitor to bind to the enzyme. In the double-reciprocal plot, the V_MAX_ value did not show any change, but the K_M_ value changed significantly (Figure 4B). The modification of the structure of *E*/*Z* vermelhotin could improve its primary site of interaction.

The classic Michaelis–Menten plots were also convenient for defining the mode of inhibition of compounds **1**, **3,** and **6** and have been included in Supplementary Figure S3.

### 3.3 SAS map

**FIGURE 5:**
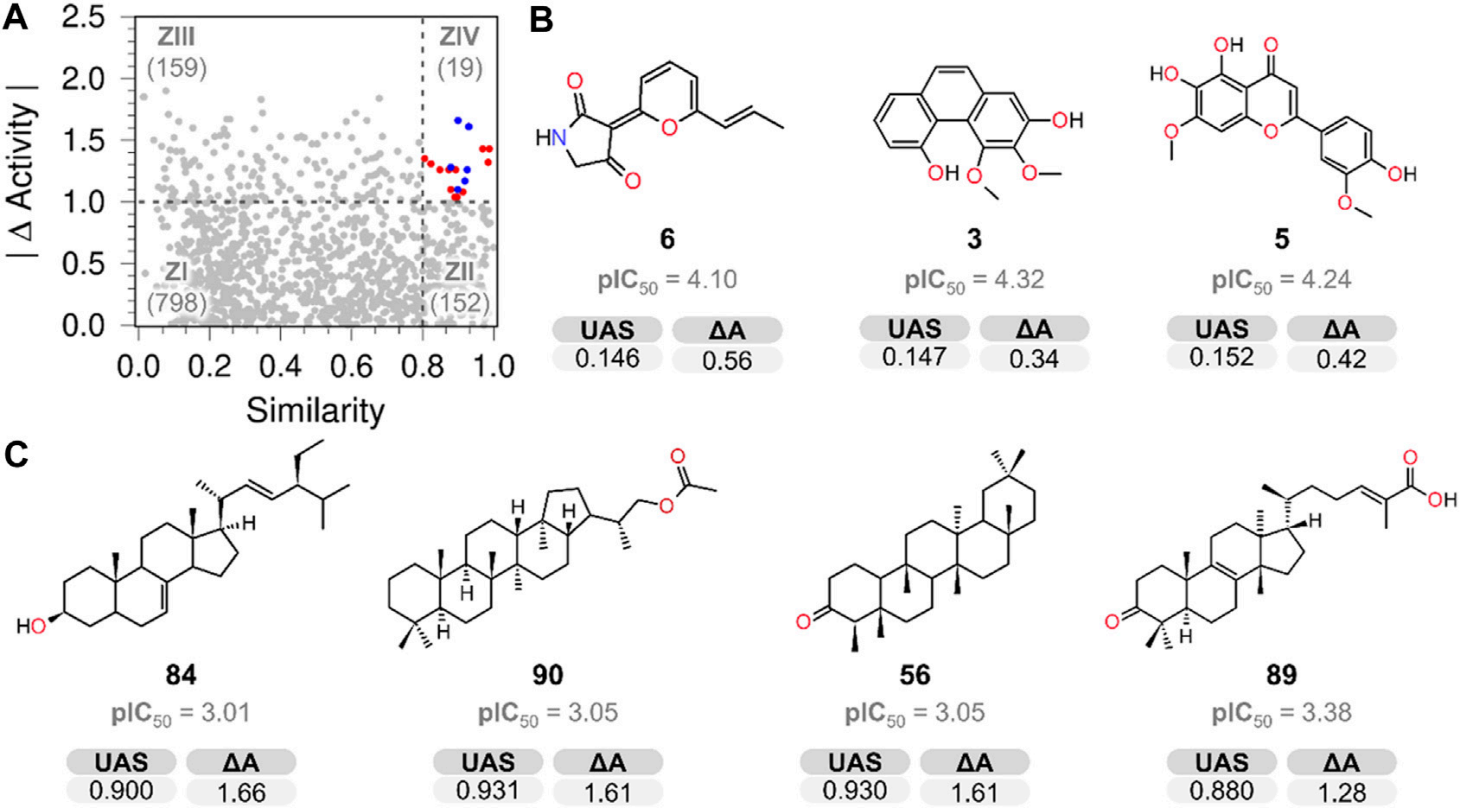
**(A)** SAS map shows the number of compound pairs in each zone. **(B)** Chemical structures identified as key scaffold hops (ZI). **(C)** Activity cliff compounds located in ZIV.

A SAS map (Medina-Franco, 2012) was created to identify scaffold hopes (compounds with low structural similarity but low activity difference) by plotting the absolute value of the PTP1B pIC_50_ difference (|∆Activity|) against ursolic acid structural similarity (Tanimoto similarity indexes) (Figure 1 and Figure 5) employing atom-type fingerprints. The resulting map was divided into four zones (ZI–ZIV), comprising the scaffold hopping region (ZI), smooth or continuous SAR (ZII), a non-descript region (ZIII), and activity cliffs (ZIV) (Naveja et al., 2018).

The analysis of the ZI region (818 pairs) revealed that NPs **3**, **5**, **6** (Figure 5B), **7**, **8**, **9**, **11**, **34**, **82**, and **99** (Supplementary Figure S15) possessed low structural similarity to the reference compound (UA) and good PTP1B inhibitory potency, therefore they are considered scaffold hops. In this group, it is not possible to make conclusions regarding structural activity relationships due to the diversity of structures. The structural simplicity of compounds **3** and **6** makes them hit compounds, likely to be optimized and used for developing potential drugs with novel scaffolds.

The SAS map allows the identification of canophyllol (**1**), also a pentacyclic triterpenoid, as the only true analog of UA (located in ZII), justified as the smallest ΔA obtained (0.2); the results suggest that the pentacyclic structure and oxygenated functionalities at C-3 and C-28 make similar compounds more active. This information is consistent with other UA derivatives possessing alcohol and carboxyl functionality at C-3 and C-28, respectively (Guzmán-Ávila et al., 2018; Khwaza et al., 2020).

**FIGURE 6:**
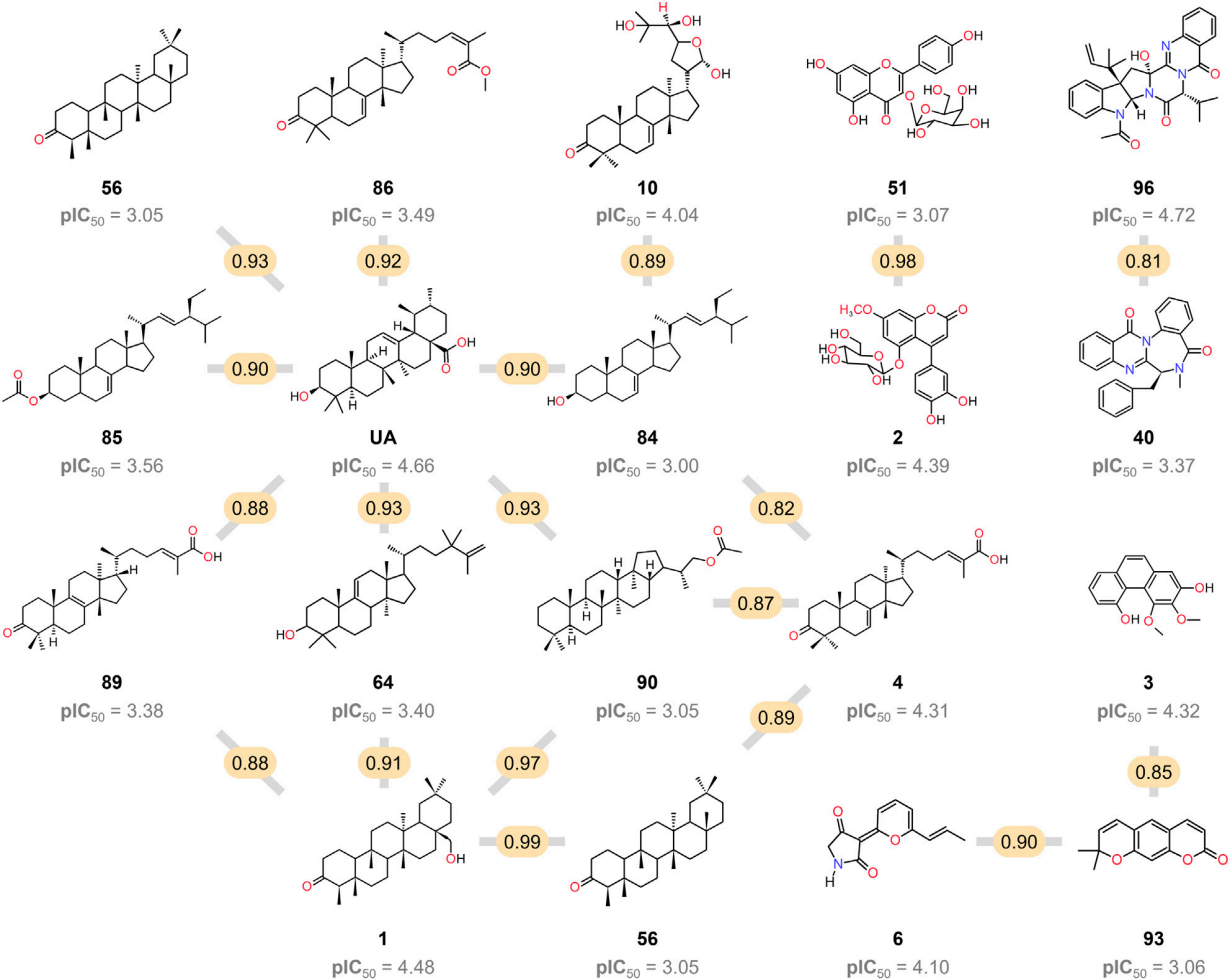
Chemical structure of the 19 pairs of compounds identified as activity cliffs in the SAS map. The compared pairs are represented by a gray line, and the molecular similarity value is highlighted in yellow.

This research also examined several triterpenoids that included **4**, **10**, **56**, **64**, **85**, **86**, and **89**, with a lower activity level than UA. None of them had the same pentacyclic core as UA and lacked the alcohol or carboxylic functionality at C-3 and C-28, respectively, suggesting that these structural features are important for activity (Figure 6).

### 3.4 Molecular docking analysis of hit compounds

**FIGURE 7:**
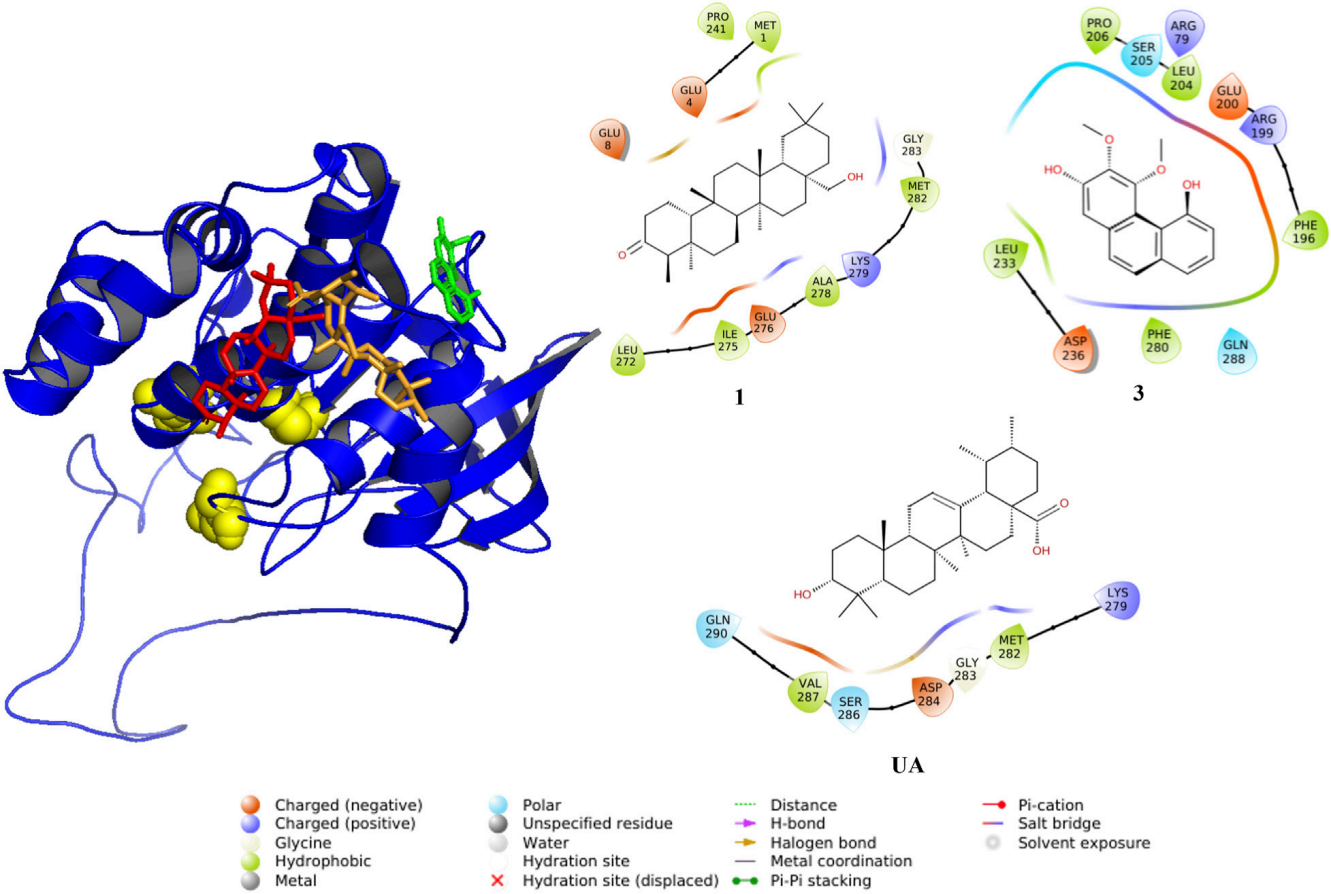
Predicted interactions between ligands and PTP1B (AlphaFold code: Q9PT91). The protein is displayed in blue (cartoon), and active sites are shown as yellow spheres and compounds as sticks: red (**1**), green (**3**), and UA (orange).

**FIGURE 8:**
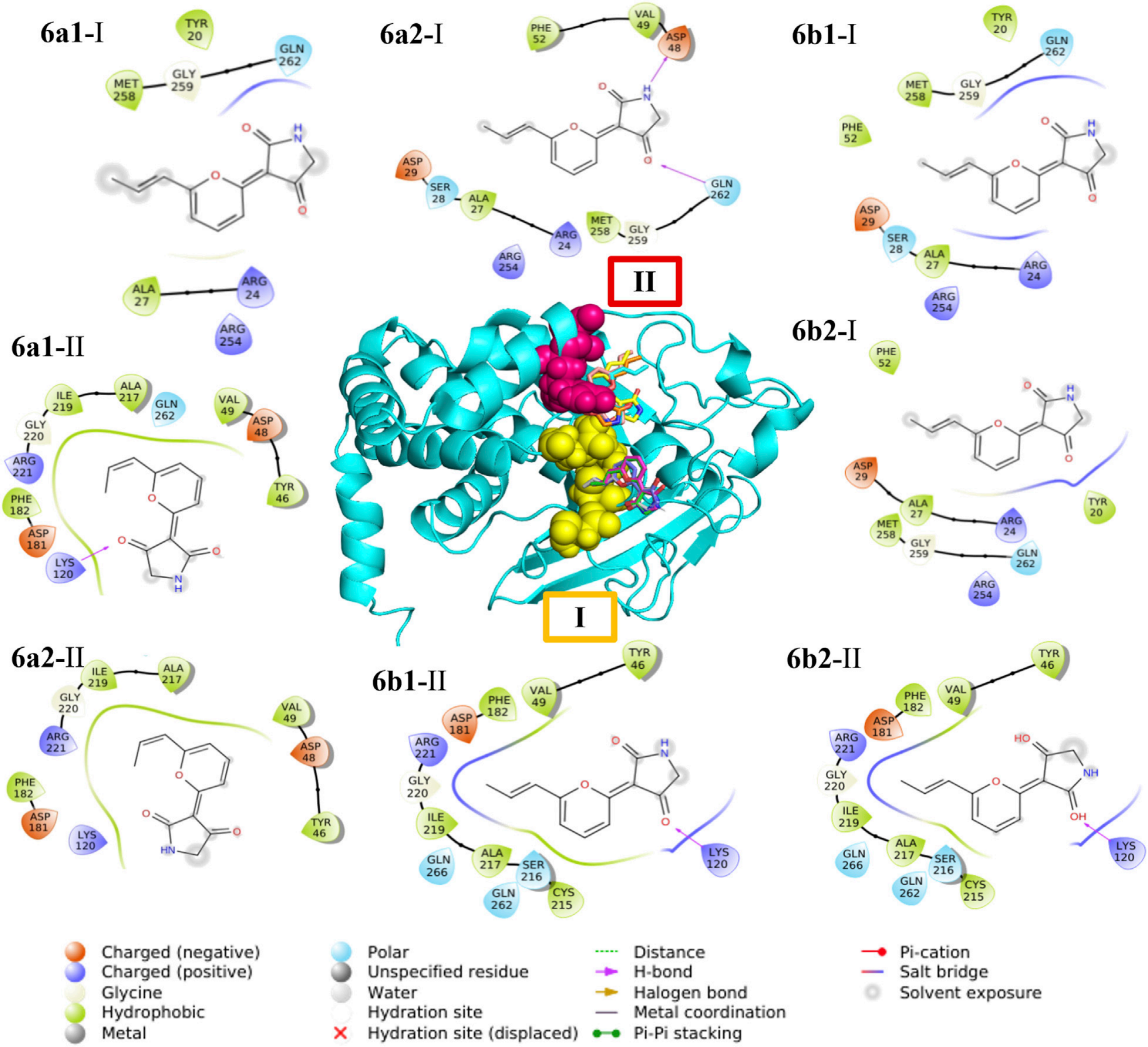
Predicted interactions between PTP1B (AlphaFold code: Q9PT91) and the four more stable conformational isomer complexes of *E*/*Z* vermelhotin (**6**). In the center, the protein is displayed in cyan (cartoon); pocket I residues are shown as pink spheres and pocket II residues are represented as yellow spheres. The conformational entities in both pockets are shown as sticks: green (**6a1**), pink (**6a2**), gray (**6b1**), slate (**6b2**), cyan (**6a1**), yellow (**6a2**), salmon (**6b1**), and orange (**6b2**).

To predict the binding modes between compounds **1–11** and PTP1B, a docking analysis was performed using the AlphaFold enzyme (code: Q9PT91), which was first subjected to molecular dynamics simulation (MDS) (Supplementary Figure S5A,B). After 100 ns, the region between amino acids 300 and 400 of PTP1B (Supplementary Figure S6A), which is an intrinsically unstructured site, was more stable (Supplementary Figure S6B). The robustness of the structural model was supported by Ramachandran plots and quality scores, before and after the MDS. Therefore, the model shown in Figure F6B was used for docking studies. This section shows only the results of **1**, **3**, and **6** (Table 3; Figures 7, 8) to correlate the findings with those of the kinetic analysis. Compounds **1** and **3** bind in a pocket formed by the 3α, 6α, and 7α helices near the *C*-terminal region of PTP1B, as previously reported for UA (Liu et al., 2022); this outcome is in agreement with the non-competitive mechanism found in the kinetic studies. Compounds **1** and **3** interacted with different amino acids, but the nature of the interactions was predominantly hydrophobic. Compound **1** had lower energy binding.

**TABLE 3:** Results of molecular docking between PTP1B and compounds **1**, **3**, **and** **6**.

Compound	ΔG_T_^[a]^ (Kcal mol^−1^)	Interacting residues
**1**	−6.71	Met1, Glu8, Glu4, Pro241, Gly283, Met282, Lys279, Ala278, Glu276, Ile275, Leu 272
**3**	−5.73	Pro206, Ser205, Leu204, Arg79, Glu200, Arg199, Phe196, Gln288, Phe280, Asp236, Leu233
**6a1 *E***	I^[b]^ = −5.67	I^[b]^: Ala27, Arg24, Arg254, Gln262, Gly259, Met258, Tyr20
	II^[c]^ = −6.14	II^[c]^: Ala217, Arg221, Asp181, Asp48, Gln262, Gly220, Ile219, Lys120, Phe182, Tyr46, Val49
**6a2 *E***	I: −5.86	I: Ala27, Arg24, Arg254, Asp29, Asp48, Gln262, Gly259, Met258, Phe52, Ser28, Val49
	II: −6.02	II: Ala217, Asp181, Asp48, Gln262, Gly220, Ile219, Lys120, Phe182, Tyr46, Val49
**6b1 *Z***	I: −5.95	I: Ala27, Arg24, Arg254, Asp29, Gln262, Gly259, Met258, Phe52, Ser28, Tyr20
	II: −6.70	II: Ala217, Arg221, Asp181, Cys215, Gln262, Gln266, Gly220, Ile219, Lys120, Phe182, Ser216, Tyr46, Val49
**6b2 *Z***	I: −5.78	I: Ala27, Arg24, Arg254, Asp29, Gln262, Gly259, Met258, Phe52, Tyr20
	II: −6.64	II: Ala217, Arg221, Asp181, Cys215, Gln262, Gln266, Gly220, Ile219, Lys120, Phe182, Ser216, Tyr46, Val49
**UA**	−6.73	Gln290, Val287, Ser286, Asp284, Gly283, Met282, Lys279

^[a]^Theoretical binding energy.

^[b]^Pocket I where **6** interacts.

^[c]^Pocket II where **6** interacts.

Since compound **6** undergoes interconversion between the *E* (**6a1**, **6b1**) and *Z* (**6a2**, **6b2**) isomers (Supplementary Figure S4), forming an equilibrium *E*/*Z* mixture with a ratio of 6.4:3.6 (Leyte-Lugo et al., 2012), the docking analysis was undertaken with the four more stable conformational isomers. For all conformers, **6a1**, **6a2**, **6b1**, and **6b2**, the low-energy docking events fell in two contiguous relatively separate pockets designated as I and II (Figure 8 and Supplementary Figure S7–S10), where simultaneous binding of two molecules might occur. Conformers **6b1** and **6b2** predominantly bind to pocket II, which includes active site residues supporting the parabolic competitive inhibition kinetic mechanism found for NP **6**. Pocket I, where **6a1** and **6a2** might attach, contains secondary residues different from the active site (Liu et al., 2022). The lowest energy pose was in pocket II (active site), followed by the pose at site I, which may be considered an allosteric spot (Table 3; Figure 8, Supplementary Figure S7–S10). This outcome is relevant because the bidentate PTP1B inhibitors bind simultaneously to the catalytic and allosteric sites, conferring higher specificity and potency to these inhibitors (Chen et al., 2018; Akyol and Kilic, 2021).

The docking studies for compounds **2**, **4**, **5**, and **7–11** are given in Supplementary Figure S11, S12 and Supplementary Table S3, S4. All compounds bound to an allosteric area in the enzyme; **2**, **4**, **5**, and **7–11** presented ΔG between −7.2 and −5.6 kcal mol^−1^, and their interactions were hydrophobic; metabolites **4**, **7**, **9**, and **10** attached in the region formed by helices 3α, 6α, and 7α, the same as UA. In the case of **2**, **8**, and **11**, they were positioned near the 3α and 9α helices and two β strands. Finally, among all hits, flavonoid **8** showed the best binding energy to the protein.

### 3.5 Medicinal chemistry and ADMET predictions of most active NPs

To estimate the safety and efficacy of the 11 active NPs, their physicochemical, pharmacokinetic, and pharmacodynamic profiles were analyzed to predict their potential use in drug development (Supplementary Figure S13, Supplementary Table S5–S11). All the results predicted were only taken as an indicator of passive absorption properties because NPs or NP-derived drugs have more than two violations of the Lipinski’s rule and their physicochemical, pharmacokinetic, and pharmacodynamic profiles are different from those of the approved drugs (Boufridi and Quinn, 2018).

**FIGURE 9:**
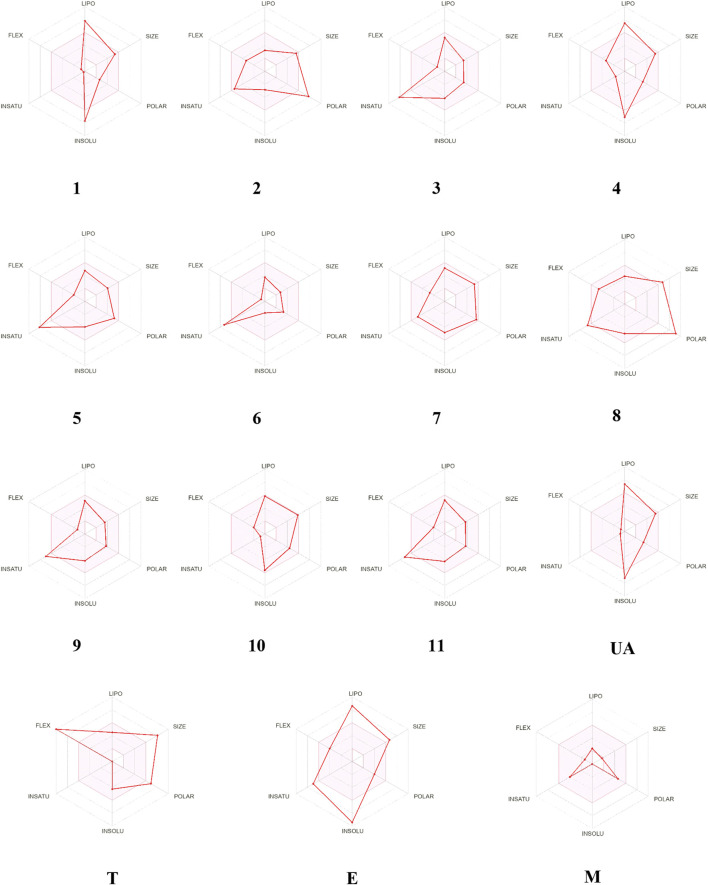
Visual representation of the optimal range for each property presented in axes. Trodusquemine (T), ertiprotafib (E), and metformin (M).

The bioavailability radar (Figure 9) predicted that compounds **1–11** possess good physicochemical properties. However, the degree of unsaturation and solubility values were out of the optimal range in almost all the molecules but **7**. Regarding the medicinal chemistry rules, only **3**, **6**, **7**, **9**, and **10** fulfilled the Lipinski and Golden Triangle rules (Supplementary Table S6); compounds **2**, **5**, and **8** are pan-assay interference substances (PAINS) due to the catechol fragment motifs; in consequence, they could show false positive results in biological assays. Finally, all compounds, excluding **1**, showed one or two BRENK alerts, so they possess unwanted fragments (Supplementary Table S6).

**FIGURE 10:**
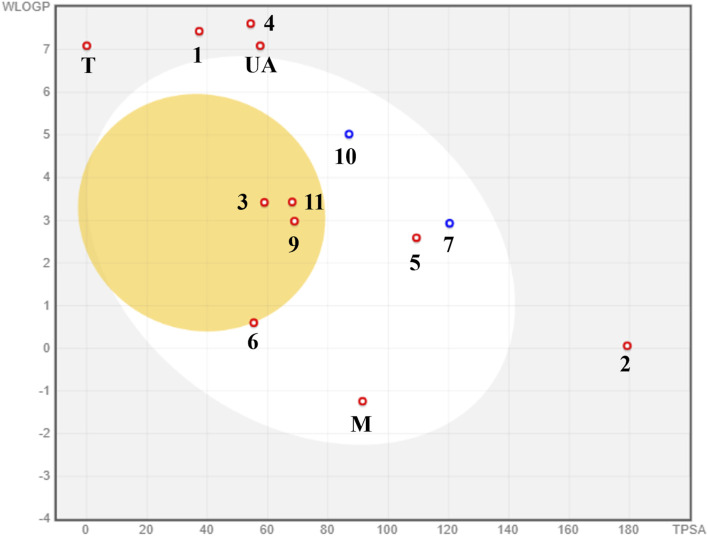
BOILED-Egg model for the prediction of passive absorption in GI and BBB using SwissADME.

The predictions related to passive permeability in the GI tract and BBB, as well as interaction with the glycoprotein P (PGP), are presented in the BOILED-Egg (Figure 10); the visual analysis of the BOILED-Egg graphic shows that **3**, **9**, and **11** could passively cross the BBB, therefore they could be neurotoxic. It is possible that **5–7** and **10** may experience passive gastrointestinal absorption because they share similar physicochemical properties with oral medications, such as M. T and E fell outside the graphic, precluding any prediction regarding their absorption type. Finally, only the prenylated xanthone **7** and triterpenoid **10** could be a substrate of PGP, indicating low possibility for unsafe drug–drug interaction and problems with their excretion.

In addition, the medium lethal concentration doses (LD_50_s) were estimated using TEST v.5.1; all calculations are given in Supplementary Table S10, S11. The toxicity conclusion must be interpreted cautiously; no compound is free of toxic effects. According to these results, compounds **1**, **6**, and **10** were the NPs with fewer toxicological alerts. The remaining compounds presented one or two alerts. The most relevant toxicological alerts were for compounds **9** (high mutagenic and teratogenic effects), **5,** and **7** (high mutagenicity), and compound **2** (high teratogenic action). Regarding the LD_50_s, compounds **1**, **3**, and **6** showed LD_50_s ≥ 1,000 mg/kg.

**FIGURE 11:**
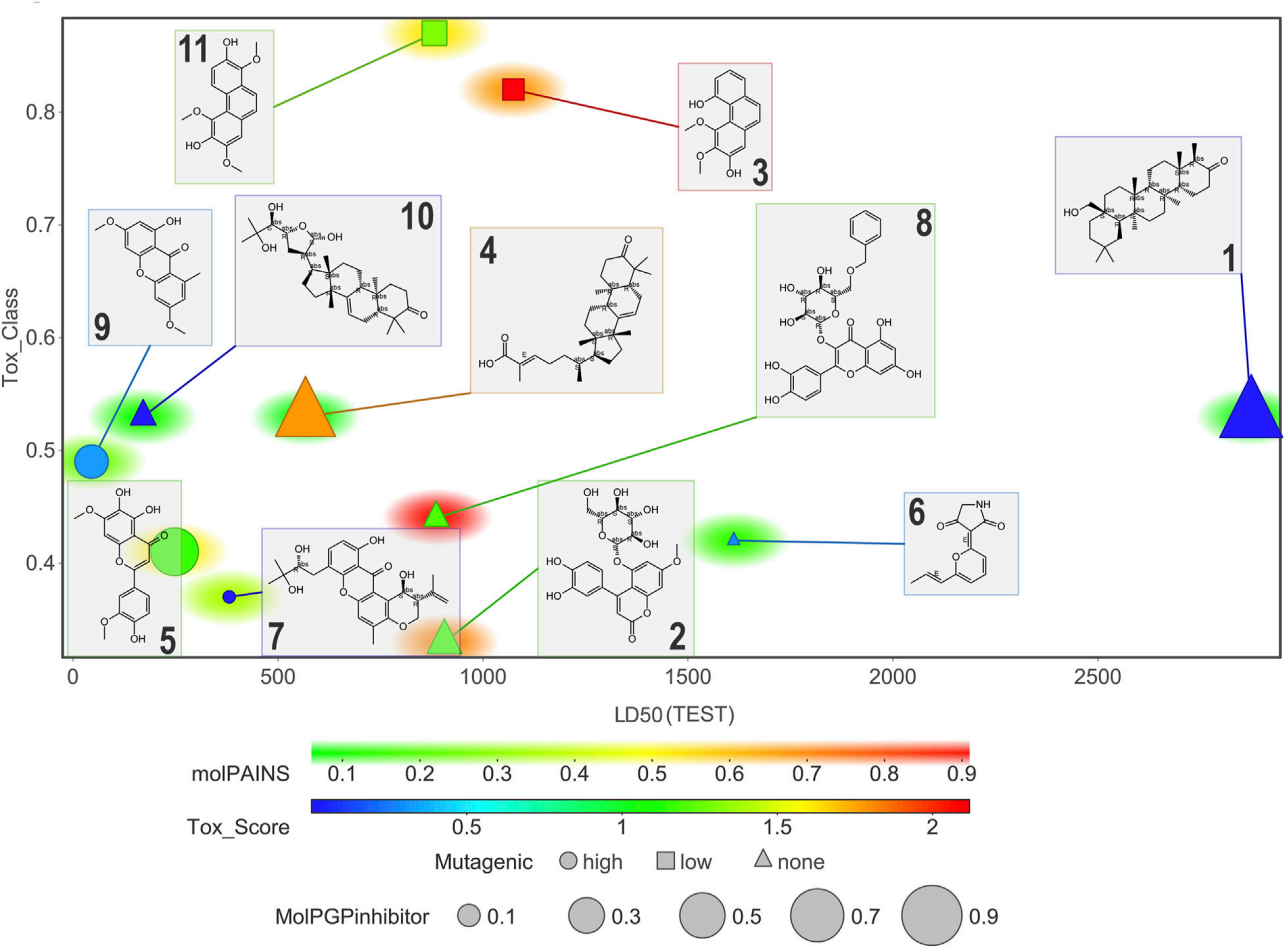
Graphic representation of some of the estimated toxicological properties for compounds **1–11**. Correlation plot of compounds according to their estimated LD_50_ and toxicity class. Marker size indicates the probability for a compound to be a PGP inhibitor (the larger, the more probable). Marker color refers to the toxicity score (chemical alerts found in the structure; values > 1 indicate unfavorable substructures). Marker shape indicates high (circle), low (square), or negligible (triangle) mutagenicity. Marker background color correlates with the probability of being a PAINS.

**FIGURE 12:**
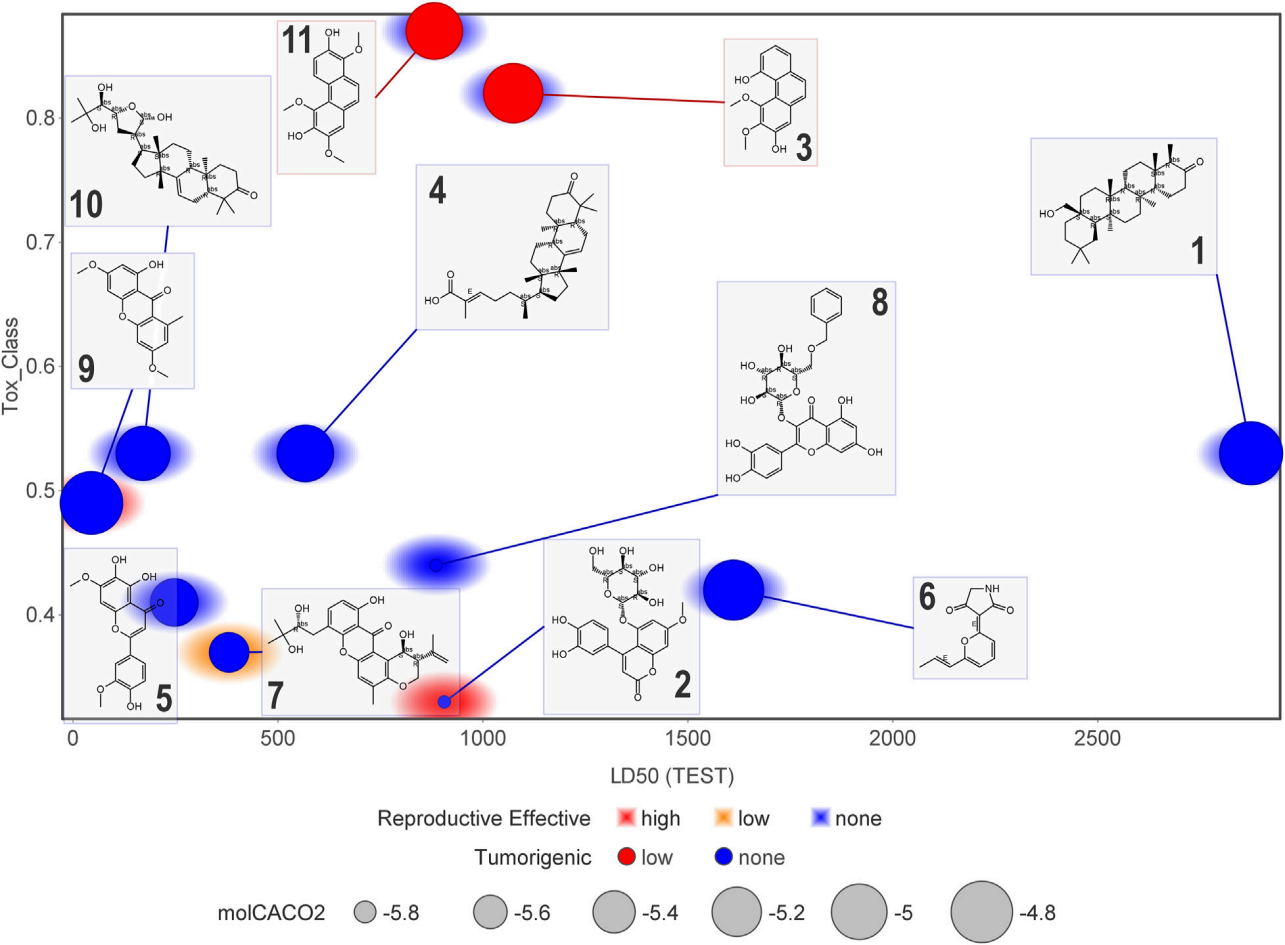
Graphical representation of some of the estimated toxicological properties for compounds **1–11**. Correlation plot of compounds according to their estimated LD_50_ and toxicity class. Marker size indicates the Caco-2 cell permeability related to *in vivo* absorption. Marker color refers to low or non-tumorigenic effect. The marker background color indicates teratogenicity (reproductive effect).

Figure 11 and Figure 12 display six toxicological properties on a graph to better understand potentially toxic molecules. On average, NPs **1** and **6** showed the lowest number of alerts (three and six, respectively) (Supplementary Table S11). Experimental Lorke toxicity evaluation of compound **6** revealed no acute toxic effect on mice; the experimental LC_50_ value was estimated to be higher than 5,000 mg/kg, in agreement with the predicted results.

## 4 Conclusion

From a small and diverse NP library selected mostly on the basis of an ethnopharmacological criterion, 11 hit compounds (**1–11**) were identified, seven of which were scaffold hops (**3**, **5**, **6**, **7**, **8**, **9**, and **11**). NPs **1–5**, **8**, **10**, and **11** are used in different traditional preparations for treating diabetes or its complications. Therefore, this work provides information supporting the rational use of some Mexican medicinal plants employed for treating diabetes.

The 47 active compounds detected after the preliminary screen are chemically diverse, covering most of the explored space of Mexican NPs. The Tanimoto similarity indexes and the absolute value of the PTP1B inhibitory activity differences of all pairs of compounds allowed identifying 10 hit molecules (**3**, **5**, **6**, **7**, **8**, **9**, **11**, **34**, **82**, and **99**) with low structural similarity to the reference compound (UA) but good PTP1B inhibitory potency (scaffold hops). The structural simplicity of **3** and **6** pinpoint them as hits for developing potential drugs with novel scaffolds.

After thoroughly analyzing the medicinal chemistry and physicochemical, pharmacokinetic, and toxicological properties of various NPs, **1**, **6**, and **10** seem to be the most promising hit compounds as PTP1B inhibitors. According to the Lorke analysis, compound **6** is not toxic to mice, with an LC_50_ value higher than 5,000 mg/kg. The three compounds warrant drug optimization analysis to develop new leads for drug development.

## Data Availability

The original contributions presented in the study are included in the article/Supplementary Material; further inquiries can be directed to the corresponding authors.

## References

[B1] ADA (2022). 9. Pharmacologic approaches to glycemic treatment: standards of medical Care in diabetes—2022. Diabetes Care 45, S125–S143. 10.2337/dc22-S009 34964831

[B2] Akyol K. Kilic D. (2021). Discovery of novel and selective inhibitors targeting protein tyrosine phosphatase 1B (PTP1B): virtual screening and molecular dynamic simulation. Comput. Biol. Med. 139, 104959. 10.1016/j.compbiomed.2021.104959 34735946

[B3] Bajusz D. Rácz A. Héberger K. (2015). Why is Tanimoto index an appropriate choice for fingerprint-based similarity calculations? J. Cheminformatics 7 (1), 20–13. 10.1186/s13321-015-0069-3 PMC4456712 26052348

[B5] Boufridi A. Quinn R. J. (2018). Harnessing the properties of natural products. Annu. Rev. Pharmacol. Toxicol. 58, 451–470. 10.1146/annurev-pharmtox-010716-105029 28968192

[B6] Calzada F. Navarrete A. del Rio F. Delgado G. (1991). Long-chain phenols from the bark of Amphypterygium adstringens. J. Ethnopharmacol. 34 (2–3), 147–154. 10.1016/0378-8741(91)90032-9 1795518

[B7] Cao D.-S. Xiao N. Xu Q. S. Chen A. F. (2015). Rcpi: R/Bioconductor package to generate various descriptors of proteins, compounds and their interactions. Bioinformatics 31 (2), 279–281. 10.1093/bioinformatics/btu624 25246429

[B8] Chen X. Gan Q. Feng C. Liu X. Zhang Q. (2018). Virtual screening of novel and selective inhibitors of protein tyrosine phosphatase 1B over T-cell protein tyrosine phosphatase using a bidentate inhibition strategy. J. Chem. Inf. Model. 58 (4), 837–847. 10.1021/acs.jcim.8b00040 29608303

[B9] Copeland R. A. (2000). Enzymes. A practical introduction to structure, mechanism, and data analysis. Second. New York: Wiley-CVH. 10.1006/abio.2001.5023

[B10] Daina A. Michielin O. Zoete V. (2017). SwissADME: a free web tool to evaluate pharmacokinetics, drug-likeness and medicinal chemistry friendliness of small molecules. Sci. Rep. 7, 42717. 10.1038/srep42717 28256516 PMC5335600

[B11] David L. Thakkar A. Mercado R. Engkvist O. (2020). Molecular representations in AI-driven drug discovery: a review and practical guide. J. Cheminformatics 12 (1), 56–22. 10.1186/s13321-020-00460-5 PMC7495975 33431035

[B12] DeLano W. L. (2004). Use of PYMOL as a communications tool for molecular science. Abstr. Pap. Am. Chem. Soc. 228, U313–U314.

[B13] Díaz-Rojas M. Raja H. González-Andrade M. Rivera-Chávez J. Rangel-Grimaldo M. Rivero-Cruz I. (2021). Protein tyrosine phosphatase 1B inhibitors from the fungus Malbranchea albolutea. Phytochemistry 184, 112664. 10.1016/j.phytochem.2021.112664 33524855

[B14] EberhardtSantos-MartinsTillack J. D. A. F. Forli S. (2021). AutoDock Vina 1.2.0: new docking methods, expanded force field, and Python bindings. J. Chem. Inf. Model. 61 (8), 3891–3898. 10.1021/acs.jcim.1c00203 34278794 PMC10683950

[B15] Estrada S. Toscano R. A. Mata R. (1999). New phenanthrene derivatives from Maxillaria densa. J. Nat. Prod. 62 (8), 1175–1178. 10.1021/np990061e 10479332

[B16] Figueroa M. González M. d. C. Rodríguez-Sotres R. Sosa-Peinado A. González-Andrade M. Cerda-García-Rojas C. M. (2009). Calmodulin inhibitors from the fungus Emericella sp. Bioorg. Med. Chem. 17 (6), 2167–2174. 10.1016/j.bmc.2008.10.079 19013822

[B17] Flores-Bocanegra L. Pérez-Vásquez A. Torres-Piedra M. Bye R. Linares E. Mata R. (2015). α-Glucosidase inhibitors from vauquelinia corymbosa. Molecules 20 (8), 15330–15342. 10.3390/molecules200815330 26307962 PMC6332183

[B18] Guerrero-Analco J. A. Hersch-Martínez P. Pedraza-Chaverri J. Navarrete A. Mata R. (2005). Antihyperglycemic effect of constituents from Hintonia standleyana in streptozotocin-induced diabetic rats. Planta Medica 71 (12), 1099–1105. 10.1055/s-2005-873137 16395644

[B19] Guha R. (2007). Chemical informatics functionality in R. J. Stat. Softw. 18 (5). 10.18637/jss.v018.i05

[B20] Guzmán-Ávila R. Flores-Morales V. Paoli P. Camici G. Ramírez-Espinosa J. J. Cerón-Romero L. (2018). Ursolic acid derivatives as potential antidiabetic agents: *in vitro, in vivo*, and *in silico* studies. Drug Dev. Res. 79 (2), 70–80. 10.1002/ddr.21422 29380400

[B21] Hanwell M. D. Curtis D. E. Lonie D. C. Vandermeersch T. Zurek E. Hutchison G. R. (2012). Avogadro: an advanced semantic chemical editor, visualization, and analysis platform. J. ofCheminformatics 4 (17), 17. 10.1186/1758-2946-4-17 PMC3542060 22889332

[B22] Harvey A. L. Clark R. L. Mackay S. P. Johnston B. F. (2010). Current strategies for drug discovery through natural products. Expert Opin. Drug Discov. 5 (6), 559–568. 10.1517/17460441.2010.488263 22823167

[B23] HumphreyDalke W. A. Schulten K. (1996). VMD: visual molecular dynamics. J. Mol. Graph. 14 (1), 33–38. 10.1016/0263-7855(96)00018-5 8744570

[B24] IDF (2021). “IDF diabetes atlas,” in Diabetes research and clinical practice. 10th edn. 10.1016/j.diabres.2013.10.013 26119773

[B25] Jimenez A. Villarreal C. Toscano R. A. Cook M. Arnason J. T. Bye R. (1998). Limonoids from swietenia humilis and guarea grandiflora (Meliaceae)Taken in part from the PhD and MS theses of C. Villarreal and M. A. Jiménez, respectively. Phytochemistry 49 (7), 1981–1988. 10.1016/s0031-9422(98)00364-1

[B26] Jiménez-Arreola B. S. Aguilar-Ramírez E. Cano-Sánchez P. Morales-Jiménez J. González-Andrade M. Medina-Franco J. L. (2020). Dimeric phenalenones from Talaromyces sp. (IQ-313) inhibit hPTP1B1-400: insights into mechanistic kinetics from *in vitro* and *in silico* studies. Bioorg. Chem. 101, 103893. 10.1016/j.bioorg.2020.103893 32492551

[B27] Kalliokoski T. Kramer C. Vulpetti A. Gedeck P. (2013). Comparability of mixed IC₅₀ data - a statistical analysis. PLoS ONE 8 (4), e61007. 10.1371/journal.pone.0061007 23613770 PMC3628986

[B28] Kanwal A. Kanwar N. Bharati S. Srivastava P. Singh S. P. Amar S. (2022). Exploring new drug targets for type 2 diabetes: success, challenges and opportunities. Biomedicines 10 (2), 331. 10.3390/biomedicines10020331 35203540 PMC8869656

[B29] Kazakova O. Giniyatullina G. Babkov D. Wimmer Z. (2022). From marine metabolites to the drugs of the future: squalamine, trodusquemine, their steroid and triterpene analogues. Int. J. Mol. Sci. 23 (3), 1075. 10.3390/ijms23031075 35162998 PMC8834734

[B30] Khwaza V. Oyedeji O. O. Aderibigbe B. A. (2020). Ursolic acid-based derivatives as potential anti-cancer agents: an update. Int. J. Mol. Sci. 21 (16), 5920–5927. 10.3390/ijms21165920 32824664 PMC7460570

[B31] Kumar G. S. Page R. Peti W. (2020). The mode of action of the Protein tyrosine phosphatase 1B inhibitor Ertiprotafib. PLOS ONES 15 (10), e0240044. 10.1371/journal.pone.0240044 PMC7531832 33007022

[B32] Lahlou M. (2007). Screening of natural products for drug discovery. Expert Opin. Drug Discov. 2 (5), 697–705. 10.1517/17460441.2.5.697 23488959

[B33] Landrum G. (2022). RDKit: open-source cheminformatics.

[B34] Lantz K. A. Hart S. G. E. Planey S. L. Roitman M. F. Ruiz-White I. A. Wolfe H. R. (2010). Inhibition of PTP1B by trodusquemine (MSI-1436) causes fat-specific weight loss in diet-induced obese mice. Obesity 18 (8), 1516–1523. 10.1038/oby.2009.444 20075852

[B35] Leyte-Lugo M. González-Andrade M. González M. d. C. Glenn A. E. Cerda-García-Rojas C. M. Mata R. (2012). (+)-Ascosalitoxin and vermelhotin, a calmodulin inhibitor, from an endophytic fungus isolated from Hintonia latiflora. J. Nat. Prod. 75 (9), 1571–1577. 10.1021/np300327y 22924467

[B36] Liu R. Mathieu C. Berthelet J. Zhang W. Dupret J. M. Rodrigues Lima F. (2022). Human protein tyrosine phosphatase 1B (PTP1B): from structure to clinical inhibitor perspectives. Int. J. Mol. Sci. 23 (13), 7027. 10.3390/ijms23137027 35806030 PMC9266911

[B37] Lorke D. (1983). A new approach to practical acute toxicity testing. Archives Toxicol. 54 (4), 275–287. 10.1007/BF01234480 6667118

[B38] Medina-Franco J. L. (2012). Scanning structure-activity relationships with structure-activity similarity and related maps: from consensus activity cliffs to selectivity switches. J. Chem. Inf. Model. 52 (10), 2485–2493. 10.1021/ci300362x 22989212

[B39] Morales-Sánchez V. Rivero-Cruz I. Laguna-Hernández G. Salazar-Chávez G. Mata R. (2014). Chemical composition, potential toxicity, and quality control procedures of the crude drug of Cyrtopodium macrobulbon. J. Ethnopharmacol. 154 (3), 790–797. 10.1016/j.jep.2014.05.006 24818583

[B40] Na B. Nguyen P. H. Zhao B. T. Vo Q. H. Min B. S. Woo M. H. (2016). Protein tyrosine phosphatase 1B (PTP1B) inhibitory activity and glucosidase inhibitory activity of compounds isolated from Agrimonia pilosa. Pharm. Biol. 54 (3), 474–480. 10.3109/13880209.2015.1048372 26084800

[B41] Naveja J. J. Oviedo-Osornio C. I. Medina-Franco J. L. (2018). Computational methods for epigenetic drug discovery: a focus on activity landscape modeling. Adv. Protein Chem. Struct. Biol. 113, 65–83. 10.1016/bs.apcsb.2018.01.001 30149906

[B42] NCBI (2009). PubChem subgraph fingerprint. Available at: https://ftp.ncbi.nlm.nih.gov/pubchem/specifications/pubchem_fingerprints.pdf.

[B43] O’Boyle N. M. Banck M. James C. A. Morley C. Vandermeersch T. Hutchison G. R. (2011). Open Babel: an open chemical toolbox. J. Cheminformatics 3 (1), 33. 10.1186/1758-2946-3-33 PMC3198950 21982300

[B4] Pedregosa F. Varoquaux G. Gramfort A. Michel V. Thirion B. Grisel O. (2011). Scikit-learn: machine learning in Python. J. Mach. Learn. Res. 12, 2825–2830. 10.48550/arXiv.1201.0490

[B44] Pilón-Jiménez B. A. Saldívar-González F. I. Díaz-Eufracio B. I. Medina-Franco J. L. (2019). BIOFACQUIM: a Mexican compound database of natural products. Biomolecules 9 (1), 31. 10.3390/biom9010031 30658522 PMC6358837

[B45] Quy P. T. Van Hue N. Bui T. Q. Triet N. T. Van Chen T. Van Long N. (2022). Inhibitory, biocompatible, and pharmacological potentiality of dammarenolic-acid derivatives towards α-glucosidase (3W37) and tyrosine phosphatase 1B (PTP1B). Vietnam J. Chem. 60 (2), 223–237. 10.1002/vjch.202100189

[B46] R Core Team (2020). R: a language and environment for statistical computing. Vienna, Austria: R Foundation for Statistical Computing.

[B47] Rangel-Grimaldo M. Macías-Rubalcava M. L. González-Andrade M. Raja H. Figueroa M. Mata R. (2020). α-Glucosidase and protein tyrosine phosphatase 1B inhibitors from malbranchea circinata. J. Nat. Prod. 83 (3), 675–683. 10.1021/acs.jnatprod.9b01108 31898904

[B48] Rivero-Cruz I. Acevedo L. Guerrero J. A. Martínez S. Bye R. Pereda-Miranda R. (2010). Antimycobacterial agents from selected Mexican medicinal plants. J. Pharm. Pharmacol. 57 (9), 1117–1126. 10.1211/jpp.57.9.0007 16105233

[B49] Roe D. R. Cheatham T. E. (2013). PTRAJ and CPPTRAJ: software for processing and analysis of molecular dynamics trajectory data. J. Chem. Theory Comput. 9 (7), 3084–3095. 10.1021/ct400341p 26583988

[B50] Rojas I. S. Lotina-Hennsen B. Mata R. (2000). Effect of lichen metabolites on thylakoid electron transport and photophosphorylation in isolated spinach chloroplasts. J. Nat. Prod. 63 (10), 1396–1399. 10.1021/np0001326 11076561

[B51] Salinas-Arellano E. Pérez-Vásquez A. Rivero-Cruz I. Torres-Colin R. González-Andrade M. Rangel-Grimaldo M. (2020). Flavonoids and terpenoids with PTP-1B inhibitory properties from the infusion of salvia amarissima ortega. Molecules 25 (15), 3530. 10.3390/molecules25153530 32752292 PMC7435600

[B52] Sander T. Freyss J. von Korff M. Rufener C. (2015). DataWarrior: an open-source program for chemistry aware data visualization and analysis. J. Chem. Inf. Model. 55 (2), 460–473. 10.1021/ci500588j 25558886

[B53] Santiago Á. Guzmán-Ocampo D. C. Aguayo-Ortiz R. Dominguez L. (2021). Characterizing the chemical space of γ-secretase inhibitors and modulators. ACS Chem. Neurosci. 12 (15), 2765–2775. 10.1021/acschemneuro.1c00313 34291906

[B54] Sharma B. Xie L. Yang F. Wang W. Zhou Q. Xiang M. (2020). Recent advance on PTP1B inhibitors and their biomedical applications. Eur. J. Med. Chem. 199, 112376. 10.1016/j.ejmech.2020.112376 32416458

[B55] Singh S. Singh Grewal A. Grover R. Sharma N. Chopra B. Kumar Dhingra A. (2022). Recent updates on development of protein-tyrosine phosphatase 1B inhibitors for treatment of diabetes, obesity and related disorders. Bioorg. Chem. 121, 105626. 10.1016/j.bioorg.2022.105626 35255350

[B56] Song Y. H. Uddin Z. Jin Y. M. Li Z. Curtis-Long M. J. Kim K. D. (2017). Inhibition of protein tyrosine phosphatase (PTP1B) and α-glucosidase by geranylated flavonoids from Paulownia tomentosa. J. Enzyme Inhibition Med. Chem. 32 (1), 1195–1202. 10.1080/14756366.2017.1368502 PMC6010085 28933230

[B57] Spector T. Cleland W. W. (1981). Meanings of Ki for conventional and alternate-substrate inhibitors. Biochem. Pharmacol. 30 (1), 1–7. 10.1016/0006-2952(81)90277-X 7213409

[B58] Sud M. (2016). MayaChemTools: an open source package for computational drug discovery. J. Chem. Inf. Model. 56 (12), 2292–2297. 10.1021/acs.jcim.6b00505 28024397

[B59] Thakur A. Kumar A. Sharma V. Mehta V. (2022). PIC50: an open source tool for interconversion of PIC50 values and IC50 for efficient data representation and analysis. bioRxiv.

[B60] Tonks N. K. (2003). PTP1B: from the sidelines to the front lines!. FEBS Lett. 546 (1), 140–148. 10.1016/S0014-5793(03)00603-3 12829250

[B61] Williams T. Kelley C. Bersch C. Bröker H. B. Campbell J. Cunningham R. (2017). gnuplot 5.2. An interactive plotting program. Available at: http://www.gnuplot.info/docs_5,2 .

[B62] Xiong G. Wu Z. Yi J. Fu L. Yang Z. Hsieh C. (2021). ADMETlab 2.0: an integrated online platform for accurate and comprehensive predictions of ADMET properties. Nucleic Acids Res. 49 (W1), W5–W14. 10.1093/nar/gkab255 33893803 PMC8262709

