# Supplementary material for: Discovery of inhibitors of protein tyrosine phosphatase 1B contained in a natural products library from Mexican medicinal plants and fungi using a combination of enzymatic and in silico methods**
Source: Front Pharmacol. 2023 Oct 31;14:1281045. doi: 10.3389/fphar.2023.1281045 (PMC10644722; doi:10.3389/fphar.2023.1281045)
Supplement: Supplementary file 1 [file DataSheet1.PDF]

Supplementary Material

Tables and Figures

Discovery of inhibitors of protein tyrosine phosphatase 1B contained in a natural products library from Mexican medicinal plants and fungi using a combination of enzymatic and in silico methods**

Miriam Díaz-Rojas[1], Martín González-Andrade[2*], Rodrigo Aguayo-Ortiz[1], Rogelio Rodríguez-Sotres[1], Araceli Pérez-Vásquez[1], Abraham Madariaga-Mazón[3,4], Rachel Mata[1*]

[1]Laboratory of Pharmacognosy, College of Chemistry, Pharmacy Department, National Autonomous University of Mexico, Mexico City, Mexico

[2]Laboratory of Biosensor, College of Medicine, Biochemistry Department, National Autonomous University of Mexico, Mexico City, Mexico

[3]Laboratory of Biological and Computational Chemistry, Institute for Research in Applied Mathematics and Systems, Department of Physical chemistry, National Autonomous University of Mexico, Mexico City, Mexico

[4]Laboratory of Biological and Computational Chemistry, Institute of Chemistry, Department of Physical chemistry, National Autonomous University of Mexico, Merida, Mexico

List of supplementary material

Table S1. Results of inhibitory activity using an *in vitro* assay of 99 natural products. 3

Table S2. pIC_{50} data of most active compounds with a value in order of magnitude as the control (UA). 34

Table S3. Theoretical binding properties of compounds **2**, **4**, **7–11** with $PTP1B_{1-400}$. 37

Table S4. Interactions of compounds **2**, **4**, **7–11** with PTP1B-$_{1-400}$. 37

Table S5. Prediction of physicochemical properties of compounds **1–11**.................... 38

Table S6. Medicinal Chemistry properties of compounds **1–11**. 39

Table S7. Pharmacokinetic properties related to absorption and distribution of compounds **1–11**. 40

Table S8. Pharmacokinetic properties related to metabolism and excretion of compounds **1–11**. .. 41

Table S9. Toxicological properties of compounds **1–11** calculated using DataWarrior. 42

Table S10. Estimated toxicity profile for selected molecules (**1−11**).................... 43

Table S11. Summary of alerts ADMET for the most active compounds (**1–11**). 44

Figure S1. IC_{50} curves for the NP in Table 2, fit using Origin 8.0 software. 45

Figure S2. Tanimoto similarity cumulative distribution (TSD) plots. 48

Figure S3. Michaelis-Menten curves.................... 49

Figure S4. Equilibrium *E*/*Z* mixture.................... 50

Figure S5. RMSD and RMSF of 100ns of molecular dynamics simulation of the structural model obtained from Alphafold of the $PTP1B_{1-400}$. 51

Figure S6. Structural model, Ramachandran plot and quality score of $PTP1B_{1-400}$ before (**A**) and after 100 ns of molecular dynamics simulation (**B**). 52

Figure S7. Vermelhotin **6a1** docking into PTP1B model from AlphaFold 2.0. 53

Figure S8. Vermelhotin **6a2** docking into PTP1B model from AlphaFold 2.0. 54

Figure S9. Vermelhotin **6b1** docking into PTP1B model from AlphaFold 2.0.................... 55

Figure S10. Vermelhotin **6b2** docking into PTP1B model from AlphaFold 2.0.................... 56

Figure S11. Interactions prediction between PTP1B-ligand complexes in 2D for compounds **2**, **4** and **7–11**.................... 57

Figure S12. Interactions predictions the between PTP1B-ligand complexes for compounds **4**, **2**, and **7–11**.................... 58

Figure S13. SwissADME prediction for compounds **1–11** and positive controls.................... 59

Figure S14. 25 molecular fingerprints computed for the 47 most active NPs to build the SAS map between the evaluated compounds and UA. 66

Figure S15. Chemical structures of the compounds identified as **scaffold hops** in the map constructed with the tested compounds.................... 66

Table S1. Results of inhibitory activity using an *in vitro* assay of 99 natural products.

No	NAME	%I[1] (0.02 mM, 1 mM)	S	Compound type	Source/extract	Reference
1	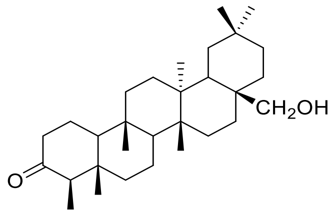<br>Canophyllol	95.54 ± 1.82<br>98.59 ± 0.28	MS	Triterpenoid friedelane	Stem bark and root of *Hippocratea excelsa* Kunth (syn.: *Semialarium mexicanum* (Miers) Mennega) (Celastraceae)<br>Hexane extract	Calzada, et al., 1991
2	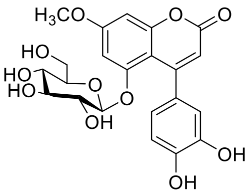<br>5-*O*-(*β*-D-Glucopyranosyl)-7-methoxy-3'-4'-dihydroxy-4-phenylcoumarin	42.48 ± 1.16<br>95.13 ± 0.54	S	4-Phenylcoumarin	Stem bark of *Hintonia latiflora* (Sessé & Moc. ex DC.) Bullock. (Rubiaceae)<br>CH_2Cl_2–MeOH (1:1) extract	Guerrero-Analco, et al., 2005
3	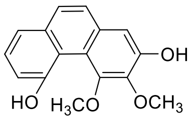<br>3,4-Dimethoxy-2,5-phenanthrenediol	70.62 ± 0.58<br>97.4 ± 0.69	MS	Phenanthrene	Whole plant of *Maxillaria densa* Lindl. (Orchidaceae)<br>$CHCl_3$-MeOH (1:1) extract	Estrada, et al., 1999

4	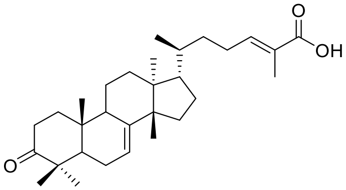<br>Masticadienonic acid	73.69 ± 2.31<br>111.54 ± 1.19	MS	Triterpenoid<br>tirucallane	Stem bark of *Amphipterygium adstringens* (Schltdl.) Standl. (Anacardiaceae)<br>CH_2Cl_2-MeOH (1:1) extract	Rivero-Cruz, et al., 2005
5	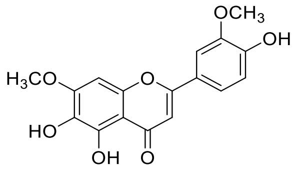<br>4',5,6-Trihydroxy-3',7-dimethoxyflavone	59.55 ± 1.18<br>104.4 ± 2.49	MS	Flavonoid<br>flavone	Aerial parts of *Salvia amarissima* Ortega (syn.: *Salvia circinnata* Cav. (Lamiaceae)<br>Ethyl acetate fraction from the infusion	Salinas-Arellano, et al., 2020
6	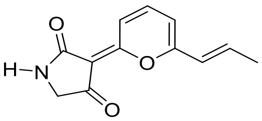<br>*E*/*Z*-vermelhotin	77.07 ± 0.13<br>92.82 ± 2.7	MS	Pyrrolidine-2,4-dione	Endophytic fungus MEXU 26343 isolated from leaves of *Hintonia latiflora* (Sessé et Moc. ex DC.) Bull. (Rubiaceae).<br>CH_2Cl_2 extract	Leyte-Lugo, et al., 2012

7	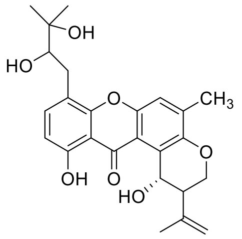 Tajixanthone hydrate	97.49 ± 1.58<br>97.86 ± 0.84	S	Xanthone	*Emericella* sp. strain 25379, isolated from the surface of a Coral<br>CH_2Cl_2 extract	Figueroa, et al., 2009
8	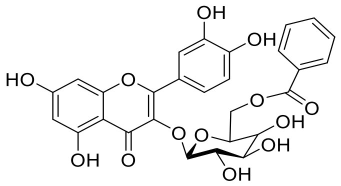 Quercetin-3-*O*-(6"-benzoyl)-*β*-D-galactoside	58.14 ± 2.26<br>96.82 ± 0.51	S	Flavonoid<br>Flavonol	Branches and leaves of *Vauquelinia corymbosa* Corrˆa ex Humb. & Bonpl. (Rosaceae)<br>Ethyl acetate fraction from the infusion	Flores-Bocanegra, et al., 2015
9	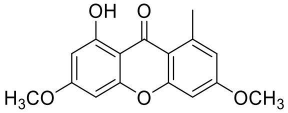 Lichexanthone	52.71 ± 0.53<br>95.37 ± 3.08	MS	Xanthone	Air-dried lichen material of *Parmotrema tinctorum* Nyl. (Hale) (Parmeliaceae)<br>$CHCl_3$-MeOH (1:1) extract	Rojas et al., 2000

10	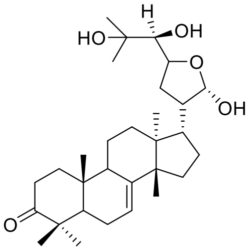<br>Melianodiol	57.08 ± 1.66<br>99.51 ± 0.99	MS	Protolimonoid	Seeds of *Guarea grandiflora* Steud. (syn.: *Guarea macrophylla* Vahl) (Meliaceae)<br>Hexane-$CHCl_3$ (1:1) extract	Jiménez, et al., 1998
11	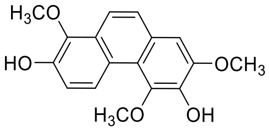<br>Confusarin	87.05 ± 0.91<br>99.89 ± 0.16	S	Phenanthrene	Pseudobulbs of *Cyrtopodium macrobulbon* (Lex.) G.A. Romero & Carnevali (Orchidaceae)<br>CH_2Cl_2 extract	Morales-Sánchez, et al., 2014
12	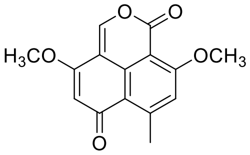<br>3,6-Dimethoxy-8-methyl-1*H*,6*H*-benzo[*de*]isochromene-1,9-dione	38.47 ± 7.36<br>65.94 ± 8.46	I	Polyketide<br>benzo[*de*]isochromene	*Sporormiella minimoides* S.I. Ahmed & Cain (Sporormiaceae), an endophytic fungus from *Hintonia latiflora* (Rubiaceae)<br>CH_2Cl_2 extract	Leyte-Lugo, et al., 2013

13	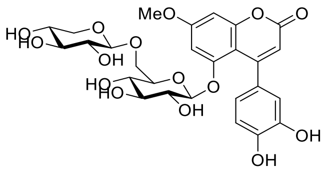<br>5-*O*-[*β*-D-Xylopyranosyl-(1→6)-*β*-D-glucopyranosyl]-7-methoxy-3',4'-dihydroxy-4-phenylcoumarin	50.14 ± 5.9<br>86.46 ± 3.32	S	4-Phenylcoumarin	Stem bark of *H. latiflora*<br>CH_2Cl_2–MeOH (1:1) extract	Guerrero-Analco, et al., 2007
14	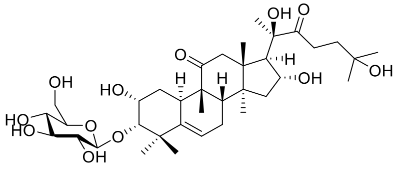<br>3-*O*-*β*-D-Glucopyranosyl-23,24-dihydrocucurbitacin F	56.13 ± 4.18<br>94.59 ± 3.14	MS	Triterpenoid cucurbitane	Stem bark of *H. standleyana*<br>CH_2Cl_2–MeOH (1:1) extract<br>2. Stem bark of *H. latiflora*<br>MeOH extract	Guerrero-Analco, et al., 2005<br>Mata, et al., 1990
15	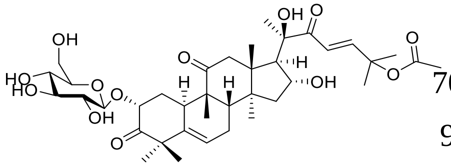<br>Arvenin I	70.15 ± 17.42<br>96.32 ± 2.63	S	Triterpenoid cucurbitane	Aerial parts of *Cigarrilla mexicana* (Zucc. & Mart. ex DC.) Aiello (syn.: *Nernstia mexicana* (Zucc. & Mart. ex DC.) Urb) (Rubiaceae)<br>MeOH extract	Mata, et al., 1988a

16	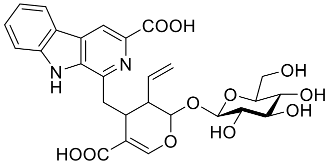<br>Desoxycordifolinic acid	55.44 ± 32.22<br>84.92 ± 19.42	S	Alkaloid	Stem bark of *H. standleyana*<br><br>CH_2Cl_2–MeOH (1:1) extract.	Déciga-Campos, et al., 2006
17	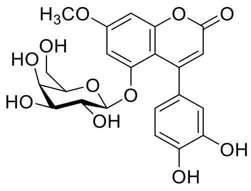<br>5-*O*-*β*-D-Galactopyranosyl-7-methoxy-3',4'-dihydroxy-4-phenylcoumarin	6.03 ± 0.85<br>14.48 ± 0.9	S	4-Phenylcoumarin	Stem bark of *H. standleyana*<br><br>CH_2Cl_2–MeOH extract.	Cristians, et al., 2009
18	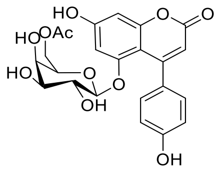<br>6''-*O*-Acetyl-5-*O*-*β*-D-galactopyranosyl-7,4'-dihydroxy-4-phenylcoumarin	2.68 ± 37.39<br>95.87 ± 0.41	S	4-Phenylcoumarin	Stem bark of *H. standleyana*<br><br>CH_2Cl_2–MeOH extract.	Cristians, et al., 2009
19	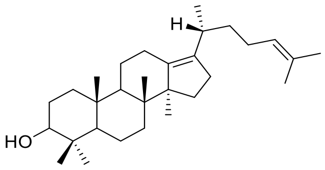<br>20(*S*)-dammar-13(17),24-diene-3*β*-ol	1.61 ± 3.58<br>22.01 ± 7.7	S	Triterpenoid<br>dammarane	Synthetic analog of 20(*S*)-dammar-13(17),24-diene-3*β*-yl acetate (Isolated of *Stevia salicifolia* Car.)	Mata, et al., 1991a

20	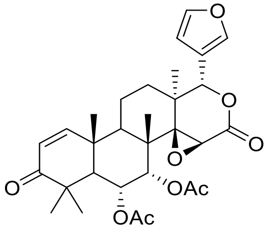<br>6α-Acetoxygedunin	31.66 ± 3.43<br>49.04 ± 7.17	MS	Limonoid	Seeds of *G. grandiflora*<br>Hexane-$CHCl_3$ (1:1) extract	Jimenez, et al., 1998
21	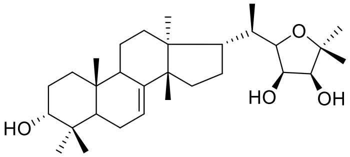<br>Odoratol	87.52 ± 7.11<br>90.19 ± 2.94	I	Protolimonoid	Wood of *Cedrela odorata* L. (Meliaceae)<br>$CHCl_3$-MeOH (1:1) extract	Achnine, et al., 1998
22	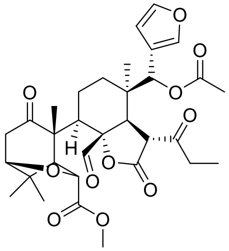<br>Cedrelanolide I	-29.83 ± 7.95<br>27.67 ± 12.75	I	Limonoid	Stem bark *Cedrela salvadorensis* Standl. (Meliaceae).<br>$CHCI_3$ extract	Segura, et al., 1994
23	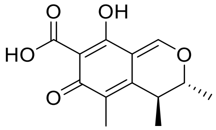<br>Citrinin	35.88 ± 4.92<br>79.83 ± 0.88	MS	Polyketide	Coprophilous Fungus *Guanomyces polythrix* M.C. González, Hanlin & Ulloa (Chaetomiaceae)	Macias, et al., 2000

24	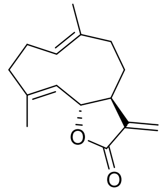 Costunolide	72.56 ± 1.3<br>88.22 ± 4.51	MS	Sesquiterpene lactone	CH_2Cl_2 extract<br>Roots of *Cosmos pringlei* B.L.Rob. & Fernald (Asteraceae)<br>CH_2Cl_2-MeOH extract (1:1)	Mata, et al., 2002
25	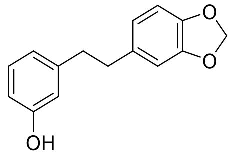 Phenol, 3-[2-(1,3-benzodioxol-5-yl)ethyl]-]	35.82 ± 3.52<br>107.56 ± 2.58	MS	Stilbenoid	Synthetic analog of gigantol	Reyes-Ramírez, et al., 2011
26	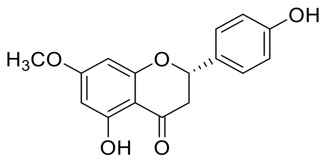 Sakuranetin	36.49 ± 0.04<br>59.88 ± 10.2	MS	Flavonoid<br>flavanone	Aereal parts *of Dodonaea viscosa* (L.) Jacq. (Sapindaceae)<br>$CHCl_3$-MeOH (1:1) extract	Mata et al., 1991b
27	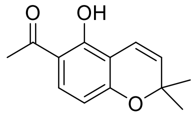 Desmethylisoencecalin	-7.18 ± 5.46<br>36.08 ± 11.99	MS	Chromene	Aerial parts of *Calea ternifolia* Kunth (Asteraceae)<br>CH_2Cl_2–MeOH (1:1) extract	Escandon-Rivera, et al., 2017

28	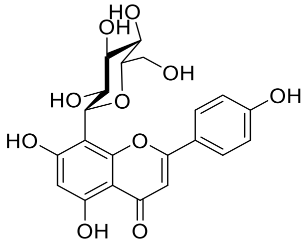<br>Vitexin	53.3 ± 7.41<br>80.18 ± 5.15	S	Flavonoid<br>flavone	1. Whole plant of *Nidema boothii* (Lindl.) Schltr. (Orchidaceae)<br>CH_2Cl_2–MeOH (1:1) extract<br>2. Whole plant of *Epidendrum rigidum* Jacq. (Orchidaceae).<br>$CHCl_3$–MeOH (1:1) extract	Hernández-Romero, et al., 2004<br>Hernández-Romero, et al., 2005
29	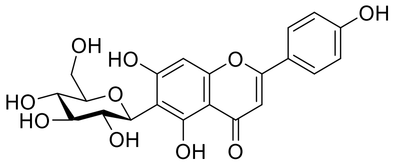<br>Isovitexin	52.26 ± 1.11<br>74.76 ± 4.32	S	Flavonoid<br>flavone	2. Whole plant of *E. rigidum*<br>$CHCl_3$–MeOH (1:1) extract	Hernández-Romero, et al., 2005
30	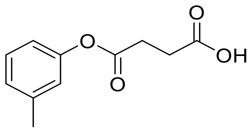<br>Butanedioic acid,1-(3-methylphenyl) ester	34.01 ± 3.25<br>67.66 ± 3.76	S	Phenyl succinate	A reaction intermediate in synthesis of derivatives.	Reyes-Ramírez, et al., 2011
31	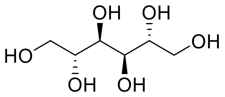<br>Mannitol	26.3 ± 6.56<br>69.08 ± 5.84	S	Polyol	Stem bark of *Exostema caribaeum* (Jacq.)	Mata, et al., 1987

					Roem. et Schult. (Rubiaceae).<br>Methanol extract	
32	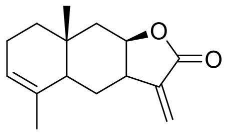<br>Isoalloalantolactone	58.33 ± 4.07<br>100.01 ± 0.3	MS	Sesquiterpene lactone	Roots of *Ratibida mexicana* (S.Watson) W. M. Sharp (Asteraceae)<br>Methanol extract	Calera, et al., 1995
33	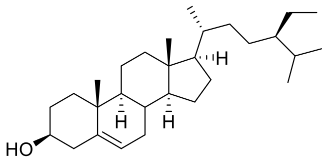<br>β-Spinasterol	12.5 ± 1.39<br>62.5 ± 7.34	I	Steroid	Roots of *Cosmos pringlei* B.L.Rob. & Fernald (Asteraceae)<br>CH_2Cl_2-MeOH extract (1:1)	Mata, et al., 2002
34	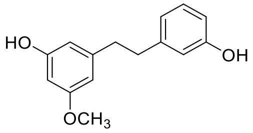<br>Batatasin III	47.23 ± 1.15<br>98.01 ± 0.9	S	Stilbenoid	Pseudobulbs of *C. macrobulbon*<br>CH_2Cl_2 extract	Morales-Sánchez, et al., 2014
36	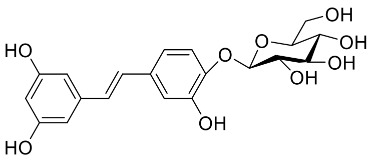<br>Resveratrol 4'-*O*-*β*-D-glucopyranoside	24.44 ± 3.6<br>41.52 ± 3.52	S	Stilbenoid	Roots of *Rumex hymenosepalus* Torr. (Polygonaceae)<br>CH_2Cl_2-MeOH (1:1) extract	Rivero-Cruz, et al., 2005

37	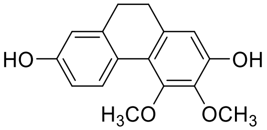 Erianthridin	20.90 ± 3.04<br>27.89 ± 4.53	MS	Phenanthrene	Whole plant of *M. densa*<br>$CHCl_3$-MeOH (1:1) extract	Estrada et al., 1999
38	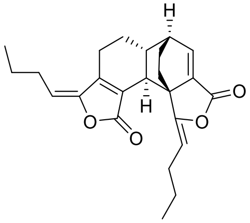 Diligustilide	32.75 ± 2.47<br>60.3 ± 4.69	I	Phthalide	Roots of *Ligusticum porteri* J.M. Coult. & Rose (Apiaceae).<br>CH_2Cl_2−MeOH extract	Brindis, et al., 2011
39	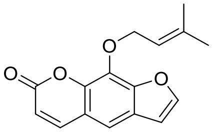 Imperatorin	37.96 ± 2.01<br>90.3 ± 0.12	S	Furanocoumarin	Aerial parts of *Esenbeckia yaaxhokob* Lundell (Rutaceae)<br>CH_2Cl_2-MeOH extract	Mata, et al., 1998
40	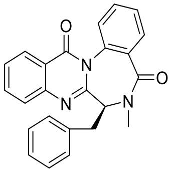 Benzomalvin A	92.43 ± 1.98<br>99.55 ± 0.36	MS	Tripeptide	fungus *Penicillium spathulatum* Frisvad & Samson (Trichocomaceae).<br>CH_2Cl_2-MeOH extract	Del Valle, et al., 2016

41	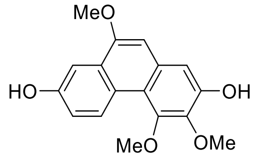<br>Gymnopusin	43.78 ± 0.45<br>97.14 ± 0.44	MS	Phenanthrene	Whole plant of *M. densa*<br>$CHCl_3$–MeOH (1:1) extract	Valencias-Islas, et al., 2002<br>Estrada, et al., 1999
42	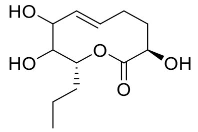<br>Herbarumin II	28.6 ± 1.02<br>63.05 ± 2.4	S	Nonenolide	Fungus *Phoma herbarum* Westend (Sphaeropsidaceae) [syn. *Phoma pigmentivora* Massee]<br>Ethyl acetate extract	Rivero-Cruz, et al., 2000
43	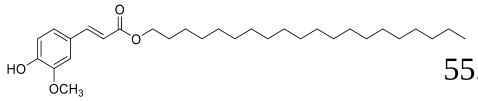<br>Eicosyl 4-hydroxy-3-methoxy-cinnamate	55.82 ± 3.24<br>98.68 ± 0.87	S	Phenylpropanoid	Whole plant of *M. densa*<br>$CHCl_3$–MeOH (1:1) extract	Rodriguez Lizana, 1998
44	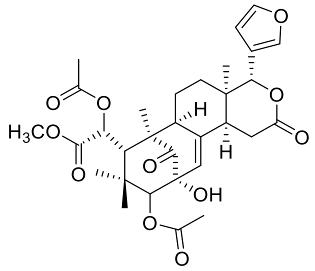<br>Humilinolide D	53.4 ± 1.26<br>84.38 ± 5.17	I	Limonoid	Seeds of *Swietenia humilis* Zucc. (Meliaceae)<br>MeOH extract	Segura-Correa, et al, 1993

45	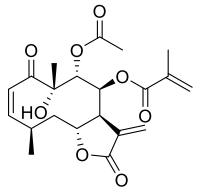<br>Calein C	39.05 ± 2.32<br>77.16 ± 0.87	I	Sesquiterpene lactone	Aerial parts of *C. ternifolia*<br>CH_2Cl_2–MeOH extract (1:1)	Escandón-Rivera, et al., 2017
46	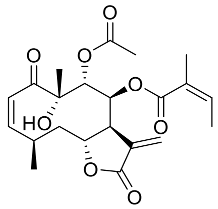<br>Calein A	65.87 ± 1.34<br>95.15 ± 3.35	MS	Sesquiterpene lactone	Aerial parts of *C. ternifolia*<br>CH_2Cl_2–MeOH extract (1:1)	Escandón-Rivera, et al., 2017
47	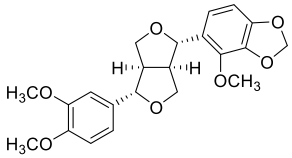<br>2'-Methoxykobusin	94.02 ± 1.01<br>93.84 ± 0.86	MS	Lignane	Whole plant of *Leucophyllum ambiguum* Bonpl (Scrophulariaceae)<br>CH_2Cl_2-MeOH (1:1) extract	Rojas, et al., 2003
48	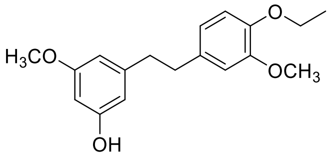<br>3-[2-(4-Ethoxy-3-methoxyphenyl)ethyl]-5-methoxyphenol	89.36 ± 1.58<br>92.72 ± 1.96	I	Stilbenoid	Synthetic analog of gigantol	Reyes-Ramírez, et al., 2011

49	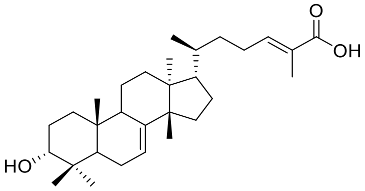 3α-Hydroxymasticadienonic acid	90.32 ± 1.21<br>91.06 ± 1.34	MS	Triterpenoid<br>tirucallane	Stem bark of *A. adstringens* (Anacardaceae)<br>CH_2Cl_2-MeOH (1:1) extract	Rivero-Cruz, et al., 2005
50	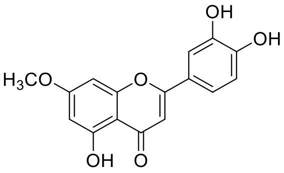 7-Methylluteolin	88.59 ± 2.25<br>95.08 ± 2.94	MS	Flavonoid<br>flavone	Stem bark of *H. latiflora*<br>Methanol extract	Mata, et al., 1990
51	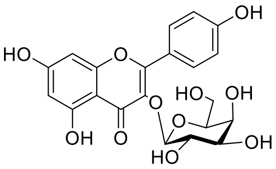 Trifolin	95.67 ± 2.71<br>124.76 ± 0.4	MS	Flavonoid<br>flavonol	Leaves of *Hydrangea seemannii* L. Riley (Hydrangeaceae)<br>Ethyl acetate fraction from the infusion	Pérez-Vásquez, et al., 2020
52	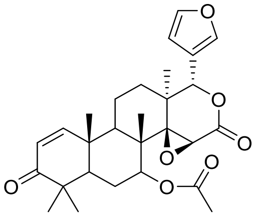 Gedunin	71.44 ± 2.45<br>73.43 ± 5.94	MS	Limonoid	Seeds of *G. grandiflora*<br>Hexane-$CHCl_3$ (1:1) extract	Jimenez, et al., 1998

53	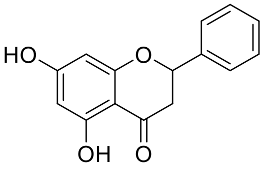<br>Pinocembrin	86.62 ± 3.45<br>96.61 ± 1.4	MS	Flavonoid<br>flavanone	Aerial parts of *Chenopodium graveolens* Willd. (syn.: *Dysphania graveolens* Mosyakin & Clemants) (Amaranthaceae)<br>$CHCl_3$ extract	Mata, et al., 1986
54	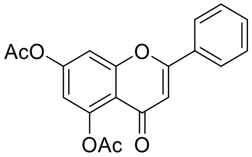<br>5,7-Diacetylchrysin	79.03 ± 1.31<br>97.81 ± 1.49	MS	Flavonoid<br>flavone	Aerial parts of *C. graveolens*<br>$CHCl_3$ extract	Mata, et al., 1986
55	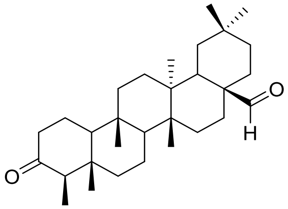<br>Canophyllal	92.91 ± 1.49<br>77.23 ± 3.48	MS	Triterpenoid<br>friedelane	Stem bark and root of *H. excelsa*<br>Hexane extract	Calzada, et al., 1991
56	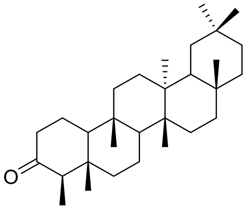<br>Friedelin	84.92 ± 2.89<br>104.67 ± 2.08	MS	Triterpenoid<br>friedelane	Stem bark and root of *H. excelsa*<br>Hexane extract	Calzada, et al., 1991

57	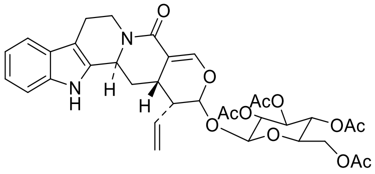<br>Tetraacetyl strictosamide	86.82 ± 0.84<br>109.16 ± 1.6	I	Alkaloid	Synthetic analog of strictosamide isolated of *Simira mexicana* (Bullock) Steyerm. (Rubiaceae)	Albor Calderon, 1989
58	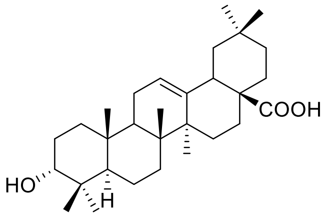<br>3-*epi*-Oleanolic acid	95.61 ± 1.32<br>105.54 ± 3.06	MS	Triterpenoid<br>oleanane	Leaves of *Celaenodendron mexicanum* Standl.<br>(Syn.: *Piranhea mexicana* (Standl.) Radcl.-Sm. (Picrodendraceae)<br>$CHCl_3$-MeOH (1:1) extract	del Rayo Camacho, et al., 2000
59	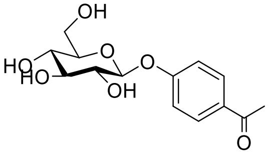<br>Picein	93.78 ± 0.7<br>94.81 ± 1.1	S	Phenolic glycoside	Branches and leaves of *V. corymbosa*<br>Ethyl acetate fraction from the infusion	Flores-Bocanegra, et al., 2015
60	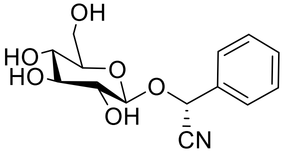<br>Prunasin	93.05 ± 0.88<br>85.42 ± 2.96	S	Cyanogenic glycoside	Branches and leaves of *V. corymbosa*<br>Ethyl acetate fraction from the infusion	Flores-Bocanegra, et al., 2015

61	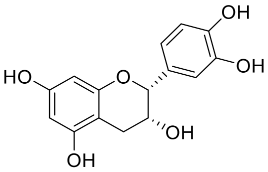<br>(−)-Epicatechin	68.74 ± 1.73<br>89.39 ± 2.54	S	Flavonoid<br>2,3-*cis*-flavan-3-ol	Branches and leaves of *V. corymbosa*<br>Ethyl acetate fraction from the infusion	Flores-Bocanegra, et al., 2015
62	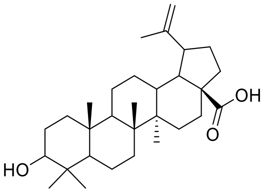<br>Betulinic acid	95.19 ± 1.06<br>93.31 ± 3.31	S	Triterpenoid<br>lupane	Branches and leaves of *V. corymbosa*<br>Ethyl acetate fraction from the infusion	Flores-Bocanegra, et al., 2015
63	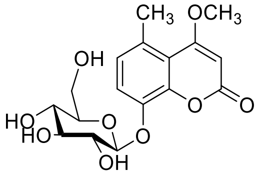<br>8-*β*-D-glucopyranosyloxy-4-methoxy-5-methyl-coumarin	96.52 ± 0.15<br>99.24 ± 0.65	S	Coumarin	Roots of *Acourtia thurberi* (A. Gray) Reveal & R.M. King (Asteraceae)<br>Hexane extract	Martínez, et al., 2017
64	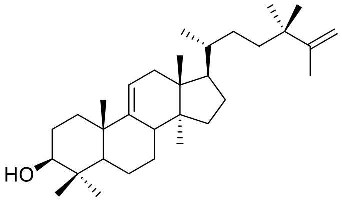<br>5*α*-lanosta-24,24-dimethyl-9(11),25-dien-3*β*-ol	9.94 ± 6.31<br>95 ± 5.84	MS	Triterpenoid<br>lanostane	Whole plants of *Scaphyglottis livida* (Lindley) Schltr. (Orchidaceae)<br>$CHC1_3$–MeOH (1:1) extract	Estrada, et al., 2002

65	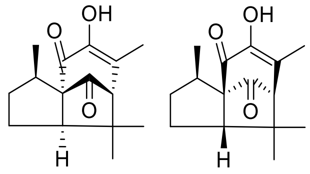 α/β-Pipitzol	-3.62 ± 0.6<br>62.11 ± 16.44	MS	Sesquiterpenoids	Roots of *A. thurberi*<br>Hexane extract	Martínez, et al., 2017
66	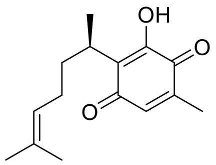 Perezone	84.45 ± 4.27<br>88.44 ± 4.03	I	Sesquiterpenoid	Roots of *A. thurberi*<br>Hexane extract	Martínez, et al., 2017
67	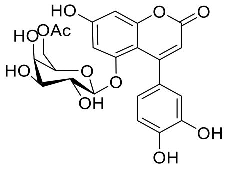 6"-*O*-acetyl-5-*O*-*β*-D-galactopyranosyl-7,3',4'-trihydroxy-4-phenylcoumarin	92.6 ± 1.51<br>95.84 ± 0.36	S	4-Phenylcoumarin	Stem bark of *E. caribaeum*<br>MeOH extract	Mata, et al., 1987
68	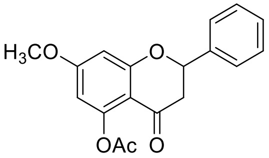 5-*O*-Acethyl pinostrobin	-5.26 ± 13.31<br>65.71 ± 1.78	MS	Flavonoid flavanone	Synthetic analog of Pinostrobin, isolated from *C. graveolens*	Mata, et al., 1986

69	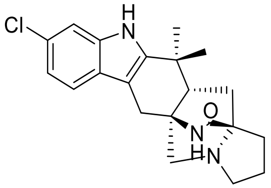<br>Malbrancheamide B	87.88 ± 10.41<br>122.02 ± 8.79	I	Alkaloid	Fungal strain *Malbranchea aurantiaca* Sigler & Carmichael (Myxotrichaceae)<br><br>CH_2Cl_2 extract	Figueroa, et al., 2008
70	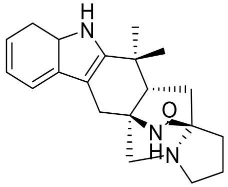<br>Premalbrancheamide	66.73 ± 2.72<br>95.45 ± 0.67	S	Alkaloid	*M. aurantiaca*<br><br>CH_2Cl_2 extract	Figueroa, et al., 2008
71	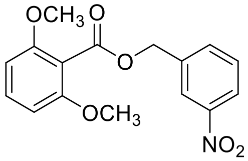<br>3'-Nitrobenzyl 2,6-dimethoxybenzoate	-16.77 ± 4.63<br>91.9 ± 0.39	MS	Benzyl benzoate	Synthetic analog of benzyl benzoates from *Brickellia veronicifolia*	Rivero-Cruz, et al., 2007
72	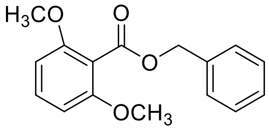<br>Benzyl 2,6-dimethoxybenzoate	3.24 ± 0.79<br>92.49 ± 0.88	MS	Benzyl benzoate	Aerial parts of *Brickellia veronicifolia* (Kunth) A. Gray (Asteraceae)	Rivero-Cruz, et al., 2007

					CH_2Cl_2-MeOH (1:1) extract	
73	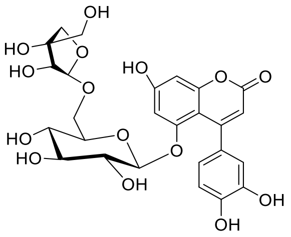<br>5-*O*-*β*-D-Apiofuranosyl-(1→6)-*β*-D-glucopyranosyl-7-metoxi-3'-4'-dihydroxy-4-phenylcoumarin	57.56 ± 3.8<br>95 ± 0.54	S	4-Phenylcoumarin	Stem bark of *H. standleyana*<br><br>CH_2Cl_2–MeOH extract	Guerrero-Analco, et al., 2005
74	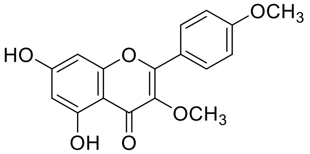<br>Kaempferol 3,4'-dimethylether	38.46 ± 3.2<br>92.85 ± 0.77	I	Flavonoid<br>flavonol	Leaves of *Hyptis albida* Kunth (Lamiaceae)	Rojas, et al., 1992
75	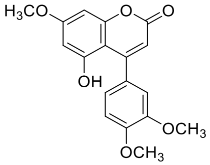<br>7,3',4'-trimethoxy-4-phenylcoumarin	56.07 ± 12.42<br>94.96 ± 1.85	MS	4-Phenylcoumarin	Semisynthetic derivative prepared by hydrolysis	Mata, et al., 1987

76	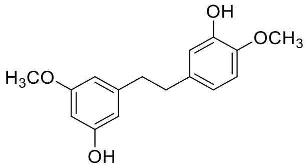 Gigantol	65.42 ± 2.33<br>54.01 ± 3.49	S	Stilbenoid	Whole plants of *S. livida*<br><br>$CHCl_3$-MeOH (1:1) extract	Estrada, et al., 1999
77	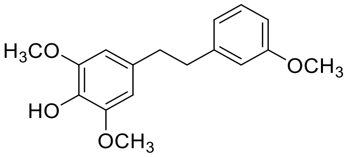 4-Hydroxy-3,3',5-trimetoxybibencyl	93.48 ± 0.35<br>100.21 ± 0.78	MS	Stilbenoid	Synthetic analog of gigantol	Reyes-Ramirez, et al., 2011<br>Hernández-Romero, et al., 2005
78	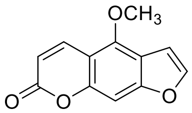 5-Methoxypsoralen	65.64 ± 2.64<br>49.04 ± 3.35	MS	Furanocoumarin	Aerial parts of *Arracacia tolucensis* var. *multifida* (S. Watson) Mathias & Constance (Apiaceae)<br><br>CH_2Cl_2–MeOH (1:1) extract	Figueroa, et al., 2007
79	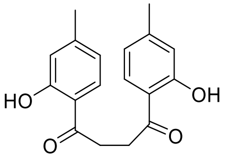 Hofmeisterin II	25.07 ± 2.90<br>79.09 ± 1.79	MS	Thymol derivative	Aerial parts of *Hofmeisteria schaffneri* (A. Gray) R. M. King & H. Rob. (Asteraceae)	Pérez-Vásquez, et al., 2005

80	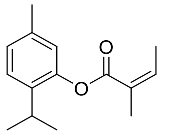<br>Thymol angelate	58.17 ± 1.4<br>46.58 ± 1.79	S	Thymol derivative	CH_2Cl_2-MeOH (1:1) extract<br>Aerial parts of *H. schaffneri*<br>CH_2Cl_2-MeOH (1:1) extract	Pérez-Vásquez, et al., 2005
81	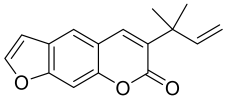<br>Chalepensin	58.75 ± 4.31<br>34.88 ± 2.87	MS	Furanocoumarin	Roots of *Stauranthus perforatus* Liebm. (Rutaceae)<br>$CHCl_3$–MeOH (1:1) extract	Anaya, et al., 2005
82	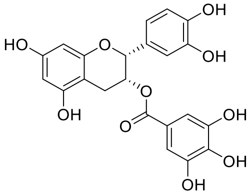<br>(-)-Epicatechin 3-*O*-gallate	94.85 ± 0.5<br>96.69 ± 0.83	S	Flavonoid<br>2,3-*cis*-flavan-3-ol	Roots of *R. hymenosepalus*<br>CH_2Cl_2–MeOH extract	Rivero-Cruz, et al., 2005
83	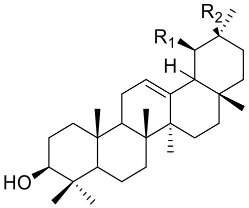	64.07 ± 3.99<br>98.47 ± 1.39	I	Triterpenoids<br>Ursane/oleanane	Root bark of *H. excelsa*<br>Methanol extract	Navarrete, et al., 2002

	α: R_1= CH_3, R_2= H<br>β: R_1= H, R_2= CH_3<br>*α*/*β*-Amyrin					
84	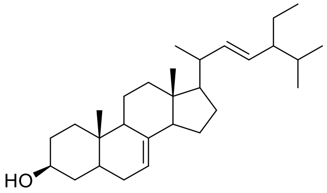<br>*α*-Spinasterol	63.12 ± 1.16<br>96.6 ± 0.44	MS	Sterol	Aerial parts of *C. filaginoides*<br><br>$CHCl_3$-MeOH extract	Mata, et al., 1997
85	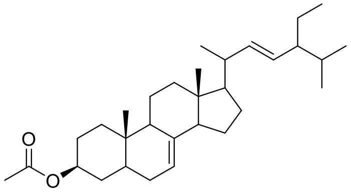<br>*α*-Spinasterol acetate	91.33 ± 2.67<br>99.95 ± 1.66	MS	Sterol	Synthetic analog of α-Spinasterol isolated of *C. filaginoides*	Mata, et al., 1997
86	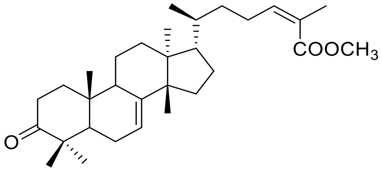<br>Methyl ester of masticadienonic acid	96.94 ± 0.73<br>100.71 ± 0.43	S	Triterpenoid<br>tirucallane	Stem bark of *A. adstringens*<br><br>Hexane extract	Rivero-Cruz, et al., 2005
87	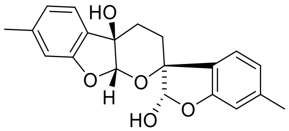	49.48 ± 1.63<br>70.93 ± 4.61	S	Thymol derivative	Aerial parts of *H. schaffneri*	Pérez-Vásquez, et al., 2005

	3',4',4a',9a'-tetrahydro-6,7'-dimethylspiro[benzofuran-3(2*H*),2'-pyrano[2,3-*b*]benzofuran]-2,4a'-diol				CH_2Cl_2-MeOH (1:1) extract	
88	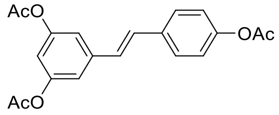 Acetyl analog of 5-[(*E*)-2-(4-hydroxyphenyl)ethenyl]-1,3-benzenediol	34.54 ± 2.42<br>45.12 ± 7.09	I	Stilbenoid	Synthetic analog of resveratrol 4'-*O*-*β*-D-glucopyranoside isolated of *R. hymenosepalus*	Rivero-Cruz, et al., 2005
89	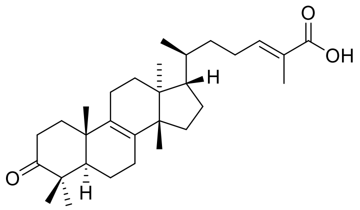 Isomasticadienonic acid	96.19 ± 0.73<br>101.05 ± 0.48	MS	Triterpenoid<br>lanostane	Stem bark of *A. adstringens*<br><br>CH_2Cl_2-MeOH extract	Rivero-Cruz, et al., 2005
90	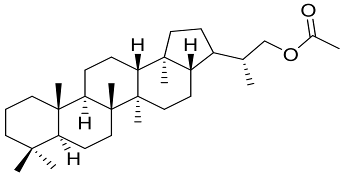 21*α*-*H*-Hopane acetate	89.16 ± 0.46<br>98.02 ± 1.36	MS	Triterpenoid<br>lupane	*P. tinctorum*<br><br>$CHCl_3$-MeOH (1:1) extract	Rojas et al., 2002
91	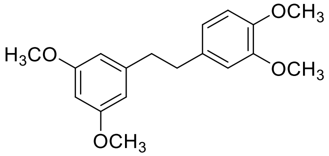	58.72 ± 3.16<br>95.13 ± 2.43	MS	Stilbene	Synthetic analog of gigantol using Wittig reaction	Hernández-Romero, et al., 2005

	3,3',4',5-Tetramethoxybibenzyl					
92	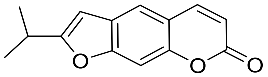 2'-Isopropyl psoralene	96.35 ± 0.96<br>90.78 ± 1.28	MS	Furanocoumarin	Roots of *S. perforatus* L<br>$CHCl_3$–MeOH (1:1) extract	Anaya, et al., 2005
93	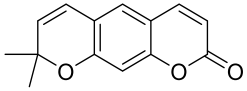 Xanthyletin	67.34 ± 2.72<br>96.99 ± 1.06	MS	Pyranocoumarin	Roots of *S. perforatus*<br>$CHCl_3$–MeOH (1:1) extract	Anaya, et al., 2005
94	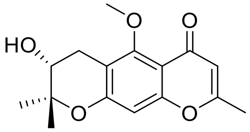 (+)-5-Methoxyhamaudol	92.6 ± 1.36<br>96.54 ± 0.68	S	Pyranochromone	Synthetic analog of (-)-hamaudol	Valencia-Islas, et al., 2002
95	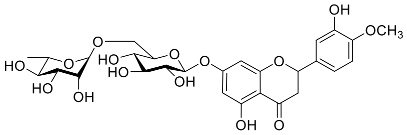 Hesperidin	88.62 ± 1.54<br>84.62 ± 0.47	S	Flavonoid<br>flavanone	Aerial parts of *E. yaaxhokob*<br>CH_2Cl_2-MeOH (1:1) extract	Mata, et al., 1998
96	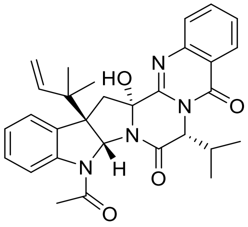 Albolutein A		S	Alkaloid<br>Diketopiperazine	Fungal strain *Malbranchea albolutea* Sigler & J.W.Carmich. (Onygenaceae)<br>AcOEt extract	Díaz-Rojas, et al., 2021

97	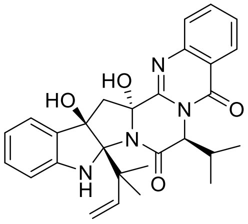 Albolutein B		S	Alkaloid Diketopiperazine	*M. albolutea* AcOEt extract	Díaz-Rojas, et al., 2021
98	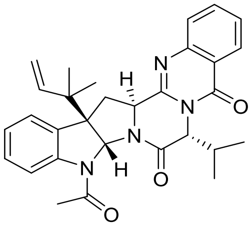 Albolutein C		S	Alkaloid Diketopiperazine	*M. albolutea* AcOEt extract	Díaz-Rojas, et al., 2021
99	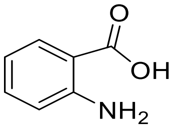 *o*-Anthranilic acid		S	*o*-Aminobenzoic acid	*M. albolutea* AcOEt extract	Díaz-Rojas, et al., 2021
C[2]	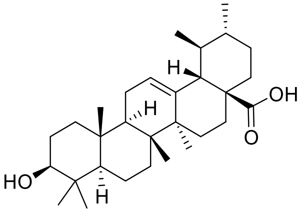 Ursolic acid	77.94 ± 0.89<br>96.67 ± 0.53	S	Triterpene ursane	Branches and leaves of *V. corymbosa*<br>Ethyl acetate fraction from infusion	Flores-Bocanegra, et al., 2015

References of Table S1 listed in alphabetical order.

Achnine, L., Mata, R., Iglesias-Prieto, R., Lotina-Hennsen, B. (**1998**). Impairment of photosystem II donor side by the natural product odoratol. Journal of agricultural and food chemistry, 46, 5313−5317.

Albor Calderón C. **1989**. Estudio fitoquímico de tres rubiaceas usadas en medicina tradicional: *Randia echinocarpa*, *Exostema mexicanum* y *Simira mexicana*. Tesis de Maestría. Universidad Nacional Autónoma de Mexico, Mexico.

Anaya, A. L., Macías-Rubalcava, M., Cruz-Ortega, R., García-Santana, C., Sánchez-Monterrubio, P. N., Hernández-Bautista, B. E., Mata, R. (**2005**). Allelochemicals from *Stauranthus perforatus*, a Rutaceous tree of the Yucatan Peninsula, Mexico. Phytochemistry, 66, 487−494.

Brindis, F., Rodríguez, R., Bye, R., González-Andrade, M., Mata, R. (**2011**). (Z)-3-butylidenephthalide from *Ligusticum porteri*, an α-glucosidase inhibitor. Journal of natural products, 74, 314−320.

Calera, M. R., Soto, F., Sanchez, P., Bye, R., Hernandez-Bautista, B., Anaya, A. L., Lotina-Hennsen B., Mata, R. (**1995**). Biochemically active sesquiterpene lactones from *Ratibida mexicana*. Phytochemistry, 40, 419−425.

Calzada, F., Mata, R., López, R., Linares, E., Bye, R., Barreto, V., del Rio, F. (**1991**). Friedelanes and Triterpenoid Quinone Methides from *Hippocratea exclesa*1. Planta Medica, 57, 194–195.

Cristians, S., Guerrero-Analco, J. A., Pérez-Vásquez, A., Palacios-Espinosa, F., Ciangherotti, C., Bye, R., Mata, R. (2009). Hypoglycemic activity of extracts and compounds from the leaves of *Hintonia standleyana* and *H. latiflora*: potential alternatives to the use of the stem bark of these species. Journal of Natural Products, 72, 408−413.

Déciga-Campos, M., Guerrero-Analco, J. A., Quijano, L., Mata, R. (**2006**). Antinociceptive activity of 3-O-β-D-glucopyranosyl-23, 24-dihydrocucurbitacin F from *Hintonia standleyana* (Rubiaceae). Pharmacology Biochemistry and Behavior, 83, 342−348.

Del Valle, P., Martínez, A. L., Figueroa, M., Raja, H. A., Mata, R. (**2016**). Alkaloids from the fungus *Penicillium spathulatum* as α-glucosidase inhibitors. Planta medica, 82, 1286−1294.

Del Rayo Camacho, M., Mata, R., Castaneda, P., Kirby, G. C., Warhurst, D. C., Croft, S. L., Phillipson, J. D. (**2000**). Bioactive compounds from *Celaenodendron mexicanum*. Planta medica, 66, 463−468.

Díaz-Rojas, M., Raja, H., González-Andrade, M., Rivera-Chávez, J., Rangel-Grimaldo, M., Rivero-Cruz, I., Mata, R. (**2021**). Protein tyrosine phosphatase 1B inhibitors from the fungus *Malbranchea albolutea*. Phytochemistry, 184, 112664.

Escandón-Rivera, S., Pérez-Vásquez, A., Navarrete, A., Hernández, M., Linares, E., Bye, R., Mata, R. (**2017**). Anti-hyperglycemic activity of major compounds from Calea ternifolia. Molecules, 22, 289.

Estrada, S., Toscano, R. A., Mata, R. (**1999**). New Phenanthrene Derivatives from *Maxillaria densa*. Journal of natural products, 62, 1175−1178.

Estrada S., Rojas A., Mathison Y., Israel A., Mata R. (**1999**). (**+**). Nitric oxide/cGMP mediates the spasmolitic action of 3,4′-dihydroxy-5,5′-dimethoxybibenzyls from *Scaphyglottis livida*. Planta Medica, 65, 109−114.

Estrada, S., Acevedo, L., Rodriguez, M., Toscano, R., Mata, R. (**2002**). New triterpenoids from the orchids Scaphyglottis livida and Nidema boothii. Natural Product Letters, 16, 81−86.

Flores-Bocanegra L, Pérez-Vásquez A, Torres-Piedra M, Bye R, Linares E, Mata R. α-Glucosidase Inhibitors from *Vauquelinia corymbosa*. *Molecules*. 2015; 20(8):15330-15342.

Figueroa M., Rivero-Cruz I., Rivero-Cruz B., Bye R., Navarrete A., Mata R. (**2007**). Constituents, biological activities and quality control parameters of the crude extract and essential oil from *Arracacia tolucensis* var. *multifida*, Journal of Ethnopharmacology, 113, 125−131.

Figueroa M., González M. del C., Rachel Mata (**2008**). Malbrancheamide B, a novel compound from the fungus *Malbranchea aurantiaca* , *Natural Product Research*, 22, 709−714.

Figueroa, M., González, M. del C., Rodríguez-Sotres, R., Sosa-Peinado, A., González-Andrade, M., Cerda-García-Rojas, C. M., Mata, R. (**2009**). Calmodulin inhibitors from the fungus *Emericella* sp. *Bioorganic & Medicinal Chemistry*, 17, 2167–2174.

Guerrero-Analco, J. A., Hersch-Martinez, P., Pedraza-Chaverri, J., Navarrete, A., Mata, R. (2005). Antihyperglycemic effect of constituents from *Hintonia standleyana* in streptozotocin-induced diabetic rats. Planta Medica, 71, 1099−1105.

Guerrero-Analco, J., Medina-Campos, O., Brindis, F., Bye, R., Pedraza-Chaverri, J., Navarrete, A., Mata, R. (**2007**). Antidiabetic properties of selected Mexican copalchis of the Rubiaceae family. Phytochemistry, 68, 2087−2095.

Hernández-Romero, Y., Rojas, J. I., Castillo, R., Rojas, A., Mata, R. (**2004**). Spasmolytic Effects, Mode of Action, and Structure− Activity Relationships of Stilbenoids from *Nidema boothii*. Journal of natural products, 67, 160−167.

Hernández-Romero, Y., Acevedo, L., de Los Ángeles Sánchez, M., Shier, W. T., Abbas, H. K., Mata, R. (**2005**). Phytotoxic activity of bibenzyl derivatives from the orchid *Epidendrum rigidum*. Journal of agricultural and food chemistry, 53, 6276−6280.

Jimenez, A., Villarreal, C., Toscano, R. A., Cook, M., Arnason, J. T., Bye, R., Mata, R. (**1998**). Limonoids from *Swietenia humilis* and *Guarea grandiflora* (Meliaceae). *Phytochemistry*, 49, 12981−1988.

Leyte-Lugo, M., González-Andrade, M., González, M. del C., Glenn, A. E., Cerda-García-Rojas, C. M., & Mata, R. (2012). (+)-Ascosalitoxin and Vermelhotin, a Calmodulin Inhibitor, from an Endophytic Fungus Isolated from *Hintonia latiflora*. Journal of Natural Products, 75, 1571–1577.

Leyte-Lugo M., Mario Figueroa, María del Carmen González, Anthony E. Glenn, Martín González-Andrade, Rachel Mata,'Corrigendum to "Metabolites from the endophytic fungus Sporormiella minimoides isolated from *Hintonia latiflora*" [Phytochemistry 96 (2013) 273–278]', Phytochemistry, 98, 2014.

Macías, M., Ulloa, M., Gamboa, A., Mata, R. (**2000**). Phytotoxic compounds from the new coprophilous fungus *Guanomyces polythrix*. Journal of natural products, 63, 757−761.

Martínez, A. L., Madariaga-Mazón, A., Rivero-Cruz, I., Bye, R., Mata, R. (2017). Antidiabetic and antihyperalgesic effects of a decoction and compounds from *Acourtia thurberi*. Planta Medica, 234, 534−544.

Mata R., Navarrete A., Alvarez A., Pereda-Miranda R., Delgado G., Romo de Vivar A. (**1986**). Flavonoids and terpenoids of *Chenopodium graveolens*, Phytochemistry, 26, 191−193.

Mata, R., Calzada, F., Garcia, M. R., Reguero, M. T. (**1987**). Chemical studies on mexican plants used in traditional medicine, III: New 4-phenylcoumarins from *Exostema caribaeum*. Journal of natural products, 50, 866−871.

Mata, R., Castañeda, P., Camacho, M. D. R., Delgado, G. (**1988**). Chemical studies on Mexican plants used in traditional medicine, V. Cucurbitacin glucosides from *Cigarrilla mexicana*. *Journal of natural products*, *51*, 836−839.

Mata R., Camac Camacho M., Cervera E., Bye R., Linares E. (**1990**). Secondary metabolites from *Hintonia latiflora*, Phytochemistry, 29, 2037−2040.

Mata, R., Pereda-Miranda, R., Bye, R., Linares, E. (**1991a**). A dammarane from *Stevia salicifolia*. Phytochemistry, 30, 3822−3823.

Mata, R., Contreras, J. L., Crisanto, D., Pereda-Miranda, R., Castañeda, P., Del Rio, F. (**1991b**). Chemical studies on Mexican plants used in traditional medicine, XVIII. New secondary metabolites from *Dodonaea viscosa*. Journal of natural products, 54, 913−917.

Mata, R., Rojas, A., Acevedo, L., Estrada, S., Calzada, F., Rojas, I., Bye R., Linares, E. (**1997**). Smooth Muscle Relaxing Flavonoids and Terpenoids from *Conyza filaginoides*. Planta Medica, 63, 31–35.

Mata R., Macías M. L., Rojas I. S., Lotina-Hennsen B., Toscano R. A. Anaya A. L. (**1998**). Phytotoxic compounds from *Esenbeckia Yaxhoob*. Phytochemistry 49, 441−449.

Mata, R., Rivero-Cruz, I., Rivero-Cruz, B., Bye, R., Timmermann, B. N. (**2002**). Sesquiterpene lactones and phenylpropanoids from *Cosmos pringlei*. Journal of natural products, 1030−1032.

Morales-Sánchez, V., Rivero-Cruz, I., Laguna-Hernández, G., Salazar-Chávez, G., Mata, R. (**2014**). Chemical composition, potential toxicity, and quality control procedures of the crude drug of *Cyrtopodium macrobulbon*. Journal of ethnopharmacology, 154, 790−797.

Navarrete, A., Trejo-Miranda, J. L., Reyes-Trejo, L. (**2002**). Principles of root bark of *Hippocratea excelsa* (Hippocrataceae) with gastroprotective activity. Journal of Ethnopharmacology, 79, 383−388.

Pérez-Vásquez, A., Reyes, A., Linares, E., Bye, R., Mata, R. (**2005**). Phytotoxins from *Hofmeisteria schaffneri*: Isolation and Synthesis of 2‘-(2‘‘-Hydroxy-4‘‘-methylphenyl)-2‘-oxoethyl Acetate. Journal of Natural Products, 68, 959–962.

Pérez-Vásquez, A., Aguilar-Cruz, R., Bye, R., Linares, E., Rivero-Cruz, I. (**2020**). UHPLC-MS Analysis of Polyphenols in the Aqueous Extract of *Hydrangea seemannii*. Revista Brasileira de Farmacognosia, 30, 7−11.

Rivero-Cruz, F., García-Aguirre, G., Cerda-García-Rojas, C.M., Mata, R. Conformational behavior and absolute stereostructure of two phytotoxic nonenolides from the fungus *Phoma herbarum* (**2000**) Tetrahedron, 56, 5337-5344.

Reyes-Ramírez, A., Leyte-Lugo, M., Figueroa, M., Serrano-Alba, T., González-Andrade, M., Mata, R. (**2011**). Synthesis, biological evaluation, and docking studies of gigantol analogs as calmodulin inhibitors. European journal of medicinal chemistry, 46, 2699−2708.

Rivero-Cruz, I., Acevedo, L., Guerrero, J. A., Martínez, S., Pereda-Miranda, R., Mata, R., Bye., Franzblau S., Barbara N Timmermann, B. N. (**2005**). Antimycobacterial agents from selected Mexican medicinal plants. Journal of pharmacy and Pharmacology, 57, 1117−1126.

Rivero-Cruz, B., Rivero-Cruz, I., Rodríguez-Sotres, R., Mata, R. (**2007**). Effect of natural and synthetic benzyl benzoates on calmodulin. Phytochemistry, 68, 1147−1155.

Rojas, A., Hernandez, L., Pereda-Miranda, R., Mata, R. (**1992**). Screening for antimicrobial activity of crude drug extracts and pure natural products from Mexican medicinal plants. Journal of ethnopharmacology, 35, 275−283.

Rojas, S., Acevedo, L., Macías, M., Toscano, R. A., Bye, R., Timmermann, B., Mata, R. (**2003**). Calmodulin Inhibitors from *Leucophyllum ambiguum*. *Journal of Natural Products*, 66, 221–224.

Rodriguez Lizana, M. (**1998**). Triterpenoides de las especies *Maxillaria densa* Lindley (Orchidaceae) y *Scaphyglottis livida* (Lindley) Schitr. (Orchidaceae). Tesis de Pregrado. Universidad Nacional Autónoma de Mexico, Mexico. http://132.248.9.195/pdbis/263825/Index.html.

Valencia-Islas, N., Abbas, H., Bye, R., Toscano, R., Mata, R. (**2002**). Phytotoxic compounds from *Prionosciadium watsoni*. Journal of Natural Products 65 (6), 828−83.

Valencia-Islas, N. A., Paul, R. N., Shier, W. T., Mata, R., Abbas, H. K. (**2002**). Phytotoxicity and ultrastructural effects of gymnopusin from the orchid *Maxillaria densa* on duckweed (*Lemna pausicostata*) frond and root tissues. Phytochemistry, 61, 141−148.

Segura, R., Calderón, J., Toscano, R., Gutiérrez, A., Mata, R. (**1994**). Cedrelanolide I, a new limonoid from *Cedrela salvadorensis*. Tetrahedron letters, 35, 3427−3440.

Segura-Correa, R., Mata, R., Anaya, A. L., Hernandez-Bautista, B., Villena, R., Soriano-Garcia, M., Bye R., Linares, E. (**1993**). New tetranortriterpenoids from *Swietenia humilis*. Journal of Natural Products, 56, 1567−1574.

Salinas-Arellano E., Pérez-Vásquez A., Rivero-Cruz I., Torres-Colin R., González-Andrade M., Rangel-Grimaldo M., Mata R. (**2020**). Flavonoids and Terpenoids with PTP-1B Inhibitory Properties from the Infusion of Salvia amarissima Ortega. Molecules, 25 (15), 3530.

Table S2. pIC_{50} data of most active compounds with a value in order of magnitude as the control (UA).

No	Name	IC_{50} (µM)	pIC_{50} (M)	r^2, χ^2	Modelo	ΔA
**1**	Canophyllol	33.2 ± 0.003	4.48	0.997, 3.1	DoseResp	0.2
**2**	5-*O*-(*β*-D-Glucopyranosyl)-7-methoxy-3'-4'-dihydroxy-4-phenylcoumarin	40.4 ± 0.02	4.39	0.999, 27	DoseResp	0.3
**3**	3,4-Dimethoxy-2,5-phenanthrenediol	47.7 ± 0.1	4.32	0.975, 1	DoseResp	0.3
**4**	Masticadienonic acid	49.1 ± 0.004	4.31	0.993, 4.5	DoseResp	0.4
**5**	4',5,6-Trihydroxy-3',7-dimethoxyflavone	57.3 ± 0.02	4.24	0.991, 1.9	DoseResp	0.4
**6**	*E*/*Z*-vermelhotin	79.6 ± 0.1	4.10	0.997, 1.3	DoseResp	0.6
**7**	Tajixanthone hydrate	83.2 ± 0.001	4.08	0.999, 2.4	DoseResp	0.6
**8**	Quercetin-3-*O*-(6"-benzoyl)-*β*-D-galactoside	88.8 ± 0.05	4.05	0.998, 4.3	DoseResp	0.6
**9**	Lichexanthone	88.8 ± 0.005	4.05	0.997, 1.9	DoseResp	0.6
**10**	Melianodiol	92.1 ± 0.04	4.04	0.992, 3.5	Hill1	0.6
**11**	Confusarin	99.1 ± 0.001	4.00	0.999, 2.1	DoseResp	0.7
**14**	3-O-*β*-D-Glucopyranosyl-23,24-dihydrocucurbitacin F	358.3 ± 0.012	3.45	0.998, 4.7	DoseResp	1.2
**15**	Arvenin I	564.3 ± 0.041	3.25	0.999, 2.2	Hill1	1.4
**18**	6"-*O*-Acetyl-5-*O*-*β*-D-galactopyranosyl-7,4'-dihydroxy-4-phenylcoumarin	348.6 ± 0.03	3.46	0.999, 0.7	Hill1	1.2
**25**	Phenol, 3-[2-(1,3-benzodioxol-5-yl)ethyl]-]	662.1 ± 0.13	3.18	0.994, 12	DoseResp	1.5
**32**	Isoalloalantolactone	443.2 ± 0.05	3.35	0.999, 65	DoseResp	1.3
**34**	Batatasin III	247.4 ± 0.07	3.61	0.999, 3.8	DoseResp	1.1
**40**	Benzomalvin A	424.2 ± 0.005	3.37	0.999, 41	DoseResp	1.3
**41**	Gymnopusin	431.4 ± 0.01	3.37	0.994, 3.1	DoseResp	1.3
**43**	Eicosyl 4-hydroxy-3-methoxy-cinnamate	648.3 ± 0.13	3.19	0.995, 3.6	DoseResp	1.5
**46**	Calein A	234.5 ± 0.02	3.63	0.997, 0.9	DoseResp	1.0
**47**	2'-Methoxykobusin	125.3 ± 0.009	3.90	0.998, 1.2	Hill1	0.8
**49**	3α-Hydroxymasticadienonic acid	150.7 ± 0.005	3.82	0.996, 6.4	DoseResp	0.8
**50**	7-Methylluteolin	434.9 ± 0.008	3.36	0.998, 15	DoseResp	1.3
**51**	Trifolin	859 ± 0.3	3.07	0.996, 90	DoseResp	1.6
**53**	Pinocembrin	306.2 ± 0.02	3.51	0.999, 4.4	DoseResp	1.1

**54**	5,7-Diacetylchrysin	361.8 ± 0.04	3.44	0.996, 9.6	Hill1	1.2
**56**	Friedelin	887.8 ± 0.04	3.05	0.997, 4.8	DoseResp	1.6
**58**	3-*epi*-Oleanolic acid	108.6 ± 0.01	3.96	0.965, 70	DoseResp	0.7
**63**	8-*β*-D-glucopyranosyloxy-4-methoxy-5-methyl-coumarin	165.7 ± 0.02	3.78	0.998, 2.7	DoseResp	0.9
**64**	5*α*-lanosta-24,24-dimethyl-9(11),25-dien-3*β*-ol	398.5 ± 0.03	3.40	0.998, 1,8	DoseResp	1.3
**67**	6''-*O*-acetyl-5-*O*-*β*-D-galactopyranosyl-7,3',4'-trihydroxy-4-phenylcoumarin	254.3 ± 0.2	3.59	0.998, 0.4	DoseResp	1.1
**73**	5-*O*-*β*-D-Apiofuranosyl-(1→6)-*β*-D-glucopyranosyl-7-metoxi-3'-4'-dihydroxy-4-phenylcoumarin	358.6 ± 0.07	3.45	0.997, 19.2	DoseResp	1.2
**77**	4-Hydroxy-3,3',5-trimetoxybibencyl	221 ± 0.1	3.66	0.998, 5.4	Hill1	1.0
**82**	(-)-Epicatechin 3-*O*-gallate	142.4 ± 0.007	3.85	0.999, 2	Hill1	0.8
**84**	*α*-Spinasterol	989.1 ± 0.14	3.00	0.985, 0.1	DoseResp	1.7
**85**	*α*-Spinasterol acetate	277.2 ± 0.01	3.56	0.993, 0.1	DoseResp	1.1
**86**	Methyl ester of masticadienonic acid	324.5 ± 0.001	3.49	0.997, 60	DoseResp	1.2
**89**	Isomasticadienonic acid	419 ± 0.01	3.38	0.994, 1	DoseResp	1.3
**90**	21-*α*-*H*-Hopane acetate	883.9 ± 0.2	3.05	0.987, 1	Hill1	1.6
**91**	3,3',4',5-Tetramethoxybibenzyl	419.5 ± 0.006	3.38	0.996, 1	DoseResp	1.3
**93**	Xanthyletin	864.6 ± 0.15	3.06	0.988, 1	Hill1	1.6
**94**	(+)-5-Methoxyhamaudol	298.5 ± 0.15	3.53	0.97, 1	Hill1	1.1
**96**	Albolutein A	19.1 ± 1.4	4.72		Hill1	-0.1
**97**	Albolutein B	25.8 ± 25.8	4.59		Hill1	0.1
**98**	Albolutein C	129.5 ± 2.6	3.89		Hill1	0.8

**99**	*o*-Anthranilic acid	12.5 ± 0.003	4.90		Hill1	-0.2
**C**[2]	Ursolic acid	21.8 ± 0.004	4.66	0.996, 1	DoseResp	0.0

Table S3. Theoretical binding properties of compounds **2**, **4**, **7–11** with $PTP1B_{1\text{-}400}$.

	**ΔG_T (Kcal mol^{-1})**	**Ki_T (mM)**
**PTP1B-2**	-7.02	6.84
**PTP1B-4**	-6.51	16.24
**PTP1B-7**	-6.87	8.82
**PTP1B-8**	-7.19	5.13
**PTP1B-9**	-5.73	60.89
**PTP1B-10**	-6.88	8.68
**PTP1B-11**	-5.2	149.45
**PTP1B-UA**	-6.73	11.19

Table S4. Interactions of compounds **2**, **4**, **7–11** with $PTP1B\text{-}_{1\text{-}400}$.

**Complex**	**Interaction residues**
**PTP1B-2**	Arg79, Phe196, Aeg199, Glu200, Ser201, Gly202, Leu204, Ser205, Pro206, Leu233, Asp236, Phe280, Gln288
**PTP1B-4**	Phe7, Glu8, Val184, Pro185, Glu186, Pro188, Asp265, Arg268, Phe269, Leu272, Ile275
**PTP1B-7**	Met1, Ile275, Glu276, Ala278, Lys279, Met282, Gly283, Asp284, Ser286, Val287, Gln290
**PTP1B-8**	Ala77, Gln78, Arg79, Phe196, Arg199, Glu200, Leu204, Ser205, Pro206, Leu233, Asp236, Lys237, Phe280, Gln288
**PTP1B-9**	Lys279, Met282, Gly283, Asp284, Ser286, Val287, Gln290
**PTP1B-10**	Met1, Glu4, Phe7. Glu8, Ile275, Glu276, Ala278, Lys279, Met282, Gly286, Asp284, Val287
**PTP1B-11**	Arg79, Phe196, Arg199, Glu200, Leu233, Asp236, Phe280, Gln288
**PTP1B-UA**	Lys279, Met282, Gly283, Asp284, Ser286, Val287, Gln290

Table S5. Prediction of physicochemical properties of compounds **1–11**.

No.	MW ≤ 500g/mol	FCsp^3>0.25	#RB < 10	#HBA < 10	#HBD < 5	TPSA < 140Å	Log P < 5	2- < LogD < 5	Bioavailability Score (0.85)
1	442.72	0.97	1	2	1	37.3	6.57	4.39	0.55
2	462.4	0.32	5	11	6	179.28	0.31	0.261	0.17
3	270.28	0.12	2	4	2	58.92	2.92	3.285	0.55
4	454.68	0.8	5	3	1	54.37	6.38	5.077	0.85
5	330.29	0.12	3	7	3	109.36	2.15	2.206	0.55
6	217.22	0.17	1	3	1	55.4	1.2	0.728	0.85
7	440.49	0.4	4	7	4	120.36	3.07	2.787	0.55
8	568.48	0.21	7	13	7	216.58	1.16	1.56	0.17
9	286.28	0.19	2	5	1	68.9	2.81	3.003	0.55
10	488.7	0.9	3	5	3	86.99	4.34	4.014	0.55
11	300.31	0.18	3	5	2	68.15	2.93	3.172	0.55
UA*	456.7	0.9	1	3	2	57.53	5.93	5.267	0.85
T*	685.06	1	20	9	6	154.32	4.49	3.632	0.17
E*	559.51	0.19	6	3	1	74.77	7.69	5.065	0.85
M*	129.16	0.5	2	2	3	91.49	-0.89	-2.054	0.55

*Positive controls, UA (Ursolic acid), T (trodusquemine), E (ertiprotafib) and M (metformin).

Table S6. Medicinal Chemistry properties of compounds **1–11**.

Calculated using OSIRIS-DataWarrior, SwissADME and ADMETlab 2.0. The data shows the number of alerts in every single MC rule, fragment or type of compound.

No	Toxicophores	PAINS	Brenk	Lipinski	GoldenTriangle	Alerts (MC)
1	0	0	0	1	0	1
2	3	1	2	2	0	8
3	2	0	1	0	0	3
4	1	0	2	1	1	5
5	2	1	1	0	0	4
6	0	0	2	0	0	2
7	2	0	2	0	0	4
8	2	1	1	3	1	8
9	2	0	1	0	0	3
10	0	0	1	0	0	1
11	2	0	1	0	0	3
UA*	0	0	1	1	1	3
T*	0	0	1	2	1	4
E*	1	0	0	2	1	4
M*	1	0	2	0	1	4

*Positive controls, UA (Ursolic acid), T (trodusquemine), E (ertiprotafib) and M (metformin).

Table S7. Pharmacokinetic properties related to absorption and distribution of compounds **1–11**.

Calculated using SwissADME and ADMETlab 2.0.

	**ABSORPTION (A)**				**DISTRIBUTION (D)**			
**No.**	**GI absorption**	**BBB permeant**	**PGP sub**	**Caco-2 ≥ -5.15**	**PPB≤90**	**VD (0.04-20 L/g)**	**Fu ≥ 20 %**	**Alerts (AD)**
1	Low	No	No	-5.205	96.32%	1.345	2.05%	2
2	Low	No	No	-6.203	86.71%	0.733	12.70%	4
3	High	Yes	No	-4.908	96.09%	0.516	5.82%	2
4	Low	No	No	-5.073	96.77%	0.716	2.24%	3
5	High	No	No	-4.985	92.51%	0.689	14.23%	1
6	High	No	No	-4.622	69.95%	0.642	36.30%	1
7	High	No	Yes	-4.977	90.43%	0.922	7.00%	2
8	Low	No	No	-6.295	98.05%	0.639	5.63%	2
9	High	Yes	No	-4.883	89.01%	0.946	10.38%	3
10	High	No	Yes	-4.759	93.24%	1.434	6.36%	2
11	High	Yes	No	-4.959	88.67%	0.484	13.76%	3
UA*	Low	No	No	-5.396	97.44%	0.672	2.43%	3
T*	Low	No	No	-6.031	59.36%	0.505	36.76%	3
E*	Low	No	Yes	-4.972	102.46%	0.383	0.37%	3
M*	High	No	No	-6.259	5.83%	1.16	74.15%	2

*Positive controls, UA (Ursolic acid), T (trodusquemine), E (ertiprotafib) and M (metformin).

Table S8. Pharmacokinetic properties related to metabolism and excretion of compounds **1–11**.

Calculated using SwissADME and ADMETlab 2.0.

	METABOLISM (M)					EXCRETION (E)		
**No.**	**CYP1A2 inh**	**CYP2C19 inh**	**CYP2C9 inh**	**CYP2D6 inh**	**CYP3A4 inh**	**CL≥15 mL/min/kg**	**$T_{1/2}$ long ↓**	**Alerts (ME)**
1	No	No	No	No	No	19.382	0.06	0
2	No	No	No	No	No	9.802	0.78	2
3	Yes	Yes	Yes	Yes	Yes	10.186	0.57	7
4	No	No	Yes	No	No	5.916	2.00	2
5	Yes	No	Yes	Yes	Yes	5.568	0.88	6
6	Yes	No	No	No	No	1.814	0.55	3
7	No	No	No	No	Yes	3.828	0.13	2
8	No	No	No	No	No	5.702	0.90	2
9	Yes	No	Yes	Yes	Yes	3.78	0.36	6
10	No	No	No	No	No	10.243	0.15	1
11	Yes	Yes	Yes	Yes	Yes	9.842	0.74	7
UA*	No	No	No	No	No	3.538	0.07	1
T*	No	No	No	No	Yes	4.342	0.04	2
E*	No	No	No	Yes	Yes	2.11	0.03	3
M*	No	No	No	No	No	3.504	0.47	2

*Positive controls, UA (Ursolic acid), T (trodusquemine), E (ertiprotafib) and M (metformin).

Table S9. Toxicological properties of compounds **1–11** calculated using DataWarrior.

**TOXICITY (T)**					
**No.**	**Mutagenic**	**Tumorigenic**	**Teratogenic**	**Irritant**	**Alerts (T)**
1	none	none	none	none	0
2	none	none	high	none	1
3	low	low	none	none	2
4	none	none	none	none	0
5	high	none	none	none	1
6	none	none	none	none	0
7	high	none	low	none	2
8	none	none	none	high	1
9	high	none	high	none	2
10	none	none	none	none	0
11	low	low	none	none	2
UA*	none	none	none	none	0
T*	none	none	none	none	0
E*	none	high	none	none	1
M*	none	none	low	none	1

*Positive controls, UA (Ursolic acid), T (trodusquemine), E (ertiprotafib) and M (metformin).

Table S10. Estimated toxicity profile for selected molecules (**1**−**11**).

Softwires used: DataWarrior v.5.5.0 and ICM v. 3.9 (Molsoft). In addition, values of LD_{50} were estimated with the software T.E.S.T. v. 5.1 (EPA USA).

ID	TOX Score	TOX CLASS	MOL CACO2	MOLL D50	MOL PAINS	MOLPGP inhibitor	Mutagenic	Tumorigenic	Reproductive Effective	Irritant	DL50 (TEST)	Tox_Names
**1**	0.00	0.53	-4.74	2.19	0.07	0.99	none	none	none	none	2873.99	
**2**	1.17	0.33	-5.90	2.34	0.71	0.28	none	none	high	none	906.12	coumarin; quinone-like
**3**	2.12	0.82	-4.87	2.26	0.67	0.09	low	low	none	none	1074.47	Phenanthrene and phenanthrene containing compounds; Phenanthrenes; polycyclic; resorcinol;α-Naphthols
**4**	1.79	0.53	-4.97	2.06	0.06	0.90	none	none	none	none	567.13	Carboxylic acids; Michael acceptor
**5**	1.17	0.41	-5.26	1.82	0.52	0.56	high	none	none	none	247.80	quinone-like; resorcinol
**6**	0.29	0.42	-4.85	1.59	0.15	0.01	none	none	none	none	1611.74	thione
**7**	0.00	0.37	-5.47	1.44	0.35	0.01	high	none	low	none	381.27	
**8**	1.21	0.44	-5.90	1.49	0.91	0.14	none	none	none	none	886.13	quinone-like;resorcinol
**9**	0.32	0.49	-4.76	1.89	0.24	0.26	high	none	high	none	45.31	resorcinol
**10**	0.00	0.53	-5.06	2.03	0.10	0.14	none	none	none	none	171.41	
**11**	1.30	0.87	-4.97	1.46	0.53	0.13	low	low	none	none	881.76	Phenanthrene and phenanthrene containing compounds; Phenanthrenes; polycyclic

Table S11. Summary of alerts ADMET for the most active compounds (**1–11**).

Medicinal chemistry includes the Lipinski and Golden triangle rules. Regarding ADMET, we examine a molecule's properties to be a potential oral drug.

ALERTS				
**No**	**MC**	**AD**	**MET**	**TOTAL**
1	1	2	0	3
2	8	4	3	15
3	3	2	9	14
4	5	3	2	10
5	4	1	7	12
6	2	1	3	6
7	4	2	4	10
8	8	2	3	13
9	3	3	8	14
10	1	2	1	4
11	3	3	9	15
UA*	3	3	1	7
T*	4	3	2	9
E*	4	3	4	11
M*	4	2	3	9

[1]Positive controls, UA (Ursolic acid), T (trodusquemine), E (ertiprotafib) and M (metformin).

[2]Medicinal Chemistry: toxicophores groups, Brenk alerts, Lipinski, and Golden Triangle.

[3]ADMET: GI absorption, BBB permeant, Pgp substrate, Caco-2 permeability, PPB ≤ 90 %, VD, Fu; inhibition of CYP's isomorphs, $T_{1/2}$), mutagenic, tumorigenic, irritant, and reproductive effective.

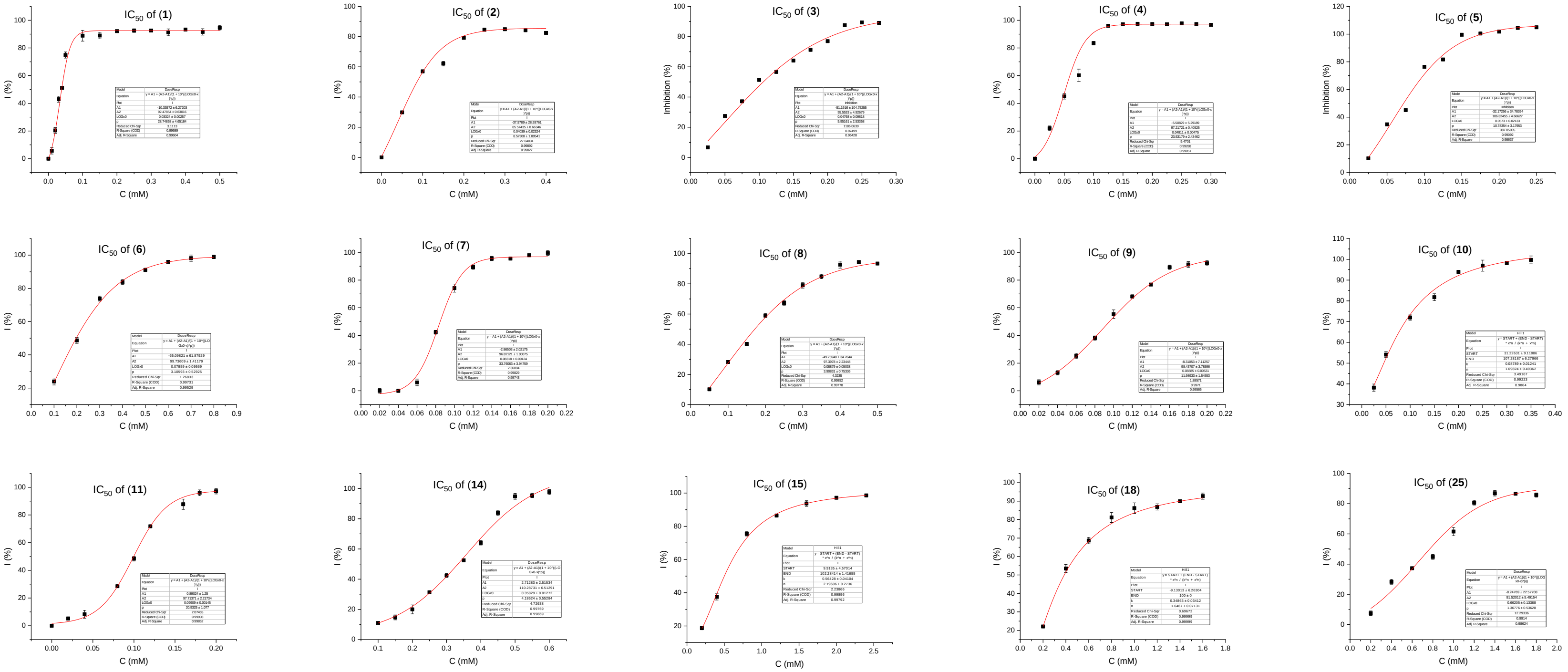

Figure S1. IC_{50} curves for the NP in Table 2, fit using Origin 8.0 software.

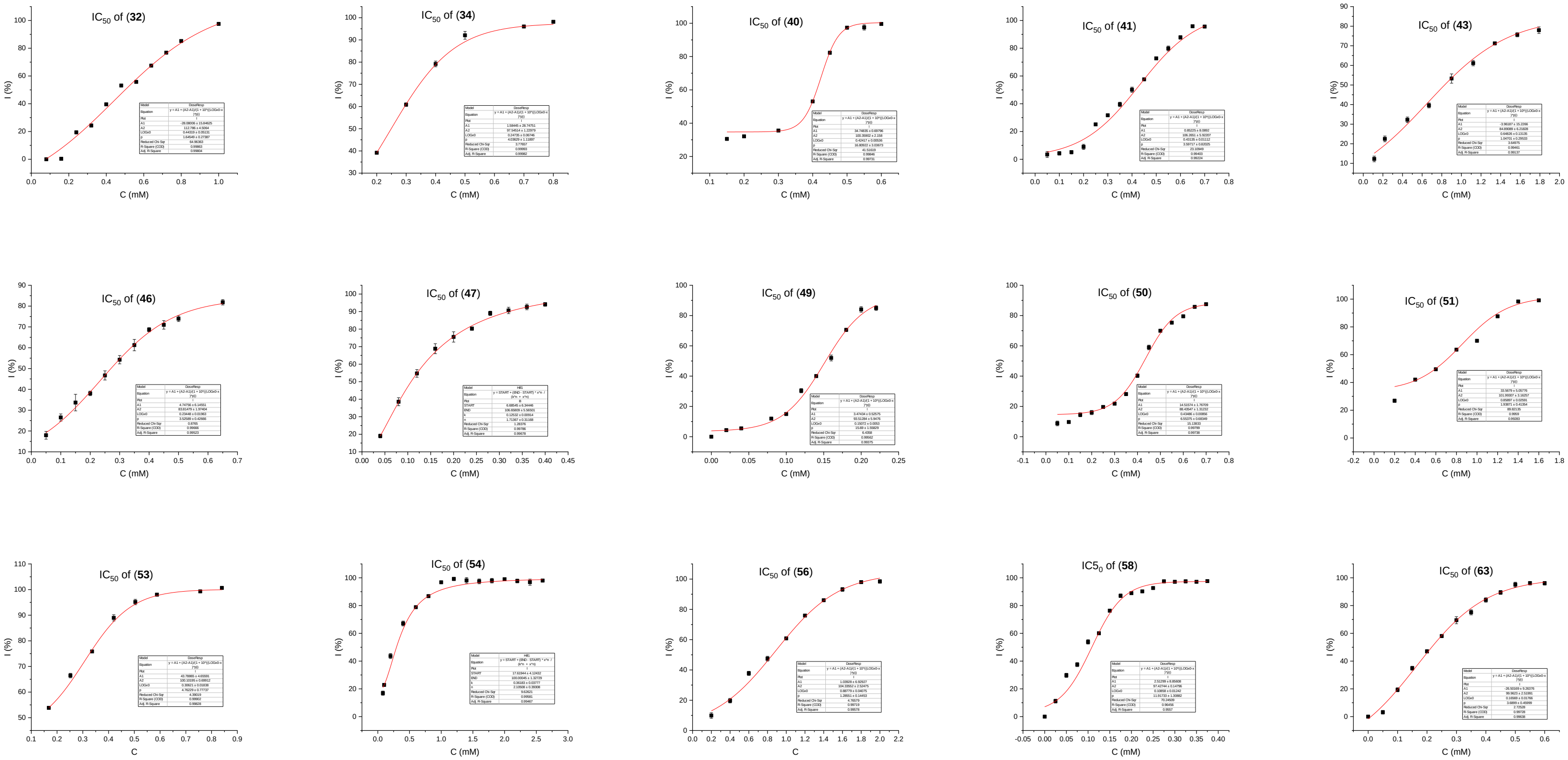

Figure S1. IC_{50} curves for the NP in Table 2, fit using Origin 8.0 software (cont.).

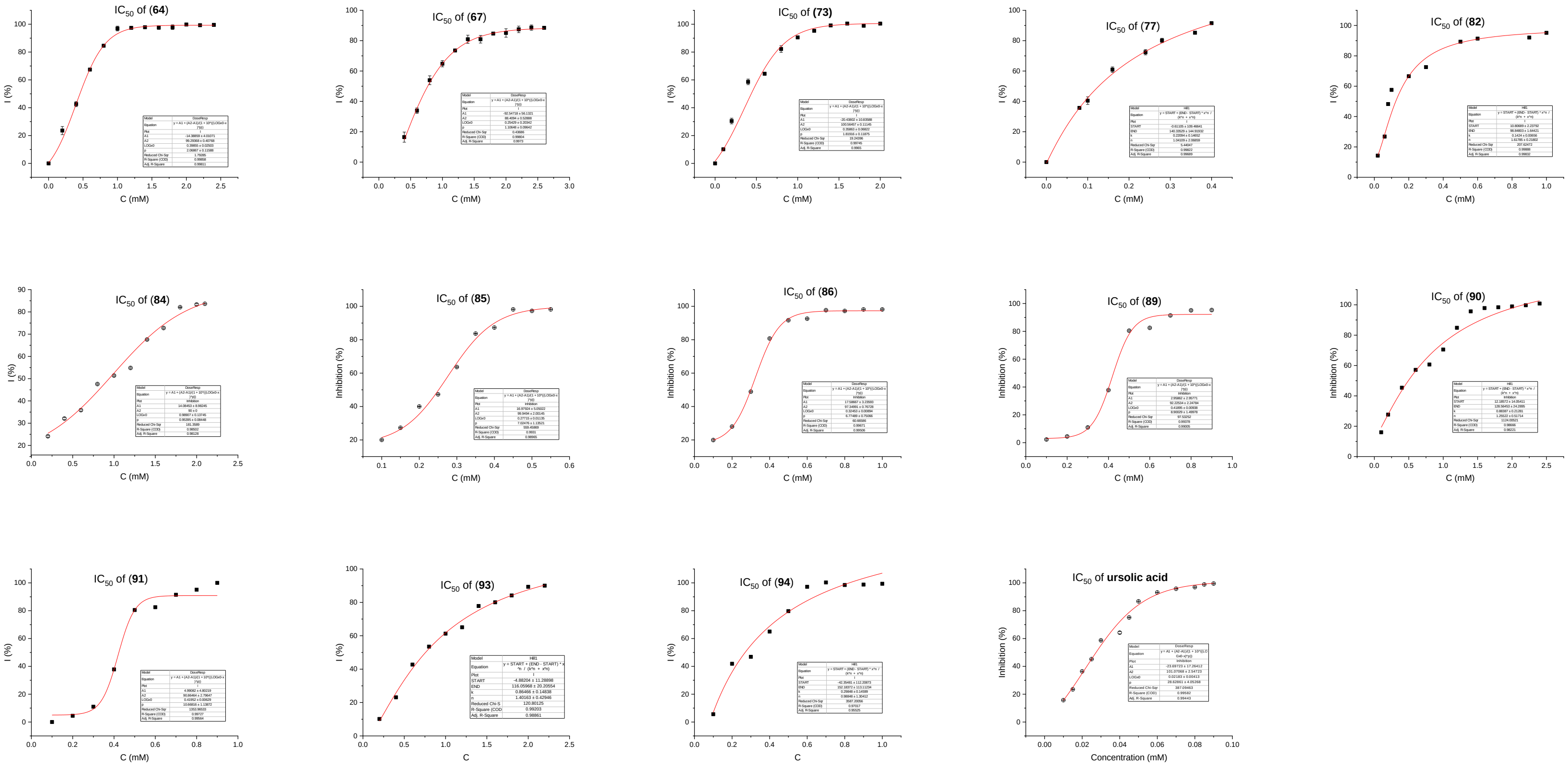

Figure S1. IC_{50} curves for the NP in Table 2, fit using Origin 8.0 software (cont.).

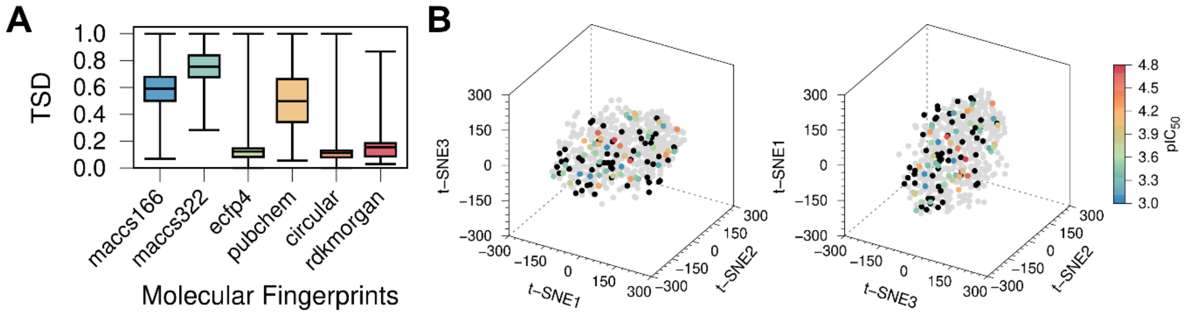

Figure S2. Tanimoto similarity cumulative distribution (TSD) plots.

(**A**) Molecular fingerprint selected for the chemical space between BIOFAQUIM and in-house library. (**B)** Projection of chemical space using t-SNE3.

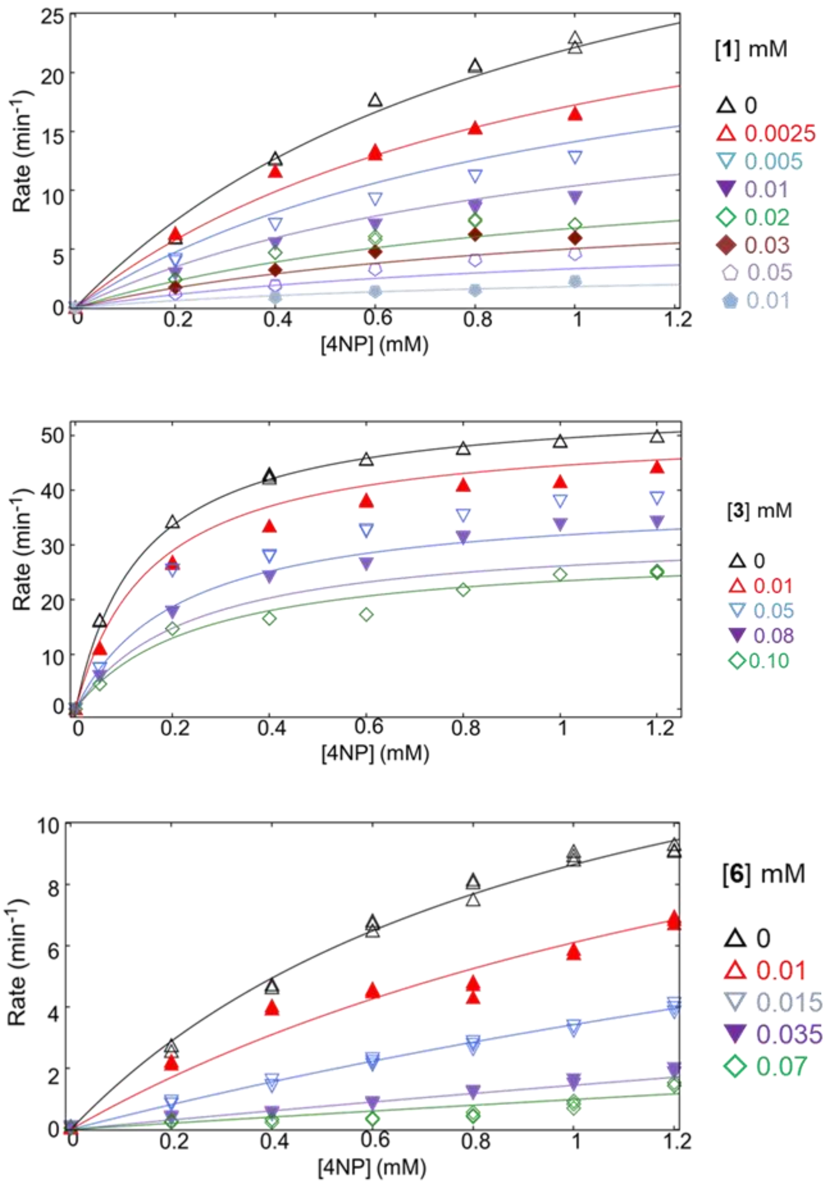

Figure S3. Michaelis-Menten curves.

Initial velocity as a function of substrate concentration at several fixed concentrations of the inhibitors **1**, **3**, and **6**.

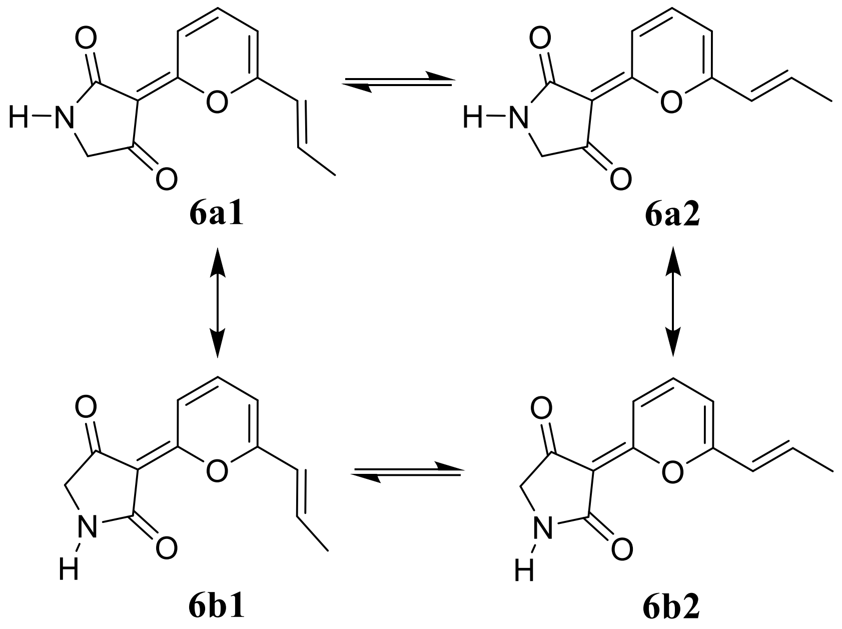

Figure S4. Equilibrium *E*/*Z* mixture.

Diastereoisomeric mixture (6.4:3.6, *E*/*Z* ratio) of **6a** and **6b**.

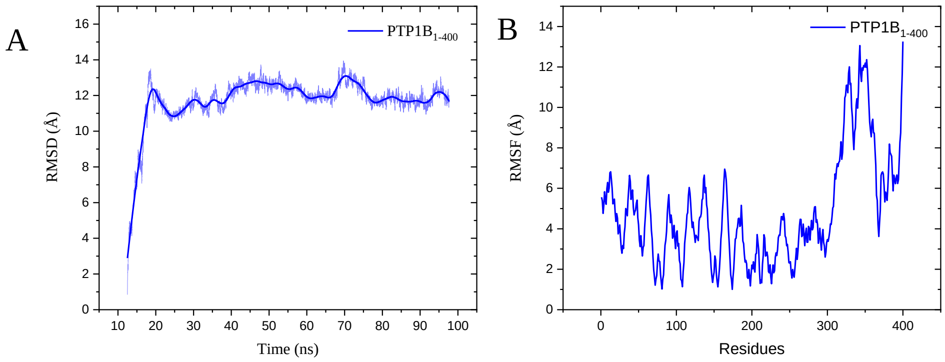

Figure S5. RMSD and RMSF of 100ns of molecular dynamics simulation of the structural model obtained from Alphafold of the PTP1B$_{1\text{-}400}$.

We can observe that after 30 ns the global conformational flexibility (**A**) remains constant. Regarding the RMSF (**B**), it is observed that the unstructured area of the protein (300 to 400 residues) is the one that presents the greatest movement.

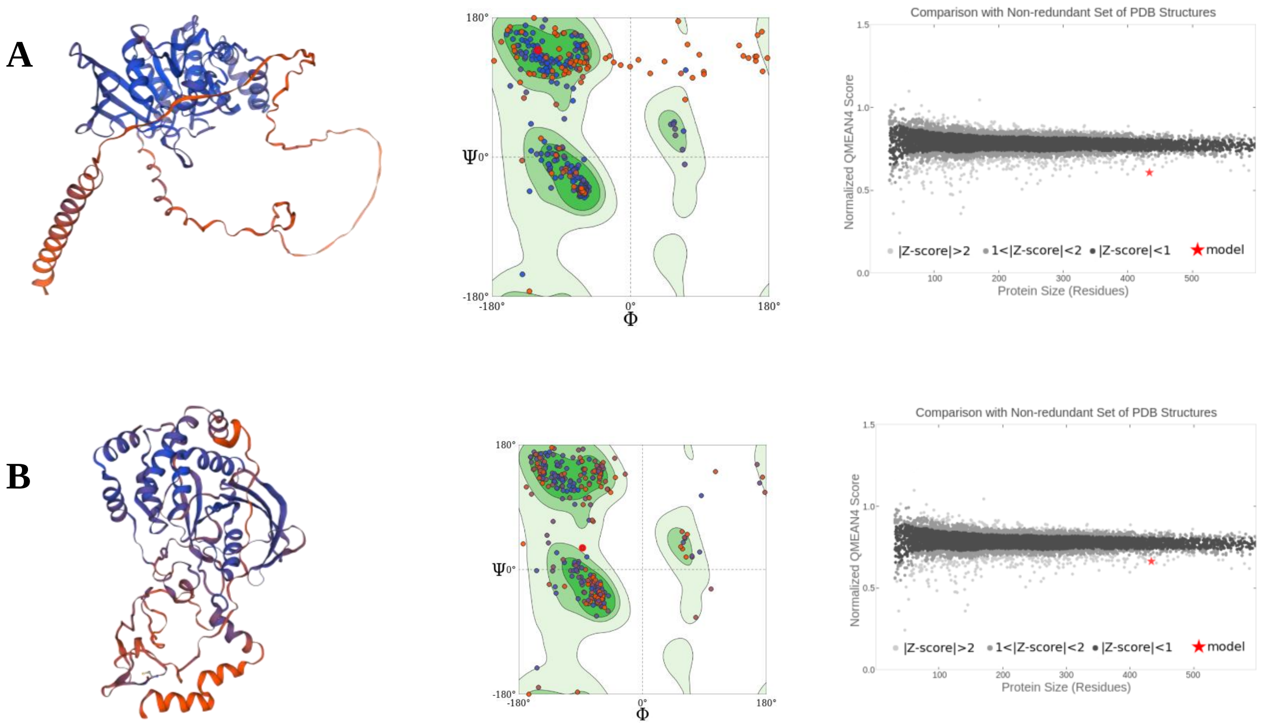

Figure S6. Structural model, Ramachandran plot and quality score of PTP1B$_{1\text{-}400}$ before (**A**) and after 100 ns of molecular dynamics simulation (**B**).

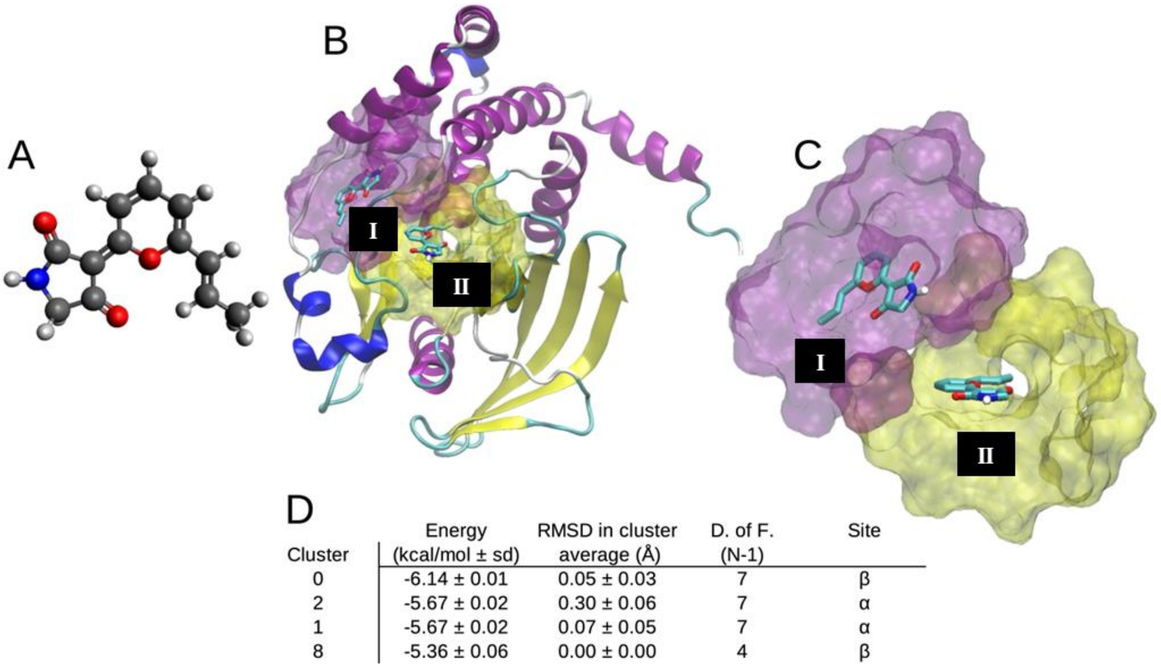

D

Cluster	Energy (kcal/mol ± sd)	RMSD in cluster average (Å)	D. of F. (N-1)	Site
0	-6.14 ± 0.01	0.05 ± 0.03	7	β
2	-5.67 ± 0.02	0.30 ± 0.06	7	α
1	-5.67 ± 0.02	0.07 ± 0.05	7	α
8	-5.36 ± 0.06	0.00 ± 0.00	4	β

Figure S7. Vermelhotin **6a1** docking into PTP1B model from AlphaFold 2.0.

(**A**) Three-dimensional model of **6a1**. (**B**) PTP1B residues (1–300) are shown as cartoons colored by secondary structure (**B**) where two binding pockets are identified as magenta (I) and yellow (II) translucent surfaces. The structure of the lowest energy cluster for sites I (cluster 2) and II (cluster 0) are shown superimposed as licorice. (**C**) The structure of the second lowest energy clusters for sites I (cluster 1) and II (cluster 8) are shown superimposed. (**D**) The average energies and RMSD (within each cluster) for the 0 lowest energy clusters are shown.

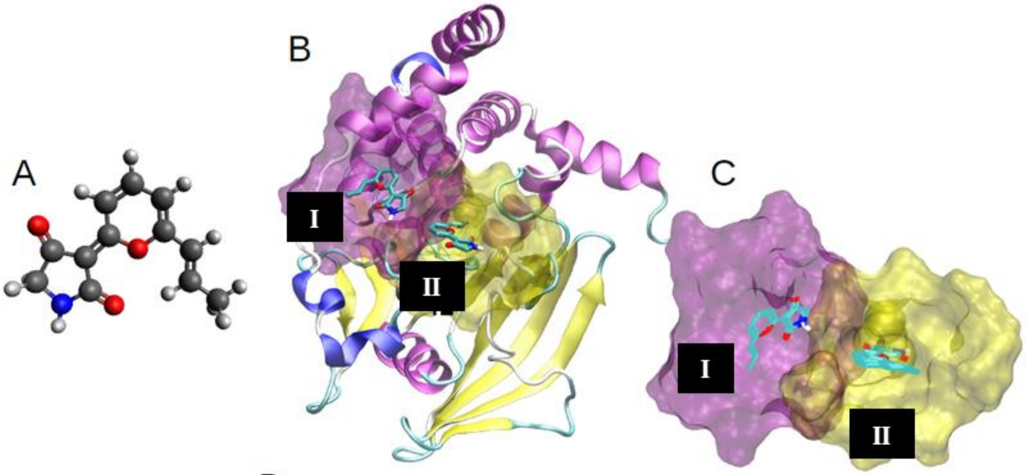

D

Cluster	Energy (kcal/mol ± sd)	RMSD in cluster average (Å)	D. of F. (N-1)	Site
0	-6.02 ± 0.02	0.08 ± 0.02	7	β
8	-5.99 ± 0.04	0.43 ± 0.23	5	β
1	-5.86 ± 0.01	0.03 ± 0.03	7	α
5	-5.64 ± 0.01	0.07 ± 0.02	6	α

Figure S8. Vermelhotin **6a2** docking into PTP1B model from AlphaFold 2.0.

(**A**) Three-dimensional model of **6a2**. (**B**) PTP1B (1–300) are shown as cartoons colored by secondary structure, where two binding pockets are identified as magenta (I) and yellow (II) translucent surfaces. The structure of the lowest energy cluster for sites I (cluster 1) and II (cluster 0) are shown superimposed as licorice. (**C**) The structure of the second lowest energy clusters for sites I (cluster 5) is shown superimposed, and II (cluster 8) are shown superimposed (**D**) The average energies and RMSD (within each cluster) for the 0 lowest energy clusters are shown.

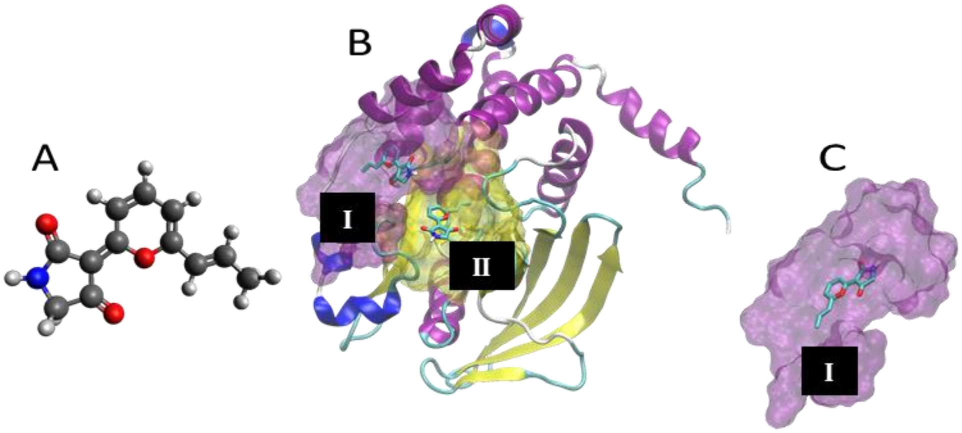

D

Cluster	Energy (kcal/mol ± sd)	RMSD in cluster average (Å)	D. of F. (N-1)	Site
3	-6.70 ± 0.01	0.07 ± 0.04	6	β
0	-5.95 ± 0.03	0.37 ± 0.03	7	α
7	-5.62 ± 0.02	0.08 ± 0.04	4	α

Figure S9. Vermelhotin **6b1** docking into PTP1B model from AlphaFold 2.0.

(**A**) Three-dimensional model of **6b1**. (**B**) PTP1B (1–300) are shown as cartoons colored by secondary structure, where two binding pockets are identified as magenta (I) and yellow (II) translucent surfaces. The structure of the lowest energy cluster for sites I (cluster 0) and II (cluster 3) are shown superimposed as licorice. (**C**) The structure of the second lowest energy clusters for sites I (cluster 7) is shown superimposed, only one cluster was found for site II. (**D**) The average energies and RMSD (within each cluster) for the 3 lowest energy clusters are shown.

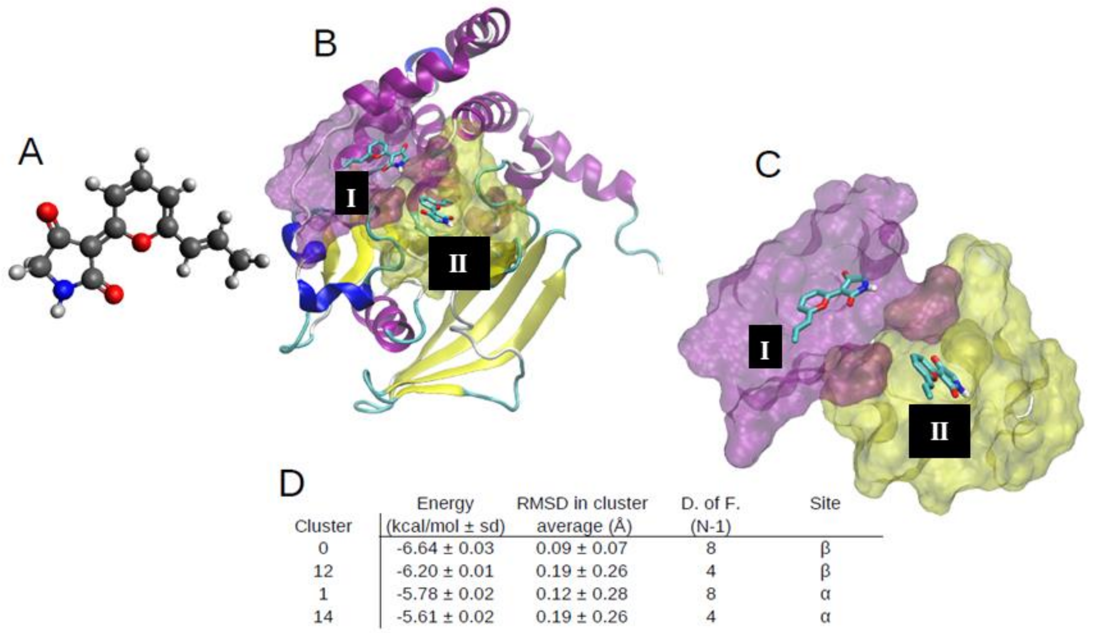

D

Cluster	Energy (kcal/mol ± sd)	RMSD in cluster average (Å)	D. of F. (N-1)	Site
0	-6.64 ± 0.03	0.09 ± 0.07	8	β
12	-6.20 ± 0.01	0.19 ± 0.26	4	β
1	-5.78 ± 0.02	0.12 ± 0.28	8	α
14	-5.61 ± 0.02	0.19 ± 0.26	4	α

Figure S10. Vermelhotin **6b2** docking into PTP1B model from AlphaFold 2.0.

(**A**) Three-dimensional model of **6b2**. (**B**) PTP1B (1–300) are shown as cartoons colored by secondary structure, where two binding pockets are identified as magenta (α) and yellow (β) translucent surfaces. The structure of the lowest energy cluster for sites α (cluster 0) and β (cluster 3) are shown superimposed as licorice. (**C**) The structure of the second lowest energy clusters for sites α (cluster 7) is shown superimposed, only one cluster was found for site β. (**D**) The average energies and RMSD (within each cluster) for the 3 lowest energy clusters are shown

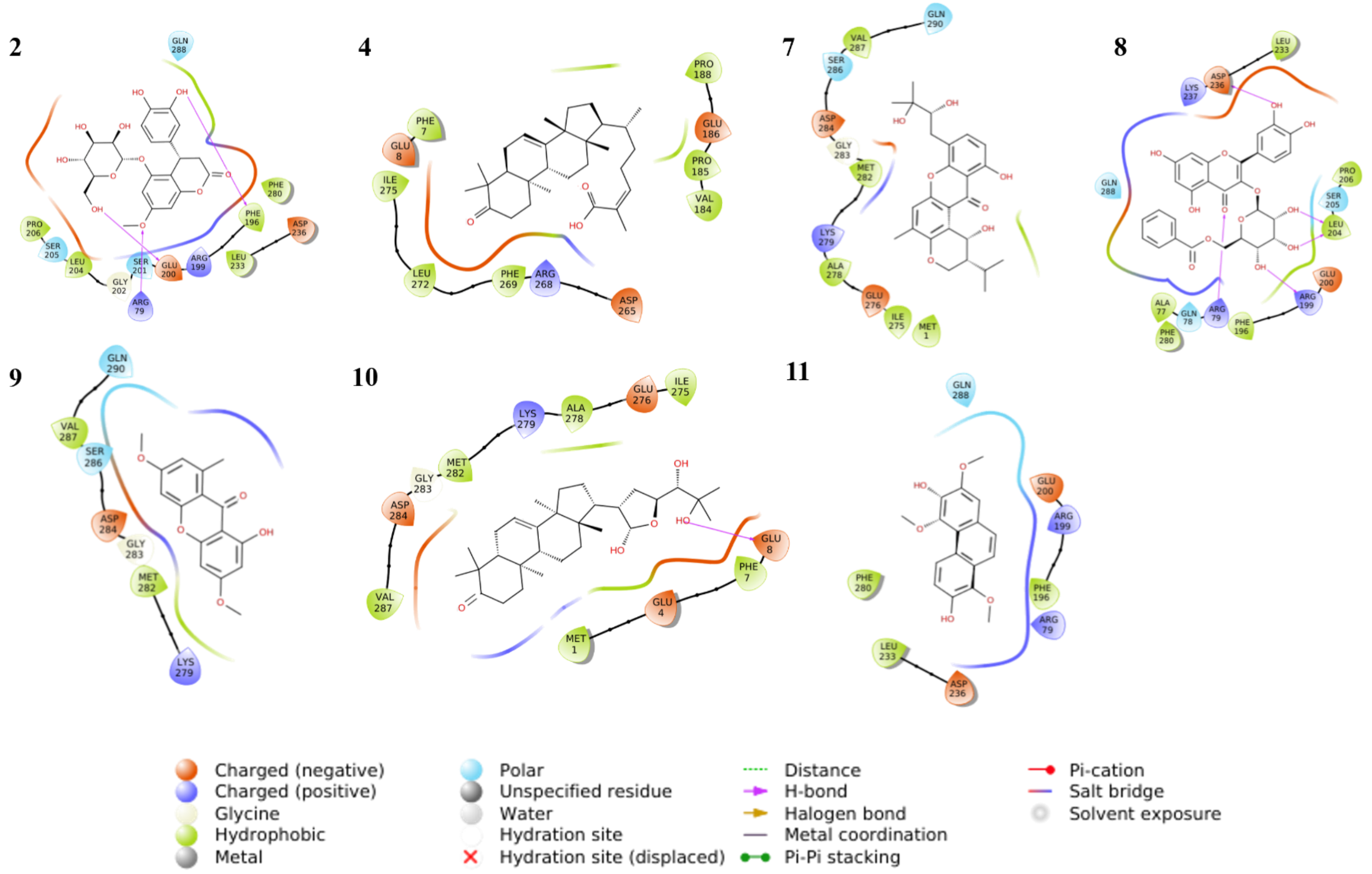

Figure S11. Interactions prediction between PTP1B-ligand complexes in 2D for compounds **2**, **4** and **7–11** (AlphaFold code: Q9PT91).

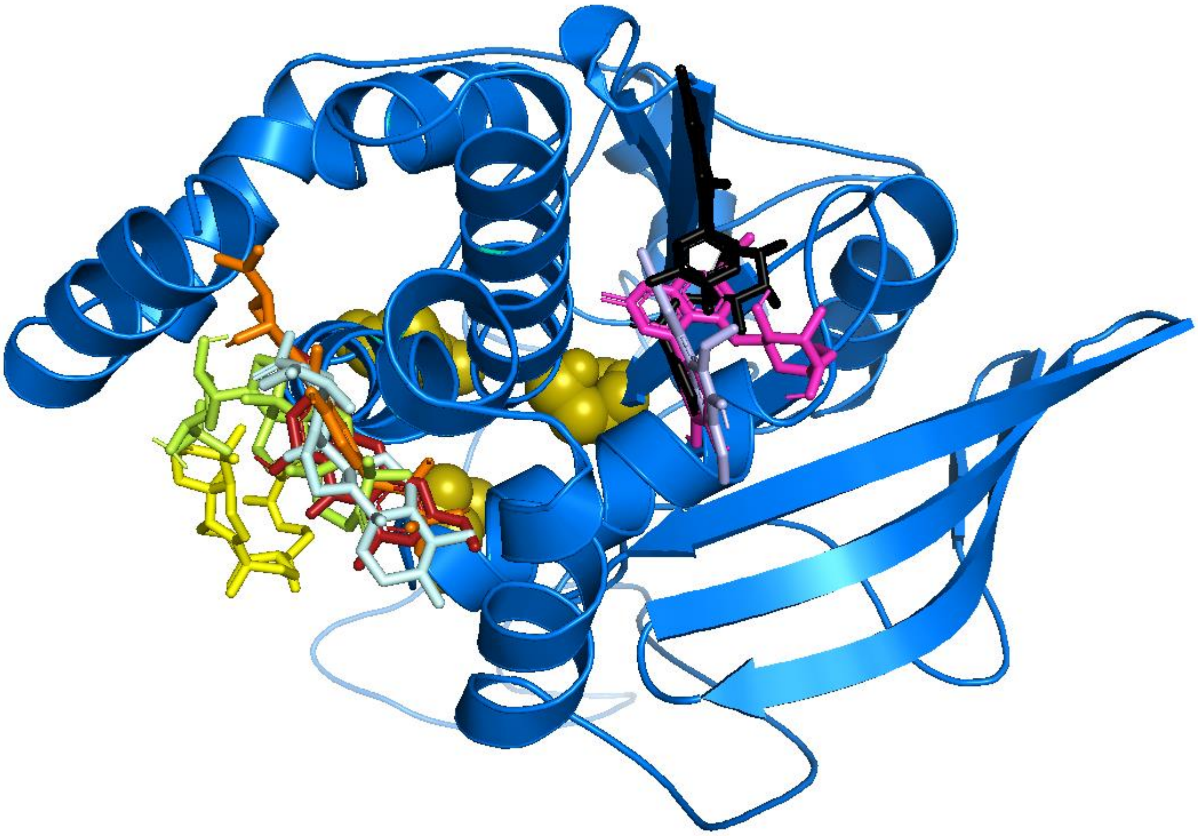

Figure S12. Interactions predictions the between PTP1B-ligand complexes for compounds **4**, **2**, and **7–11**.

In the center, the protein is displayed in blue (cartoon), active site in olive spheres, compounds are in sticks: light magenta (**2**), yellow (**4**), orange (**7**), black (**8**), firebrick (**9**), limon (**10**), violet (**11**), and UA (white)

(1)

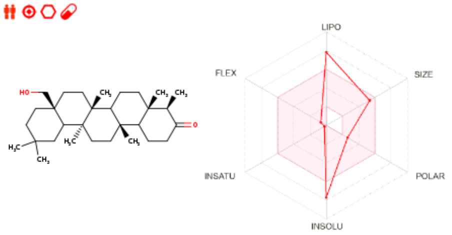

SMILES OC[C@@]12CCC(CC2[C@]2([C@@](CC1)(C)C1CC[C@]3(C([C@@]1(CC2)C)CCC(=O)[C@@H]3C)C)C)(C)C

Physicochemical Properties	
Formula	C30H50O2
Molecular weight	442.72 g/mol
Num. heavy atoms	32
Num. arom. heavy atoms	0
Fraction Csp3	0.97
Num. rotatable bonds	1
Num. H-bond acceptors	2
Num. H-bond donors	1
Molar Refractivity	135.56
TPSA	37.30 Å²
**Lipophilicity**	
Log $P_{o/w}$ (iLOGP)	4.29
Log $P_{o/w}$ (XLOGP3)	8.21
Log $P_{o/w}$ (WLOGP)	7.43
Log $P_{o/w}$ (MLOGP)	6.00
Log $P_{o/w}$ (SILICOS-IT)	6.91
Consensus Log $P_{o/w}$	6.57

Water Solubility	
Log *S* (ESOL)	-7.69
Solubility	9.02e-06 mg/ml ; 2.04e-08 mol/l
Class	Poorly soluble
Log *S* (Ali)	-8.85
Solubility	6.20e-07 mg/ml ; 1.40e-09 mol/l
Class	Poorly soluble
Log *S* (SILICOS-IT)	-7.30
Solubility	2.20e-05 mg/ml ; 4.97e-08 mol/l
Class	Poorly soluble
**Pharmacokinetics**	
GI absorption	Low
BBB permeant	No
P-gp substrate	No
CYP1A2 inhibitor	No
CYP2C19 inhibitor	No
CYP2C9 inhibitor	No
CYP2D6 inhibitor	No
CYP3A4 inhibitor	No
Log K_p (skin permeation)	-3.17 cm/s
**Druglikeness**	
Lipinski	Yes; 1 violation: MLOGP>4.15
Ghose	No; 3 violations: WLOGP>5.6, MR>130, #atoms>70
Veber	Yes
Egan	No; 1 violation: WLOGP>5.88
Muegge	No; 1 violation: XLOGP3>5
Bioavailability Score	0.55
**Medicinal Chemistry**	
PAINS	0 alert
Brenk	0 alert
Leadlikeness	No; 2 violations: MW>350, XLOGP3>3.[illegible]
Synthetic accessibility	5.14

(2)

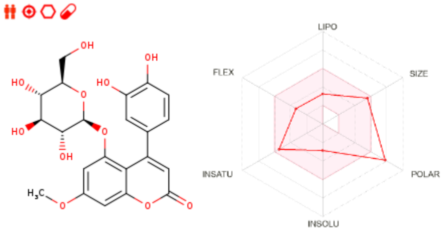

SMILES OC[C@H]1O[C@@H](Oc2cc(OC)cc3c2cc(cc(=O)o3)c2ccc(c(c2)O)O)[C@@H]([C@H]([C@@H]1O)O)O

Physicochemical Properties	
Formula	C22H22O11
Molecular weight	462.40 g/mol
Num. heavy atoms	33
Num. arom. heavy atoms	16
Fraction Csp3	0.32
Num. rotatable bonds	5
Num. H-bond acceptors	11
Num. H-bond donors	6
Molar Refractivity	112.60
TPSA	179.28 Å²
**Lipophilicity**	
Log $P_{o/w}$ (iLOGP)	1.97
Log $P_{o/w}$ (XLOGP3)	0.19
Log $P_{o/w}$ (WLOGP)	0.06
Log $P_{o/w}$ (MLOGP)	-1.07
Log $P_{o/w}$ (SILICOS-IT)	0.42
Consensus Log $P_{o/w}$	0.31

Water Solubility	
Log *S* (ESOL)	-2.86
Solubility	6.45e-01 mg/ml ; 1.40e-03 mol/l
Class	Soluble
Log *S* (Ali)	-3.51
Solubility	1.42e-01 mg/ml ; 3.07e-04 mol/l
Class	Soluble
Log *S* (SILICOS-IT)	-2.79
Solubility	7.48e-01 mg/ml ; 1.62e-03 mol/l
Class	Soluble
**Pharmacokinetics**	
GI absorption	Low
BBB permeant	No
P-gp substrate	No
CYP1A2 inhibitor	No
CYP2C19 inhibitor	No
CYP2C9 inhibitor	No
CYP2D6 inhibitor	No
CYP3A4 inhibitor	No
Log K_p (skin permeation)	-8.99 cm/s
**Druglikeness**	
Lipinski	No; 2 violations: NorO>10, NHorOH>5
Ghose	Yes
Veber	No; 1 violation: TPSA>140
Egan	No; 1 violation: TPSA>131.6
Muegge	No; 3 violations: TPSA>150, H-acc>10, H[illegible] don>5
Bioavailability Score	0.17
**Medicinal Chemistry**	
PAINS	1 alert: catechol_A
Brenk	2 alerts: catechol, cumarine
Leadlikeness	No; 1 violation: MW>350
Synthetic accessibility	5.31

Figure S13. SwissADME prediction for compounds **1–11** and positive controls.

(3)

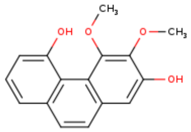

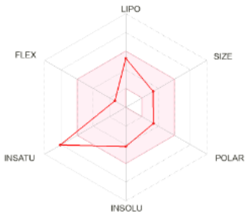

SMILES COc1c(OC)c(O)cc2c1c1c(O)cccc1cc2

Physicochemical Properties	
Formula	C16H14O4
Molecular weight	270.28 g/mol
Num. heavy atoms	20
Num. arom. heavy atoms	14
Fraction Csp3	0.12
Num. rotatable bonds	2
Num. H-bond acceptors	4
Num. H-bond donors	2
Molar Refractivity	78.48
TPSA	58.92 Å²
**Lipophilicity**	
Log $P_{o/w}$ (iLOGP)	2.37
Log $P_{o/w}$ (XLOGP3)	3.65
Log $P_{o/w}$ (WLOGP)	3.42
Log $P_{o/w}$ (MLOGP)	2.10
Log $P_{o/w}$ (SILICOS-IT)	3.06
Consensus Log $P_{o/w}$	2.92

Water Solubility	
Log *S* (ESOL)	-4.20
Solubility	1.70e-02 mg/ml ; 6.29e-05 mol/l
Class	Moderately soluble
Log *S* (Ali)	-4.58
Solubility	7.17e-03 mg/ml ; 2.65e-05 mol/l
Class	Moderately soluble
Log *S* (SILICOS-IT)	-4.86
Solubility	3.77e-03 mg/ml ; 1.40e-05 mol/l
Class	Moderately soluble
**Pharmacokinetics**	
GI absorption	High
BBB permeant	Yes
P-gp substrate	No
CYP1A2 inhibitor	Yes
CYP2C19 inhibitor	Yes
CYP2C9 inhibitor	Yes
CYP2D6 inhibitor	Yes
CYP3A4 inhibitor	Yes
Log K_p (skin permeation)	-5.36 cm/s
**Druglikeness**	
Lipinski	Yes; 0 violation
Ghose	Yes
Veber	Yes
Egan	Yes
Muegge	Yes
Bioavailability Score	0.55
**Medicinal Chemistry**	
PAINS	0 alert
Brenk	1 alert: polycyclic_aromatic_hydrocarbon_
Leadlikeness	No; 1 violation: XLOGP3>3.5
Synthetic accessibility	1.98

(4)

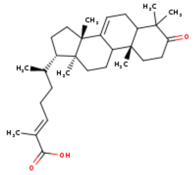

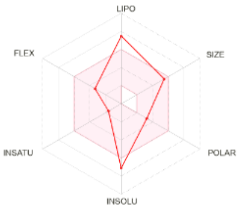

SMILES C[C@@H]([C@@H]1CC[C@]2([C@@]1(C)CCC1C2=CCC2[C@]1(C)CCC(=O)C2(C)C)C)CC/C=C(/C(=O)O)\C

Physicochemical Properties	
Formula	C30H46O3
Molecular weight	454.68 g/mol
Num. heavy atoms	33
Num. arom. heavy atoms	0
Fraction Csp3	0.80
Num. rotatable bonds	5
Num. H-bond acceptors	3
Num. H-bond donors	1
Molar Refractivity	137.85
TPSA	54.37 Å²
**Lipophilicity**	
Log $P_{o/w}$ (iLOGP)	4.11
Log $P_{o/w}$ (XLOGP3)	7.59
Log $P_{o/w}$ (WLOGP)	7.61
Log $P_{o/w}$ (MLOGP)	5.63
Log $P_{o/w}$ (SILICOS-IT)	6.93
Consensus Log $P_{o/w}$	6.38

Water Solubility	
Log *S* (ESOL)	-7.11
Solubility	3.52e-05 mg/ml ; 7.75e-08 mol/l
Class	Poorly soluble
Log *S* (Ali)	-8.57
Solubility	1.23e-06 mg/ml ; 2.70e-09 mol/l
Class	Poorly soluble
Log *S* (SILICOS-IT)	-6.40
Solubility	1.81e-04 mg/ml ; 3.97e-07 mol/l
Class	Poorly soluble
**Pharmacokinetics**	
GI absorption	Low
BBB permeant	No
P-gp substrate	No
CYP1A2 inhibitor	No
CYP2C19 inhibitor	No
CYP2C9 inhibitor	Yes
CYP2D6 inhibitor	No
CYP3A4 inhibitor	No
Log K_p (skin permeation)	-3.68 cm/s
**Druglikeness**	
Lipinski	Yes; 1 violation: MLOGP>4.15
Ghose	No; 3 violations: WLOGP>5.6, MR>130, #atoms>70
Veber	Yes
Egan	No; 1 violation: WLOGP>5.88
Muegge	No; 1 violation: XLOGP3>5
Bioavailability Score	0.85
**Medicinal Chemistry**	
PAINS	0 alert
Brenk	2 alerts: isolated_alkene, michael_accep
Leadlikeness	No; 2 violations: MW>350, XLOGP3>3.5
Synthetic accessibility	5.97

Figure S13. SwissADME prediction for compounds **1–11** and positive controls (cont.).

(5)

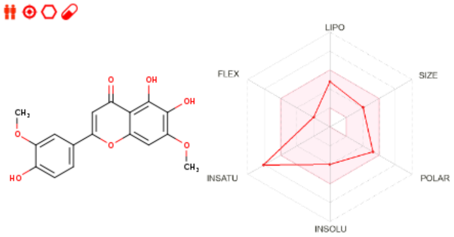

SMILES COc1cc(ccc1O)c1cc(=O)c2c(o1)cc(c(c2O)O)OC

Physicochemical Properties	
Formula	C17H14O7
Molecular weight	330.29 g/mol
Num. heavy atoms	24
Num. arom. heavy atoms	16
Fraction Csp3	0.12
Num. rotatable bonds	3
Num. H-bond acceptors	7
Num. H-bond donors	3
Molar Refractivity	86.97
TPSA	109.36 Å²

Lipophilicity	
Log $P_{o/w}$ (iLOGP)	2.77
Log $P_{o/w}$ (XLOGP3)	2.83
Log $P_{o/w}$ (WLOGP)	2.59
Log $P_{o/w}$ (MLOGP)	-0.07
Log $P_{o/w}$ (SILICOS-IT)	2.59
Consensus Log $P_{o/w}$	2.15

Water Solubility	
Log *S* (ESOL)	-3.97
Solubility	3.57e-02 mg/ml ; 1.08e-04 mol/l
Class	Soluble
Log *S* (Ali)	-4.78
Solubility	5.43e-03 mg/ml ; 1.64e-05 mol/l
Class	Moderately soluble
Log *S* (SILICOS-IT)	-4.63
Solubility	7.71e-03 mg/ml ; 2.33e-05 mol/l
Class	Moderately soluble

Pharmacokinetics	
GI absorption	High
BBB permeant	No
P-gp substrate	No
CYP1A2 inhibitor	Yes
CYP2C19 inhibitor	No
CYP2C9 inhibitor	Yes
CYP2D6 inhibitor	Yes
CYP3A4 inhibitor	Yes
Log K_p (skin permeation)	-6.31 cm/s

Druglikeness	
Lipinski	Yes; 0 violation
Ghose	Yes
Veber	Yes
Egan	Yes
Muegge	Yes
Bioavailability Score	0.55

Medicinal Chemistry	
PAINS	1 alert: catechol_A
Brenk	1 alert: catechol
Leadlikeness	Yes
Synthetic accessibility	3.29

(6)

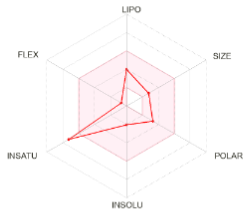

SMILES C/C=C/C1=CC=C/C(=C/2\C(=O)CNC2=O)/O1

Physicochemical Properties	
Formula	C12H11NO3
Molecular weight	217.22 g/mol
Num. heavy atoms	16
Num. arom. heavy atoms	0
Fraction Csp3	0.17
Num. rotatable bonds	1
Num. H-bond acceptors	3
Num. H-bond donors	1
Molar Refractivity	61.88
TPSA	55.40 Å²

Lipophilicity	
Log $P_{o/w}$ (iLOGP)	1.90
Log $P_{o/w}$ (XLOGP3)	1.49
Log $P_{o/w}$ (WLOGP)	0.60
Log $P_{o/w}$ (MLOGP)	0.16
Log $P_{o/w}$ (SILICOS-IT)	1.84
Consensus Log $P_{o/w}$	1.20

Water Solubility	
Log *S* (ESOL)	-2.06
Solubility	1.89e+00 mg/ml ; 8.72e-03 mol/l
Class	Soluble
Log *S* (Ali)	-2.26
Solubility	1.19e+00 mg/ml ; 5.49e-03 mol/l
Class	Soluble
Log *S* (SILICOS-IT)	-1.69
Solubility	4.49e+00 mg/ml ; 2.07e-02 mol/l
Class	Soluble

Pharmacokinetics	
GI absorption	High
BBB permeant	No
P-gp substrate	No
CYP1A2 inhibitor	Yes
CYP2C19 inhibitor	No
CYP2C9 inhibitor	No
CYP2D6 inhibitor	No
CYP3A4 inhibitor	No
Log K_p (skin permeation)	-6.57 cm/s

Druglikeness	
Lipinski	Yes; 0 violation
Ghose	Yes
Veber	Yes
Egan	Yes
Muegge	Yes
Bioavailability Score	0.85

Medicinal Chemistry	
PAINS	0 alert
Brenk	2 alerts: michael_acceptor_1, michael_acceptor_4
Leadlikeness	No; 1 violation: MW<250
Synthetic accessibility	3.50

Figure S13. SwissADME prediction for compounds **1–11** and positive controls (cont.).

(7)

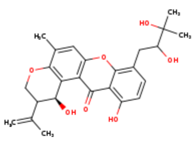

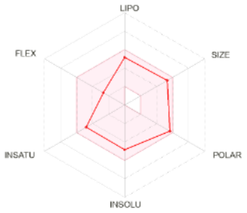

SMILES	CC(=C)C1COc2c([C@H]1O)c1c(cc2C)oc2c(c1=O)c(O)ccc2CC(C(O)(C)C)O

Physicochemical Properties	
Formula	C25H28O7
Molecular weight	440.49 g/mol
Num. heavy atoms	32
Num. arom. heavy atoms	14
Fraction Csp3	0.40
Num. rotatable bonds	4
Num. H-bond acceptors	7
Num. H-bond donors	4
Molar Refractivity	122.79
TPSA	120.36 Å²

Lipophilicity	
Log $P_{o/w}$ (iLOGP)	3.39
Log $P_{o/w}$ (XLOGP3)	3.51
Log $P_{o/w}$ (WLOGP)	2.93
Log $P_{o/w}$ (MLOGP)	1.13
Log $P_{o/w}$ (SILICOS-IT)	4.38
Consensus Log $P_{o/w}$	3.07

Water Solubility	
Log *S* (ESOL)	-4.84
Solubility	6.34e-03 mg/ml ; 1.44e-05 mol/l
Class	Moderately soluble
Log *S* (Ali)	-5.72
Solubility	8.37e-04 mg/ml ; 1.90e-06 mol/l
Class	Moderately soluble
Log *S* (SILICOS-IT)	-5.48
Solubility	1.47e-03 mg/ml ; 3.33e-06 mol/l
Class	Moderately soluble

Pharmacokinetics	
GI absorption	High
BBB permeant	No
P-gp substrate	Yes
CYP1A2 inhibitor	No
CYP2C19 inhibitor	No
CYP2C9 inhibitor	No
CYP2D6 inhibitor	No
CYP3A4 inhibitor	Yes
Log K_p (skin permeation)	-6.49 cm/s

Druglikeness	
Lipinski	Yes; 0 violation
Ghose	Yes
Veber	Yes
Egan	Yes
Muegge	Yes
Bioavailability Score	0.55

Medicinal Chemistry	
PAINS	0 alert
Brenk	2 alerts: isolated_alkene, polycyclic_aromatic_hydrocarbon_2
Leadlikeness	No; 2 violations: MW>350, XLOGP3>3.5
Synthetic accessibility	5.11

(8)

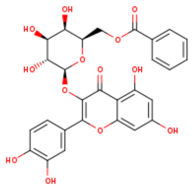

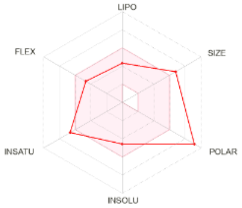

SMILES	Oc1cc(O)c2c(c1)oc(c(c2=O)O[C@@H]1O[C@H](COC(=O)c2ccccc2)[C@@H]([C@@H]([C@H]1O)O)O)c1ccc(c(c1)O)O

Physicochemical Properties	
Formula	C28H24O13
Molecular weight	568.48 g/mol
Num. heavy atoms	41
Num. arom. heavy atoms	22
Fraction Csp3	0.21
Num. rotatable bonds	7
Num. H-bond acceptors	13
Num. H-bond donors	7
Molar Refractivity	139.80
TPSA	216.58 Å²

Lipophilicity	
Log $P_{o/w}$ (iLOGP)	2.61
Log $P_{o/w}$ (XLOGP3)	2.04
Log $P_{o/w}$ (WLOGP)	1.33
Log $P_{o/w}$ (MLOGP)	-1.09
Log $P_{o/w}$ (SILICOS-IT)	0.92
Consensus Log $P_{o/w}$	1.16

Water Solubility	
Log *S* (ESOL)	-4.58
Solubility	1.48e-02 mg/ml ; 2.60e-05 mol/l
Class	Moderately soluble
Log *S* (Ali)	-6.22
Solubility	3.45e-04 mg/ml ; 6.08e-07 mol/l
Class	Poorly soluble
Log *S* (SILICOS-IT)	-4.17
Solubility	3.83e-02 mg/ml ; 6.75e-05 mol/l
Class	Moderately soluble

Pharmacokinetics	
GI absorption	Low
BBB permeant	No
P-gp substrate	No
CYP1A2 inhibitor	No
CYP2C19 inhibitor	No
CYP2C9 inhibitor	No
CYP2D6 inhibitor	No
CYP3A4 inhibitor	No
Log K_p (skin permeation)	-8.32 cm/s

Druglikeness	
Lipinski	No; 3 violations: MW>500, NorO>10, NHorOH>5
Ghose	No; 2 violations: MW>480, MR>130
Veber	No; 1 violation: TPSA>140
Egan	No; 1 violation: TPSA>131.6
Muegge	No; 3 violations: TPSA>150, H-acc>10, H-don>5
Bioavailability Score	0.17

Medicinal Chemistry	
PAINS	1 alert: catechol_A
Brenk	1 alert: catechol
Leadlikeness	No; 1 violation: MW>350
Synthetic accessibility	5.75

Figure S13. SwissADME prediction for compounds **1–11** and positive controls (cont.).

(11)

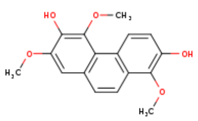

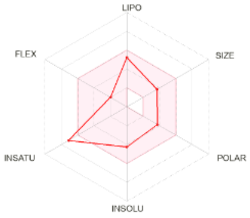

SMILES COc1cc2ccc3c(c2c(c1O)OC)ccc(c3OC)O

Physicochemical Properties	
Formula	C17H16O5
Molecular weight	300.31 g/mol
Num. heavy atoms	22
Num. arom. heavy atoms	14
Fraction Csp3	0.18
Num. rotatable bonds	3
Num. H-bond acceptors	5
Num. H-bond donors	2
Molar Refractivity	84.98
TPSA	68.15 Å²
**Lipophilicity**	
Log $P_{o/w}$ (iLOGP)	2.70
Log $P_{o/w}$ (XLOGP3)	3.63
Log $P_{o/w}$ (WLOGP)	3.43
Log $P_{o/w}$ (MLOGP)	1.78
Log $P_{o/w}$ (SILICOS-IT)	3.10
Consensus Log $P_{o/w}$	2.93

Water Solubility	
Log *S* (ESOL)	-4.26
Solubility	1.64e-02 mg/ml ; 5.47e-05 mol/l
Class	Moderately soluble
Log *S* (Ali)	-4.75
Solubility	5.35e-03 mg/ml ; 1.78e-05 mol/l
Class	Moderately soluble
Log *S* (SILICOS-IT)	-4.97
Solubility	3.21e-03 mg/ml ; 1.07e-05 mol/l
Class	Moderately soluble
**Pharmacokinetics**	
GI absorption	High
BBB permeant	Yes
P-gp substrate	No
CYP1A2 inhibitor	Yes
CYP2C19 inhibitor	Yes
CYP2C9 inhibitor	Yes
CYP2D6 inhibitor	Yes
CYP3A4 inhibitor	Yes
Log K_p (skin permeation)	-5.55 cm/s
**Druglikeness**	
Lipinski	Yes; 0 violation
Ghose	Yes
Veber	Yes
Egan	Yes
Muegge	Yes
Bioavailability Score	0.55
**Medicinal Chemistry**	
PAINS	0 alert
Brenk	1 alert: polycyclic_aromatic_hydrocarbo
Leadlikeness	No; 1 violation: XLOGP3>3.5
Synthetic accessibility	2.00

UA

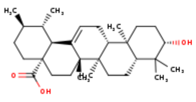

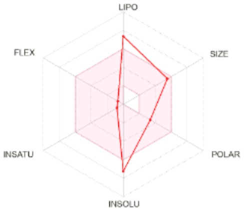

SMILES C[C@@H]1CC[C@]2([C@@H]([C@H]1C)C1=CC[C@H]3[C@@]([C@@]1(CC2)C)(C)CC[C@@H]1[C@]3(C)CC[C@@H](C1(C)C)O)C(=O)O

Physicochemical Properties	
Formula	C30H48O3
Molecular weight	456.70 g/mol
Num. heavy atoms	33
Num. arom. heavy atoms	0
Fraction Csp3	0.90
Num. rotatable bonds	1
Num. H-bond acceptors	3
Num. H-bond donors	2
Molar Refractivity	136.91
TPSA	57.53 Å²
**Lipophilicity**	
Log $P_{o/w}$ (iLOGP)	3.95
Log $P_{o/w}$ (XLOGP3)	7.34
Log $P_{o/w}$ (WLOGP)	7.09
Log $P_{o/w}$ (MLOGP)	5.82
Log $P_{o/w}$ (SILICOS-IT)	5.46
Consensus Log $P_{o/w}$	5.93

Water Solubility	
Log *S* (ESOL)	-7.23
Solubility	2.69e-05 mg/ml ; 5.89e-08 mol/l
Class	Poorly soluble
Log *S* (Ali)	-8.38
Solubility	1.92e-06 mg/ml ; 4.21e-09 mol/l
Class	Poorly soluble
Log *S* (SILICOS-IT)	-5.67
Solubility	9.72e-04 mg/ml ; 2.13e-06 mol/l
Class	Moderately soluble
**Pharmacokinetics**	
GI absorption	Low
BBB permeant	No
P-gp substrate	No
CYP1A2 inhibitor	No
CYP2C19 inhibitor	No
CYP2C9 inhibitor	No
CYP2D6 inhibitor	No
CYP3A4 inhibitor	No
Log K_p (skin permeation)	-3.87 cm/s
**Druglikeness**	
Lipinski	Yes; 1 violation: MLOGP>4.15
Ghose	No; 3 violations: WLOGP>5.6, MR>130, #atoms>70
Veber	Yes
Egan	No; 1 violation: WLOGP>5.88
Muegge	No; 1 violation: XLOGP3>5
Bioavailability Score	0.85
**Medicinal Chemistry**	
PAINS	0 alert
Brenk	1 alert: isolated_alkene
Leadlikeness	No; 2 violations: MW>350, XLOGP3>3.5
Synthetic accessibility	6.21

Figure S13. SwissADME prediction for compounds **1–11** and positive controls (cont.).

T

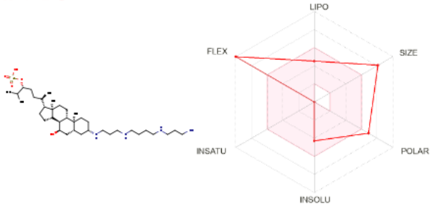

SMILES NCCCNCCCCNCCCN[C@H]1CC[C@]2([C@H](C1)C[C@H]([C@@H]1[C@@H]2CC[C@]2([C@H]1CC[C@@H]2[C@@H](CC[C@H](C(C)C)OS(=O)(=O)O)C)C)O)C

Physicochemical Properties	
Formula	C37H72N4O5S
Molecular weight	685.06 g/mol
Num. heavy atoms	47
Num. arom. heavy atoms	0
Fraction Csp3	1.00
Num. rotatable bonds	20
Num. H-bond acceptors	9
Num. H-bond donors	6
Molar Refractivity	194.89
TPSA	154.32 Å²

Lipophilicity	
Log $P_{o/w}$ (iLOGP)	5.13
Log $P_{o/w}$ (XLOGP3)	2.42
Log $P_{o/w}$ (WLOGP)	6.61
Log $P_{o/w}$ (MLOGP)	3.46
Log $P_{o/w}$ (SILICOS-IT)	4.83
Consensus Log $P_{o/w}$	4.49

Water Solubility	
Log *S* (ESOL)	-4.29
Solubility	3.50e-02 mg/ml ; 5.11e-05 mol/l
Class	Moderately soluble
Log *S* (Ali)	-5.30
Solubility	3.41e-03 mg/ml ; 4.98e-06 mol/l
Class	Moderately soluble
Log *S* (SILICOS-IT)	-7.79
Solubility	1.12e-05 mg/ml ; 1.63e-08 mol/l
Class	Poorly soluble

Pharmacokinetics	
GI absorption	Low
BBB permeant	No
P-gp substrate	No
CYP1A2 inhibitor	No
CYP2C19 inhibitor	No
CYP2C9 inhibitor	No
CYP2D6 inhibitor	No
CYP3A4 inhibitor	Yes
Log K_p (skin permeation)	-8.76 cm/s

Druglikeness	
Lipinski	No; 2 violations: MW>500, NHorOH>5
Ghose	No; 4 violations: MW>480, WLOGP>5.6, MR>130, #atoms>70
Veber	No; 2 violations: Rotors>10, TPSA>140
Egan	No; 2 violations: WLOGP>5.88, TPSA>131.
Muegge	No; 4 violations: MW>600, TPSA>150, Rotors>15, H-don>5
Bioavailability Score	0.17

Medicinal Chemistry	
PAINS	0 alert
Brenk	1 alert: sulfonic_acid_2
Leadlikeness	No; 2 violations: MW>350, Rotors>7
Synthetic accessibility	7.51

E

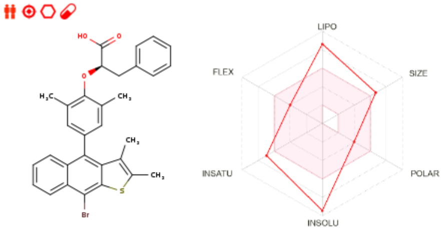

SMILES OC(=O)[C@H](Oc1c(C)cc(cc1C)c1c2ccccc2c(c2c1c(C)c(s2)C)Br)Cc1ccccc1

Physicochemical Properties	
Formula	C31H27BrO3S
Molecular weight	559.51 g/mol
Num. heavy atoms	36
Num. arom. heavy atoms	25
Fraction Csp3	0.19
Num. rotatable bonds	6
Num. H-bond acceptors	3
Num. H-bond donors	1
Molar Refractivity	154.70
TPSA	74.77 Å²

Lipophilicity	
Log $P_{o/w}$ (iLOGP)	4.00
Log $P_{o/w}$ (XLOGP3)	9.56
Log $P_{o/w}$ (WLOGP)	8.79
Log $P_{o/w}$ (MLOGP)	6.12
Log $P_{o/w}$ (SILICOS-IT)	9.99
Consensus Log $P_{o/w}$	7.69

Water Solubility	
Log *S* (ESOL)	-9.45
Solubility	1.99e-07 mg/ml ; 3.55e-10 mol/l
Class	Poorly soluble
Log *S* (Ali)	-11.04
Solubility	5.08e-09 mg/ml ; 9.08e-12 mol/l
Class	Insoluble
Log *S* (SILICOS-IT)	-11.77
Solubility	9.50e-10 mg/ml ; 1.70e-12 mol/l
Class	Insoluble

Pharmacokinetics	
GI absorption	Low
BBB permeant	No
P-gp substrate	Yes
CYP1A2 inhibitor	No
CYP2C19 inhibitor	No
CYP2C9 inhibitor	No
CYP2D6 inhibitor	Yes
CYP3A4 inhibitor	Yes
Log K_p (skin permeation)	-2.93 cm/s

Druglikeness	
Lipinski	No; 2 violations: MW>500, MLOGP>4.15
Ghose	No; 3 violations: MW>480, WLOGP>5.6, MR>130
Veber	Yes
Egan	No; 1 violation: WLOGP>5.88
Muegge	No; 1 violation: XLOGP3>5
Bioavailability Score	0.85

Medicinal Chemistry	
PAINS	0 alert
Brenk	0 alert
Leadlikeness	No; 2 violations: MW>350, XLOGP3>3.5
Synthetic accessibility	4.24

Figure S13. SwissADME prediction for compounds **1–11** and positive controls (cont.).

Metformin

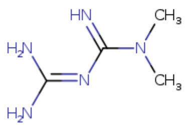

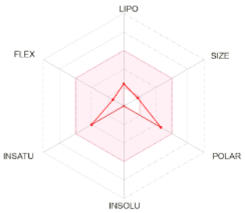

SMILES CN(C(=N)N=C(N)N)C

Physicochemical Properties	
Formula	C4H11N5
Molecular weight	129.16 g/mol
Num. heavy atoms	9
Num. arom. heavy atoms	0
Fraction Csp3	0.50
Num. rotatable bonds	2
Num. H-bond acceptors	2
Num. H-bond donors	3
Molar Refractivity	36.93
TPSA	91.49 Å²

Lipophilicity	
Log $P_{o/w}$ (iLOGP)	0.34
Log $P_{o/w}$ (XLOGP3)	-1.27
Log $P_{o/w}$ (WLOGP)	-1.24
Log $P_{o/w}$ (MLOGP)	-0.56
Log $P_{o/w}$ (SILICOS-IT)	-1.74
Consensus Log $P_{o/w}$	-0.89

Water Solubility	
Log *S* (ESOL)	0.29
Solubility	2.53e+02 mg/ml ; 1.96e+00 mol/l
Class	Highly soluble
Log *S* (Ali)	-0.15
Solubility	9.05e+01 mg/ml ; 7.00e-01 mol/l
Class	Very soluble
Log *S* (SILICOS-IT)	0.58
Solubility	4.90e+02 mg/ml ; 3.79e+00 mol/l
Class	Soluble

Pharmacokinetics	
GI absorption	High
BBB permeant	No
P-gp substrate	No
CYP1A2 inhibitor	No
CYP2C19 inhibitor	No
CYP2C9 inhibitor	No
CYP2D6 inhibitor	No
CYP3A4 inhibitor	No
Log K_p (skin permeation)	-7.99 cm/s

Druglikeness	
Lipinski	Yes; 0 violation
Ghose	No; 3 violations: MW<160, WLOGP<-0.4, MR<40
Veber	Yes
Egan	Yes
Muegge	No; 2 violations: MW<200, #C<5
Bioavailability Score	0.55

Medicinal Chemistry	
PAINS	0 alert
Brenk	2 alerts: imine_1, imine_2
Leadlikeness	No; 1 violation: MW<250
Synthetic accessibility	3.02

Figure S13. SwissADME prediction for compounds **1–11** and positive controls (cont.).

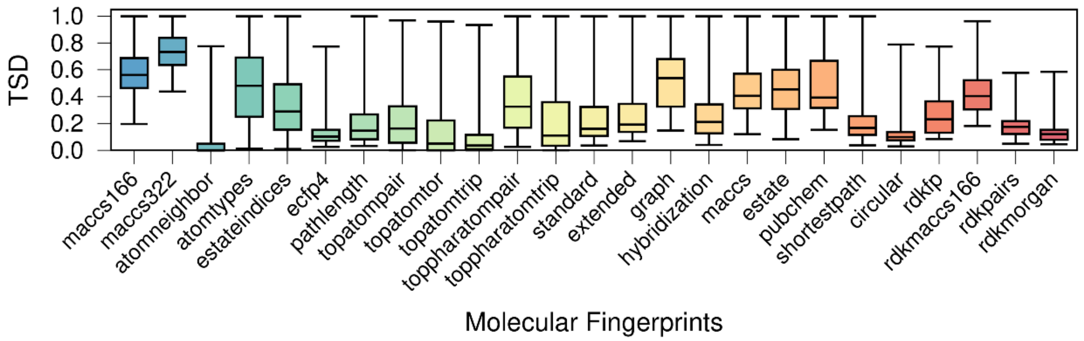

Figure S14. 25 molecular fingerprints computed for the 47 most active NPs to build the SAS map between the evaluated compounds and UA.

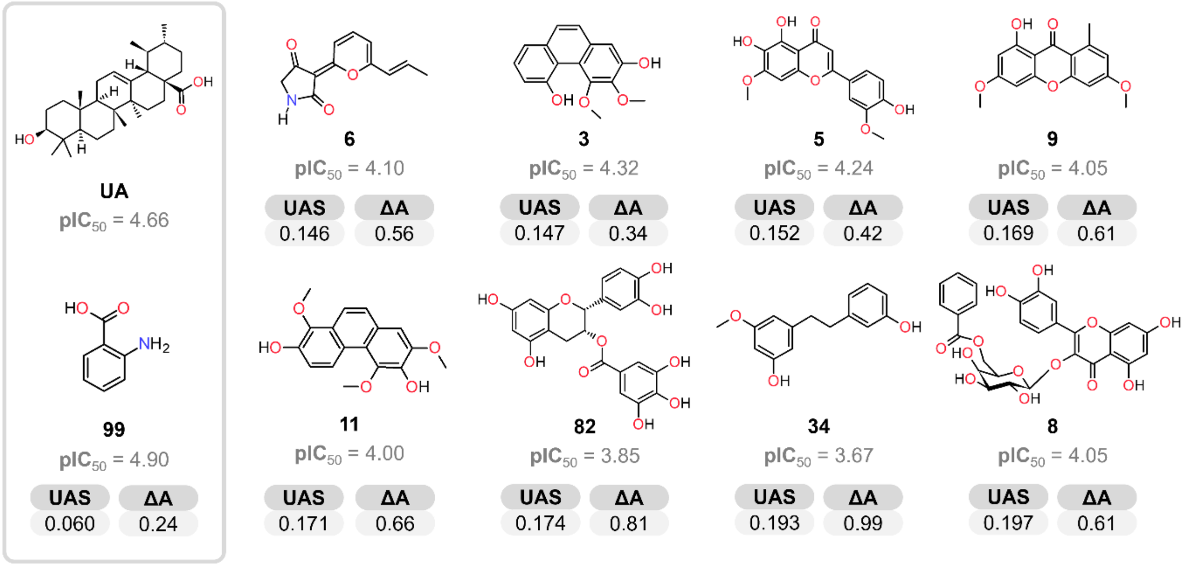

Figure S15. Chemical structures of the compounds identified as **scaffold hops** in the map constructed with the tested compounds.

The structure similarity and activity difference values of these compounds with the ursolic acid are shown below the structures.
